# Robustness, spatial detail, and pitfalls of fixed ICA dimensionality in resting-state fMRI networks at 1.5, 3, and 7 T

**DOI:** 10.3389/fnins.2025.1731143

**Published:** 2026-01-06

**Authors:** Pierfrancesco Ambrosi, Marta Lancione, Paolo Cecchi, Michela Tosetti, Laura Biagi

**Affiliations:** 1 Laboratory of Medical Physics and Magnetic Resonance, IRCCS Stella Maris Foundation, Pisa, Italy; 2 Imago7 Research Foundation, Pisa, Italy; 3 Department of Translational Research on New Technologies in Medicine and Surgery, University of Pisa, Pisa, Italy

**Keywords:** BOLD fMRI, resting state fMRI (rsfMRI), brain connectivity, functional connectivity (FC), independent component analyses (ICA)

## Abstract

Resting-state fMRI functional connectivity analysis is usually performed with seed-based methods that strongly rely on user-dependent definitions of regions of interest. Data-driven methods like independent component analysis (ICA) can mitigate this need. However, the number of components that should be expected in an fMRI acquisition, which determines the model order of the ICA, is not defined, and it is not uniformly chosen across studies. This variability is further complicated by the dependence of component number on field strength, with higher field strengths typically yielding more detectable components. Therefore, relying on a predetermined number may influence the results. Here, we compare functional maps obtained through ICA analysis at different magnetic field strengths and at various levels of spatial detail. Our results confirm the presence of the most frequently reported resting-state networks across field strengths and demonstrate that higher magnetic field strength enables more robust detection of functional networks with greater spatial detail. We also show that: (1) fixing the number of components, although improving interpretability of group results, may provide an incomplete picture of brain function; (2) a greater number of components is consistently identified at higher field strength, suggesting that the model order should be adapted according to both field strength and spatial detail.

## Introduction

Resting-state BOLD functional Magnetic Resonance Imaging (rs-fMRI) (Biswal et al., 1995; Azeez and Biswal, 2017; Smith et al., 2013; Raimondo et al., 2021; Van Den Heuvel and Hulshoff Pol, 2010; Chen et al., 2020) is a widely used experimental procedure that allows exploring brain activity during rest by identifying functionally connected brain regions in the absence of a coherent external stimulus. Resting-state networks (RSNs) are usually, but not exclusively, individuated by defining a Region of Interest (ROI), which can be either a single voxel or a larger brain region, either functionally or anatomically defined (Azeez and Biswal, 2017; Van Den Heuvel and Hulshoff Pol, 2010). Functionally related areas are usually found by comparing the seed’s time course to the rest of the brain or portions of it, generally by means of temporal correlation or analogous measures (Azeez and Biswal, 2017). This procedure has provided numerous insights into brain functional networks (Yang et al., 2020), but has the drawback of user-dependent identification of an ROI, which can lead to biased results or an incomplete description of the brain as a whole (Smith et al., 2013). Data-driven methods such as independent component analysis (ICA) (Beckmann, 2012; Kiviniemi et al., 2003) might provide a solution to this issue, as functional networks are individuated by considering the whole brain and with minimal user-dependent assumptions.

ICA has been frequently employed in rs-fMRI, either to detect functional networks or noise spatial patterns, and has overall confirmed findings previously obtained using other methods (Damoiseaux et al., 2006; Smith et al., 2009). A crucial parameter of ICA is the model order, that is, the number of components into which the original dataset is decomposed, for which no standardized criterion exists. Choosing a low number of components has the clear advantage of aiding visualization and interpretability of the results. However, it imposes strong prior assumptions that may obscure individual or group differences, particularly those influenced by factors such as age or pathology that might affect the number and the characteristics of the components (Calhoun, 2009; Du et al., 2015; Hyatt et al., 2015; Öngür et al., 2010; Sorg et al., 2007; Syed et al., 2017; Xiong et al., 2020). On the other hand, assuming too many components might provide meaningless results by forcing functionally connected areas to split (Calhoun et al., 2009; Li et al., 2007; Abou-Elseoud et al., 2010). ICA toolboxes generally allow the automatic estimation of the optimal number of components in a dataset, which depends on the number of acquired volumes (Beckmann, 2012; Damoiseaux et al., 2006; Calhoun and De Lacy, 2017). This would further reduce user-driven assumptions, but is not always implemented, as direct comparison between groups, individuals, or studies can become impractical (Calhoun and De Lacy, 2017).

Usually, 20 to 40 independent components are assumed in the resting brain (Smith et al., 2009; Calhoun and De Lacy, 2017; Cole et al., 2010; Laird et al., 2011), independently of field strength and spatial detail, although both field strength and spatial detail can be expected to influence the number of detectable functional networks (Hu et al., 2016; Kiviniemi et al., 2009). As high-field scanners that achieve higher levels of spatial detail become more common, it is relevant to understand their added value in network detection via ICA decomposition. Increasing field strength amplifies the signal-to-noise ratio, which allows the acquisition of fMRI data at higher spatial resolution (Triantafyllou et al., 2005; Van Der Zwaag et al., 2009). Higher spatial resolution can increase the number of components, for example, by separating networks into more subnetworks. On the contrary, with small voxel sizes, noise can dominate over the signal, impeding the discrimination of functional networks and noise components. The optimal number of components can then be expected to increase with higher magnetic fields strength and spatial resolution. Therefore, determining how the model order should be adapted as these acquisition parameters change is important to prevent biologically implausible decompositions that could compromise the validity (Zuo et al., 2019) of conclusions on the brain architecture, both in healthy subjects ad patients. To address how the expected number of components should be adapted based on field strength and spatial resolution is important to avoid biologically implausible models resulting in invalid conclusions on the brain architecture.

Here, we explore the effect of automatic dimensionality estimation of ICA on rs-fMRI acquired at different field strengths, 1.5, 3, and 7 T, in a traveling brain experiment, within a group of healthy subjects. Our aim is to investigate how the number of components is affected by magnetic field strength and spatial resolution, as well as the differences between automatic and fixed dimensionality analyses.

## Methods

### MRI protocol

Thirteen healthy volunteers (age 31–60, 7 females) with no history of neurological or psychiatric disorders participated in the study and were scanned at different field strengths after giving their written informed consent. Acquisitions were conducted on a 1.5 T GE HDxt, a GE SIGNA Premier 3T, and a GE SIGNA 7 T (GE HealthCare). The acquisition protocols included anatomical and resting-state BOLD-fMRI acquisitions. The protocols were established in order to achieve an optimal protocol for each magnetic field used, by adjusting the acquisition parameters to the best extent allowed by each system, while keeping the total time of acquisition constant (6 min).

During rs-fMRI, participants lied supine inside the scanner with eyes closed, and were instructed not to think about anything in particular. For T1-weighted images, we used 3D sequences with isotropic spatial resolutions: Fast SPoiled GRadient echo (FSPGR) at 1.5 T and 3 T (BRAin VOlume imaging, BRAVO), and Magnetization-Prepared RApid Gradient Echo (MPRAGE) at 7 T. Detailed sequence parameters are reported in Table 1. fMRI data were acquired by using 2D GRE-EPI sequences at all magnetic field strengths (details in Table 2). At 3 T and 7 T, we acquired an additional 2D GRE-EPI with the same parameters as reported in Table 2 with inverted phase-encoding polarity to correct geometric distortions. Dummy volumes were acquired at the beginning of each fMRI acquisition.

**Table 1:** T1-weighted sequence parameters for each magnetic field strength.

Acquisition parameters	1.5 T	3 T	7 T
Sequence	3D FSPGR	3D BRAVO	3D MPRAGE
Isotropic spatial resolution (mm)	1	0.9	0.7
FOV (mm^3^)	256 × 256 × 164	230 × 230 × 176	240 × 240 × 164
Matrix size	256 × 256 × 164	256 × 256 × 196	320 × 320 × 234
TR/TE/TI (ms)	12.4/5.2/700	7.3/3/500	3560/3.9/1100
FA (°)	10	8	8
Pixel BW (Hz)	122	122	244

**Table 2:** fMRI sequence parameters at each magnetic field strength.

Acquisition parameters	1.5 T	3 T	7 T
Sequence	2D EPI-GRE	2D EPI-GRE*	2D EPI-GRE*
Isotropic spatial resolution (mm)	3.0	2.2	1.8
FOV (mm^3^)	256 × 256 × 120	240 × 240 × 148	230 × 230 × 144
Matrix size	64 × 64 × 40	110 × 110 × 67	128 × 128 × 80
TR/TE (ms)	3,000/50	2,000/24	2,000/21.6
FA (°)	90	90	70
Acceleration factor	ASSET = 2	ARC = 2 Multiband = 2	ARC = 3 Multiband = 2
Number of volumes	120	180	180
Acquisition time	6 min	6 min	6 min

Sequences labelled with an asterisk were also acquired with reversed polarity to correct geometric distortions (4 volumes each). Dummy volumes were acquired at the beginning of each sequence.

### Preprocessing

Bias field correction using N4 algorithm and brain extraction were performed using the Advance Normalization Tools (ANTs) algorithm (Tustison et al., 2010) on T1-weighted anatomical images, which were then registered to the MNI template using linear and non-linear registration in AFNI (Cox, 1996; Cox and Hyde, 1997). Anatomical data were then segmented into grey matter (GM), white matter (WM), and Cerebrospinal Fluid (CSF) using 3dSeg in AFNI. On functional data, we performed despiking, slice-timing correction, and a two-step motion correction by first determining the volume with minimal displacement from the average time series and then registering all time points to the selected volume. Brain extraction was then performed using the BET algorithm in FSL (Smith, 2002). EPI images were corrected for geometric distortions (only for 3 T and 7 T data) using AFNI 3dQwarp and then aligned to the corresponding T1-weighted data. Spatially aligned data were then smoothed to FWHMs of 4, 6, 8, and 12 mm using 3dBlurToFWHM in AFNI and scaled to percent signal change. This range of smoothing levels was chosen because it covers the values that are usually used for the field strengths employed here (Smith et al., 2009; Triantafyllou et al., 2006). As physiological and thermal noise are affected differently by smoothing (Triantafyllou et al., 2006), these datasets were regarded as an approximation of images with different spatial resolutions. While such datasets cannot be considered independent from each other, synthesizing such images from the same scan allows us to compare ICA results without introducing additional confounding factors, like motion or individual differences across scanning sessions (Maknojia et al., 2019). Finally, regression of nuisance time courses included the 6 motion degrees of freedom and the average WM and CSF signal. WM and CSF time courses were generated from the signal averaged over the segmentation masks obtained from the T1-weighted image, after resampling to the spatial resolution of the corresponding fMRI data. Time-courses were then pass-band filtered (0.01–0.1 Hz).

### Data analysis

ICA was performed using MELODIC in FSL (Beckmann and Smith, 2004), at the group level [group-ICA, gICA (Du et al., 2015; Calhoun et al., 2009)], for all levels of smoothing and field strengths separately. Dimensionality, that is, the number of components, was either fixed to 20 or estimated automatically by MELODIC. The model order of 20 was chosen in accordance with previous studies (as in Smith et al., 2009; Laird et al., 2011 for 3 T data). gICA probability maps were clusterized in AFNI with a second nearest-neighbors (face and edges) minimum cluster size of 80, after thresholding to 0.95. Spatial similarity between components was estimated through Pearson’s spatial correlation to address the robustness of ICA networks at different levels of spatial detail (smoothing). For each field strength, gICAs with the highest level of smoothing (12 mm-FWHM), with either fixed or automatic dimensionality, were used as benchmarks for gICA obtained under different conditions (lower level of smoothing and/or automatic dimensionality). This allows us to assess the number of components that are consistently identified across different levels of spatial detail, and how fixed or automatic dimensionality affects the resulting spatial patterns. The underlying rationale is that increased smoothing enhances SNR but reduces spatial detail, diminishing differences across field strengths. Therefore, we selected a reference template and evaluated how many components matched the template when smoothing was reduced or when dimensionality was switched from fixed to automatic. Table 3 summarizes the experiments performed in this work, i.e., the comparisons between each template (presented in the columns) and the test set of ICAs (in the rows). Each comparison, labelled as Experiment 1 to 5, results in a correlation matrix for each magnetic field strength and level of smoothing. The dimension of each correlation matrix depends on the number of components in the test and template and is indicated in Table 3. When the ICA dimensionality was free to vary, the number of resulting components in the template and test sets is indicated in the table as M*_ij_* and N*_ij_*, respectively, where *i* represents the levels of smoothing and *j* the magnetic field strengths. In all cases, a component in the test set was considered matched to a component in the template if the spatial correlation between the corresponding t-score maps was higher than 0.25 (Laird et al., 2011). The temporal Signal-to-Noise Ratio (tSNR) was estimated voxel-wise using AFNI as the ratio of the mean signal to its standard deviation over time, and then averaged across the skull-stripped mask of the whole brain.

**Table 3:** Summary of the comparisons between different gICA.

Test	Template			
	gICA DIM = 20 Smoothing = 12 mm	gICA DIM = auto Smoothing = 12 mm	gICA DIM = 20 Smoothing = 4, 6, 8, 12 mm	gICA DIM = auto Smoothing = 4, 6, 8, 12 mm
gICA DIM = 20 Smoothing = 4, 6, 8 mm	**Experiment 1** 3 field × 3 smoothing 20 × 20 correlation matrices			
gICA DIM = auto Smoothing = 4, 6, 8, 12 mm	**Experiment 2** 12 N*_ij_* × 20 correlation matrices			
gICA DIM = auto Smoothing = 4, 6, 8 mm		**Experiment 3** 3 field × 3 smoothing N*_ij_* × M*_ij_* correlation matrices		
ssICA DIM = 20 Smoothing = 4, 6, 8, 12 mm			**Experiment 4** S*_j_* × 3 fields 20 × 20 correlation matrices	
ssICA DIM = auto Smoothing = 4, 6, 8, 12 mm				**Experiment 5** S*_j_* × 3 fields N*_ij_* × M*_ij_* Correlation matrices

Columns indicate the templates used for the comparisons, their model order, either fixed to 20 (DIM = 20) or automatic (DIM = auto), and their smoothing level. Rows indicate the test set of gICA, their model order, and the smoothing levels. Each comparison, labelled as Experiments 1 to 4, results in a correlation matrix for each level of smoothing and field strength, with dimensions depending on the number of components in the template, M*_ij_*, and in the test, N*_ij_*, where *i* = levels of smoothing (4, 6, 8, and 12 mm) and *j* = field strength (1.5, 3, and 7 T).

## Results

**Figure 1:**
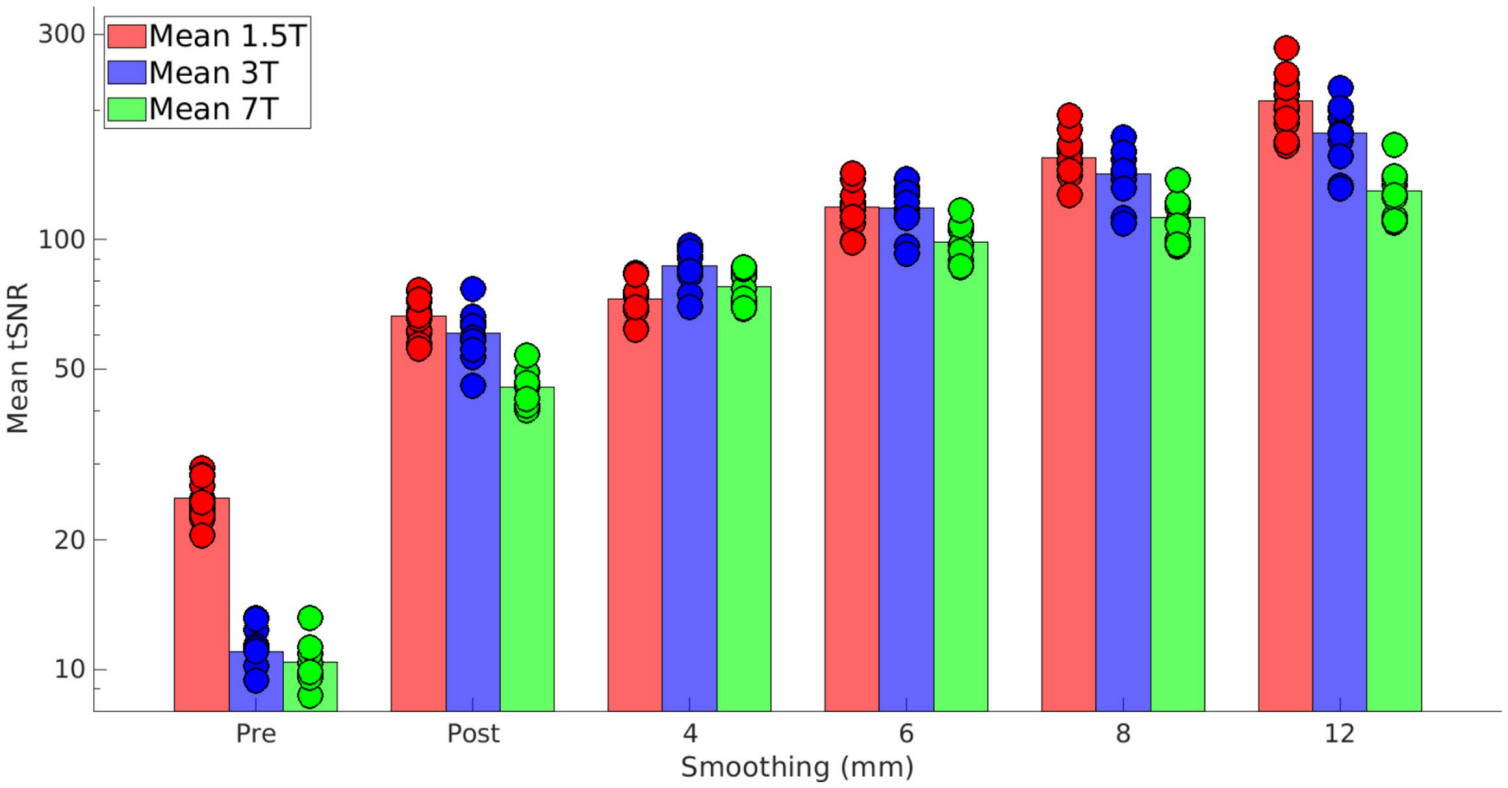
Bar plot of tSNR calculated before (“Pre”) and after (“Post”) pre-processing and after smoothing to 4, 6, 8, and 12 mm. Bars show the average across participants, in red, blue, and green for 1.5 T, 3 T and 7 T, respectively; circles represent values for each participant. Before pre-processing, 1.5 T has higher tSNR than higher fields, because of the larger voxel size. Pre-processing improves tSNR for all fields and participants and almost completely compensates for the aforementioned difference. Smoothing improves tSNR even further, as expected. While 7 T data show slightly lower tSNR than lower fields, our dataset can be regarded as fairly homogeneous in data quality.

At the end of the study, among the total of 13 recruited healthy participants, 11 were scanned at 1.5 T (6 females), 10 at 3 T (5 females), and 9 at 7 T (4 females). Figure 1 shows, for each magnetic field strength, the tSNR for each subject before (“Pre”) and after (“Post”) pre-processing, and after smoothing at 4, 6, 8, and 12 mm. These data show that increasing the magnetic field strength allows for obtaining data with comparable SNR and smaller voxel sizes (higher spatial resolution). On raw data, SNR is higher at 1.5 T, but this difference is mitigated after data pre-processing, even before smoothing. As expected, smoothing improved tSNR even further, which remained slightly lower at 7 T than at lower fields. This demonstrates that the quality of our dataset is fairly homogeneous across field strength.

### Experiment 1

**Figure 2:**
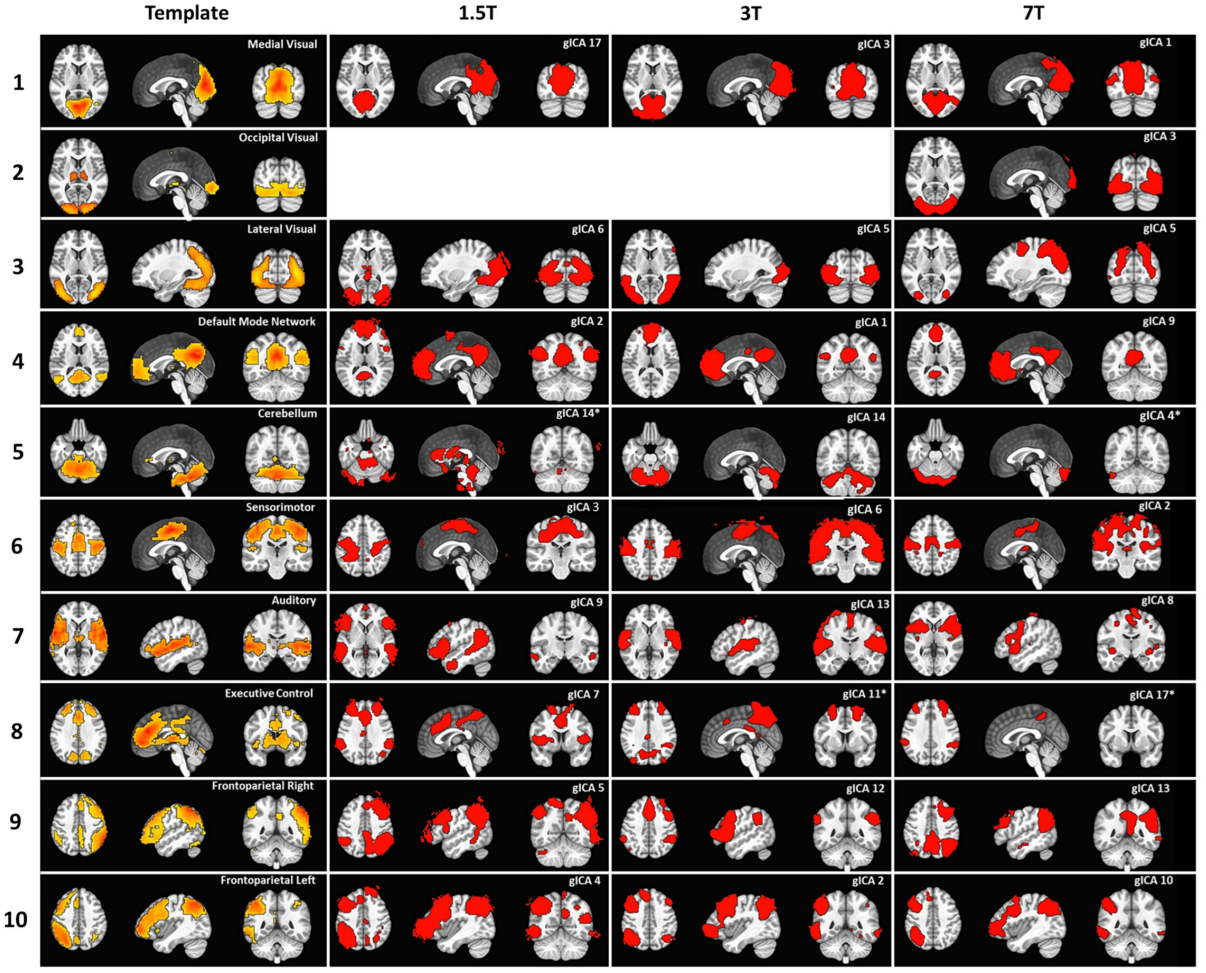
Results of the comparison between the RSN template from Smith et al. (2009) and gICA at different field strengths, obtained with 12 mm smoothing and dimensionality fixed to 20. Columns from left to right: template components, displayed in the original order, best matching components at 1.5, 3, and 7 T. The top right corner of each panel displays the RSN names for the template or the component number assigned by MELODIC. The asterisks denote components that, while representing the best match for the template, have a correlation coefficient lower than 0.25.

The set of the 10 major RSNs identified in Smith et al. (2009), which resulted from the analysis of 36 subjects during resting-state at 3 T, was used as a template. The gICA for each field strength with 12 mm smoothing and dimensionality fixed to 20 was compared to this template via spatial correlation. Each RSN of the template was matched to the gICA component showing the highest spatial correlation. Most of the RSNs of the template were identified in the gICA for all field strengths, as shown in Figure 2.

At 1.5 T, the occipital visual RSN (row 2) is not found in our dataset, but has likely been included in the lateral visual RSN (row 3, gICA6), which also shows some thalamic activations. The preferential matching of gICA6 to the lateral visual RSN is due to the higher spatial correlation with the lateral visual RSN (*ρ* = 0.47) than to the occipital visual RSN (ρ = 0.28), although this value was also considered significant. The Default Mode Network (DMN, row 4) is found in our dataset, but the medial frontal cluster is larger and also includes some parietal clusters. The cerebellum RSN (row 5) is best matched by gICA14, which clearly shows some cerebellar areas, but is also contaminated by noise. In fact, the correlation coefficient between these networks is lower than 0.25 (*ρ* = 0.22), and is indicated in the figure by an asterisk. The somatosensory RSN (row 6) is present in our dataset (gICA3), albeit with a smaller extension. This is also true for the auditory network (row 7), which is missing some temporal activations. The executive control RSN (row 8) is best matched by gICA7, which has very similar frontal activations, but a very different occipital spatial pattern.

At 3 T, the occipital visual RSN (row 2) has been included in the medial visual RSN (row 1) and in the lateral visual RSN (*ρ* = 0.31 and *ρ* = 0.26, respectively). The DMN best matches gICA1 (*ρ* = 0.45), but also correlates strongly with gICA15 (ρ = 0.41, shown in Supplementary figures). gICA15 has medial parietal and inferior-lateral-parietal clusters resembling those of the DMN in the template, which are larger than those in gICA1. A similar splitting involves the sensorimotor RSN (row 6) which best matches gICA6 (*ρ* = 0.46), but is also strongly correlated with gICA8 (ρ = 0.44, shown in Supplementary figures), which comprises the primary sensory cortex bilaterally, not completely covered by gICA6. The executive control RSN (row 8) matches gICA1 (*ρ* = 0.33) and gICA12 (ρ = 0.35), which, however, show a better match with the DMN (ρ = 0.45) and the frontoparietal right (row 9, ρ = 0.36), respectively. Therefore, in the figure, we reported the third best matching component, which has a correlation coefficient lower than 0.25 (ρ = 0.21), indicated by an asterisk. The right frontoparietal RSN (row 9) also matches gICA18 (*ρ* = 0.26, shown in Supplementary figures), which has large temporoparietal clusters lateralized on the right side, which are smaller in gICA12 than in the template.

At 7 T, the three visual areas are correctly matched, but the rostral area of the lateral visual RSN (row 2) has been included in the medial and occipital visual RSNs (rows 1 and 2). The DMN (gICA9, *ρ* = 0.47, row 4) is lacking the temporal clusters, which are found in gICA13 (*ρ* = 0.41, shown in Supplementary figures). The medial-parietal cluster has also a smaller extension than the template, and part of it is found in gICA1 (*ρ* = 0.25, shown in Supplementary figures). No components match the cerebellum RSN (*ρ*_max_ = 0.10); gICA4 covers the posterior cerebellum, but has a small correlation with the template (*ρ* = 0.05). The sensorimotor RSN (row 6, gICA2) is smaller than the template, and parts of it can be found in gICA5 and gICA7 (*ρ* = 0.26 and ρ = 0.29). The same is true for the auditory RSN (row 7), which is split across gICA8 (*ρ* = 0.32), gICA2 (ρ = 0.31, shown in Supplementary figures), and gICA11 (ρ = 0.28, not shown). The executive control RSN (row 8) is best matched by gICA9 (ρ = 0.40), which, however, matches better the DMN (ρ = 0.47), and then by gICA8 (ρ = 0.23), which matches the auditory RSN (ρ = 0.32). Therefore, the third best matching component, gICA17, is reported in the figure, despite its low correlation coefficient (ρ = 0.21).

To summarize, most components in the template can be identified in the gICA at all field strengths. The three visual networks tend to merge, as previously reported (Beckmann et al., 2005), and only at 7 T are successfully separated as in the template. The DMN tends to incorporate some frontal regions of the executive control RSN, while temporoparietal clusters are split across other components, especially at 7 T. The cerebellum is correctly identified only at 3 T with this configuration, while at 7 T, none of the 20 components fully cover the cerebellar region. The somatosensory and auditory components are also split across components. The executive control RSN appears as the most challenging to detect, as portions of it are included in the DMN or in the right frontoparietal RSN at all field strengths. The two frontoparietal networks are the easiest to match unambiguously because of their strong lateralization.

Overall, these results confirm the presence of conventional RSNs in our dataset, but also show that an unambiguous interpretation of the resulting components is not straightforward, due to merging and splitting components. Moreover, our results at 3 T also deviate from the template, suggesting additional variability even with matching magnetic field strength and spatial resolution. This is likely due to the lower number of participants included in our study with respect to the external template, resulting in a different relevance of noise and true components in the group analysis (Xu et al., 2025).

### Experiment 2

**Figure 3:**
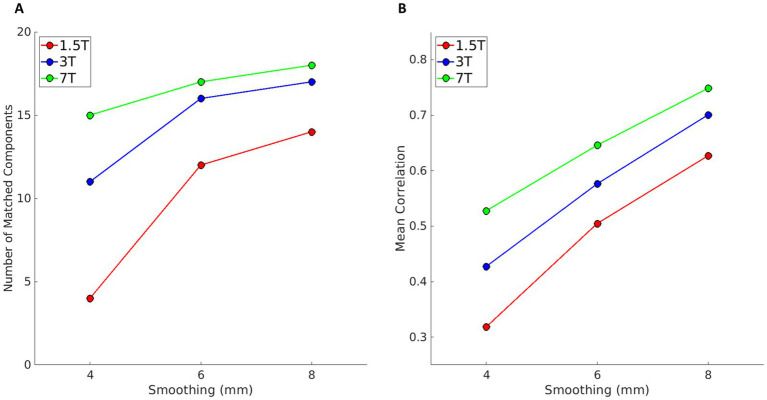
Results of Experiment 2, i.e., the comparison between gICA with fixed dimensionality of 20 at different levels of smoothing (4, 6, and 8 mm) against a template obtained with the same dimensionality and with 12 mm smoothing (red, blue, and green for 1.5, 3, and 7 T data, respectively). **(A)** Number of components with a correlation coefficient higher than 0.25 with one of the components of the template. **(B)** Mean correlation across those components with the correlation coefficient higher than 0.25.

For each magnetic field strength, the gICA with dimensionality of 20- and 12-mm smoothing used in Experiment 1 was used as the template for the gICA with the same fixed dimensionality and lower levels of smoothing (4, 6, and 8 mm). Results are shown in Figure 3. The plots report the number of components that are spatially matched (Pearson correlation r > 0.25) by a component in the template, at different levels of smoothing (panel A) and the mean correlation coefficient across matched components (panel B). At 1.5 T, smoothing below 6 mm significantly reduces the number of matched components (from 14 matching components at 8 mm, to 4 at 4 mm), suggesting lower robustness of the results compared to those obtained at higher field strengths. A similar effect is also visible at 3 T, albeit, even at 4 mm smoothing, more than 10 components match the template. On the contrary, at 7 T, the number of matched components is only mildly affected by smoothing. The mean correlation is larger at higher field strength, and its trend with smoothing has a similar slope across fields, indicating that matched components become overall more similar with increased smoothing and field strength.

### Experiment 3

Using the same three templates of Experiment 2 (gICA with dimensionality = 20 and smoothing = 12 mm, one for each magnetic field strength), we evaluated the results of gICA with automatic dimensionality at all smoothing levels. The number of components estimated by MELODIC varied from a minimum of 30 (obtained for 1.5 T and 12 mm smoothing) to a maximum of 145 (for 7 T and 4 mm smoothing). The number of components estimated at all magnetic field strengths and all levels of smoothing, as well as the number of matched components with the respective template, is reported in Table 4.

**Table 4:** Total number of components found with automatic dimensionality and number of matching components with the respective template for each magnetic field strength and smoothing level.

	Matched components/total components			
Field	4 mm	6 mm	8 mm	12 mm
1.5 T	9/52	25/49	27/43	24/30
3 T	26/93	34/77	36/67	46/83
7 T	47/145	45/110	45/93	45/103

**Figure 4:**
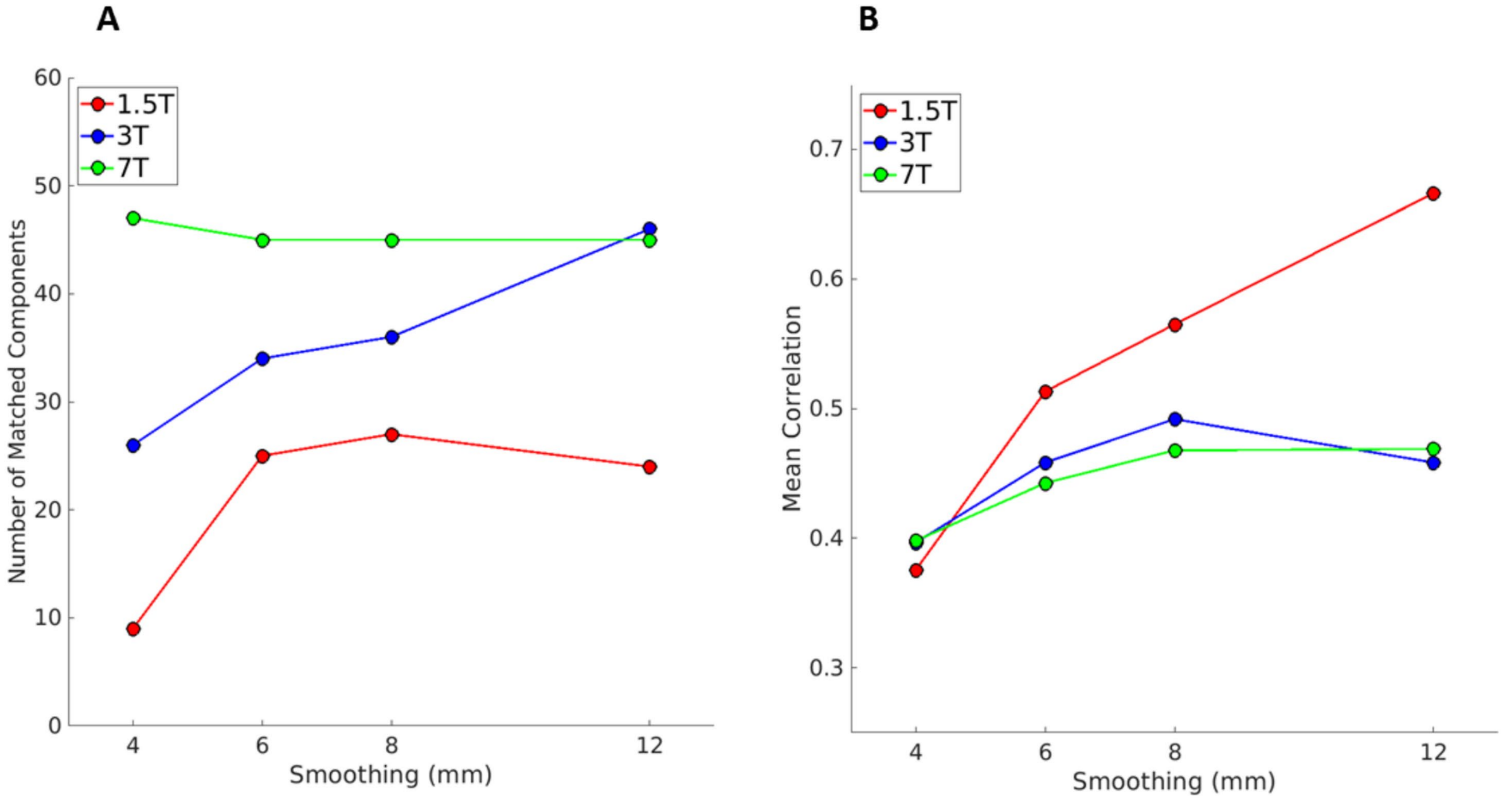
Results of Experiment 3, i.e., for each magnetic field, the comparison between gICA obtained with automatic dimensionality at different levels of smoothing (4, 6, and 8 mm) against the template obtained with a fixed dimensionality of 20- and 12-mm smoothing (red, blue, and green for 1.5, 3, and 7 T data, respectively). Panel **A** shows the number of components with a correlation coefficient higher than 0.25; panel **B** shows the mean correlation coefficient across matched components. The higher apparent mean correlation at 1.5 T is due to the lower number of detected components.

Results of the comparison with the template are shown in Figure 4. Panels A and B show the number of matching components and their mean correlation as a function of smoothing, as in Figure 3. Again, the number of components matching the respective template increases at higher magnetic fields (panel A). The mean correlation in panel B appears higher at 1.5 T. This is because at 1.5 T there are overall less components, and since the acquisition matrix size is smaller than at higher fields, because the voxel size is larger and the FOV is similar, components found at 1.5 T have larger size than those at higher fields. Correlation coefficients are not affected by the components’ extension, but components of larger size are more likely to share similarities with the template at lower smoothing, as they cover the same FOV, and therefore the overall similarity to the template is overestimated when only matching components are considered.

### Experiment 4

**Figure 5:**
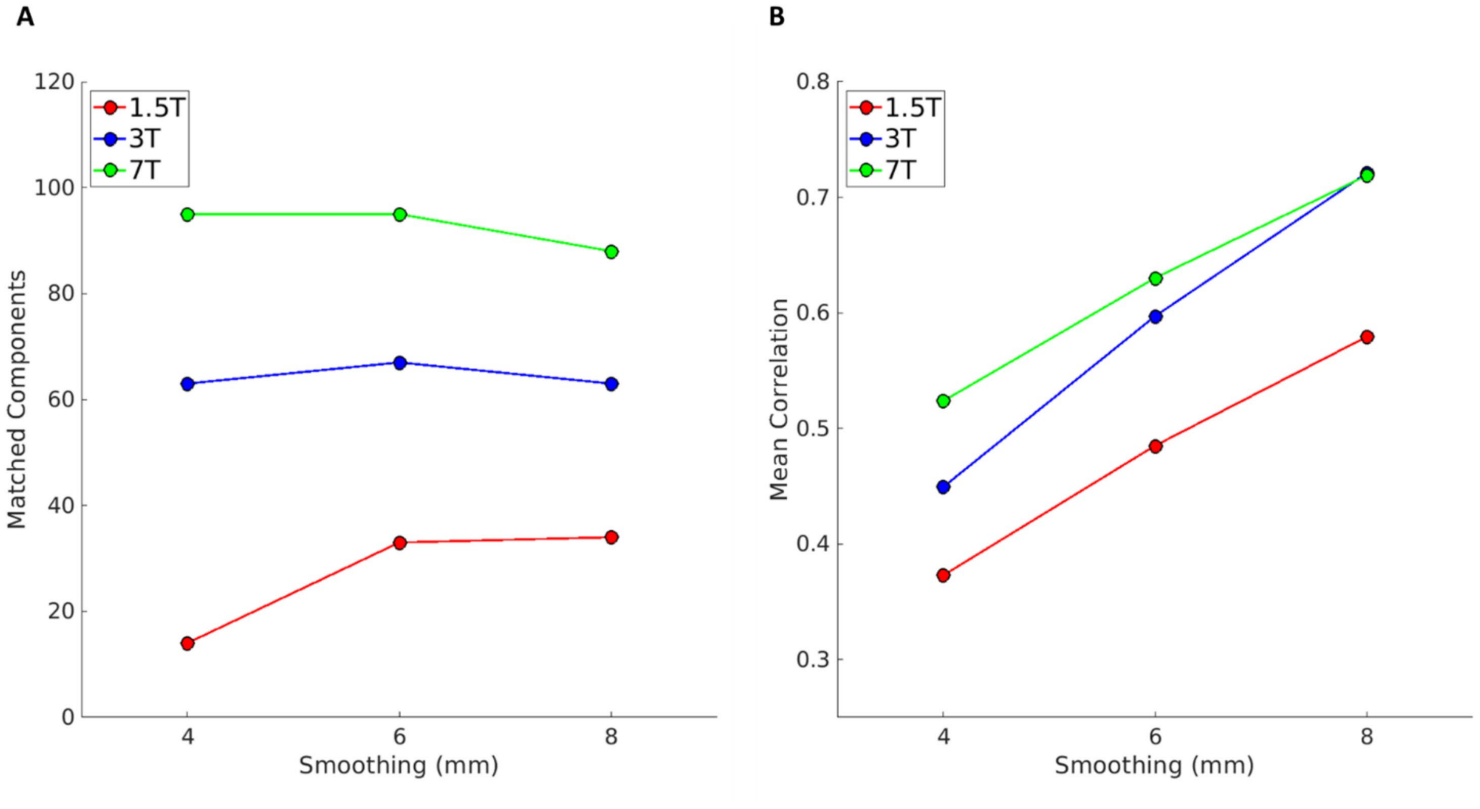
Results of Experiment 4, i.e., for each magnetic field, the comparison between gICA obtained with automatic dimensionality at different levels of smoothing (4, 6, and 8 mm) against the template obtained with automatic dimensionality and 12 mm smoothing (red, blue, and green for 1.5, 3, and 7 T data, respectively). Panels **A** and **B**, respectively, show the number of matched components with a correlation coefficient higher than 0.25 with one of the components of the template and the mean correlation across those components with the correlation coefficient higher than 0.25. More components are matched at higher fields, and at 7 T the number slightly increases with decreased smoothing (panel **A**).

Here, we used the gICAs analysed in Experiment 3 (automatic dimensionality and smoothing = 12 mm, one for each magnetic field strength) as the templates to compare with the gICAs results obtained with automatic dimensionality and different smoothing levels. Results are displayed in Figure 5. With increased field strength, there are more matching components (panel A) with a higher mean correlation coefficient (panel B). Interestingly, at 7 T, the number of matching components increases slightly with lower smoothing, suggesting that components found with 12 mm smoothing are also found at lower smoothing levels. Mean correlation values have a similar behavior to that in Figure 3B, with correlations increasing with a larger level of smoothing and a higher magnetic field.

**Figure 6:**
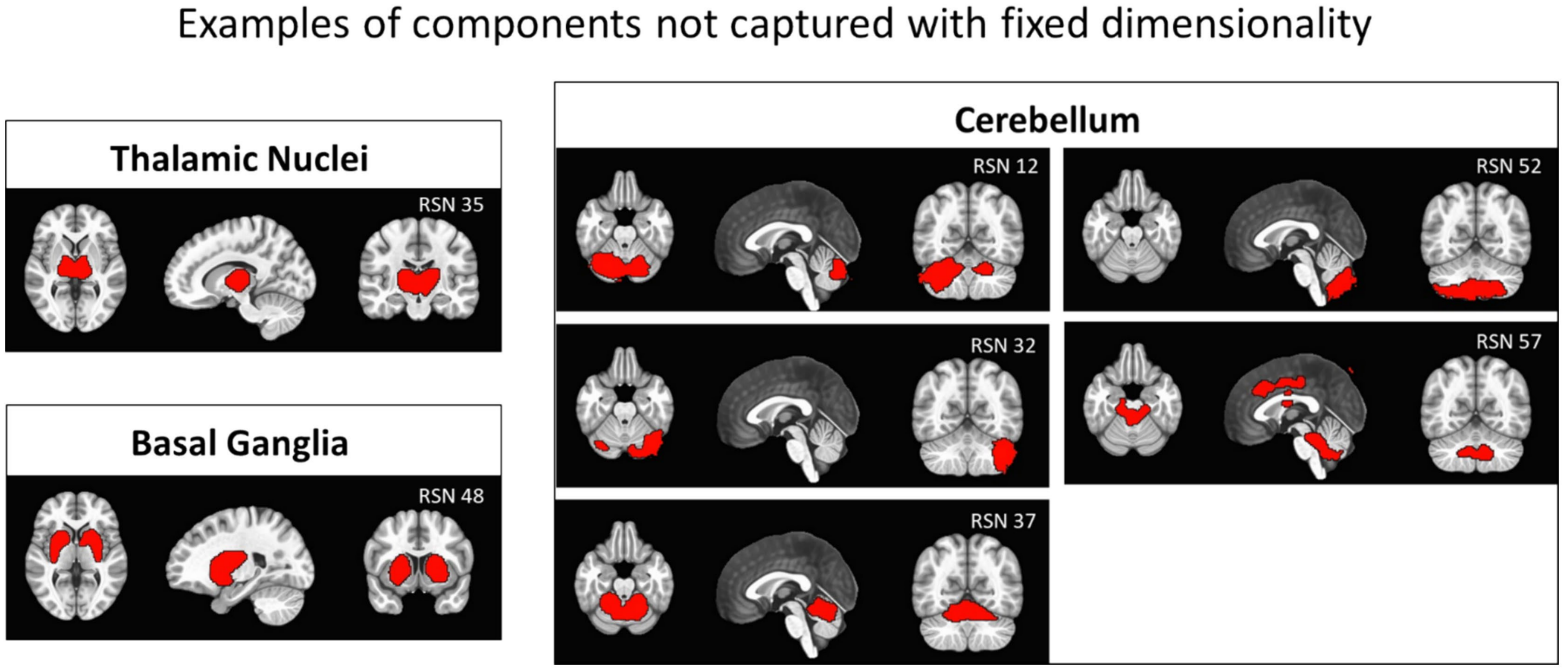
Examples of components found on 7 T data with automatic dimensionality (smoothing = 12 mm) that were not observed with dimensionality constrained to 20: left panels show the thalamic nuclei (top, RSN 35) and the basal ganglia (bottom, RSN 48). Right panels show five components covering the cerebellar region. Compare with the third column in Figure 2, where no thalamic or basal ganglia components are present, and the cerebellum is covered only partially in RSN 4, row 5.

Figure 6 shows examples of components obtained at 7 T with 12 mm smoothing and automatic dimensionality that are not captured when dimensionality is fixed to 20: in particular, the RSN 35 and RSN 48 components identify the thalamic nuclei and the basal ganglia, and even five non-overlapping cerebellar components cover the cerebellum. With dimensionality fixed to 20, we only found a partial coverage in RSN 4, shown in Figure 2 in row 5.

## Discussion

To our knowledge, this is the first work that compares ICA on rs-fMRI data at three different field strengths with the aim of exploring the dependency of ICA on magnetic field strength, model order, and spatial smoothing, which was used as a proxy for different levels of spatial resolution. Our results show that, both when the number of components is fixed or estimated automatically, and therefore free to vary, ICA results are more consistent at higher field strengths, both across smoothing levels and when compared to an external reference. The latter is suggested by the results of Experiment 1, where the RSNs obtained at different fields were compared to the template derived by Smith et al. (2009). Despite this template cannot be regarded as a biological ground-truth, it can be used to estimate deviations from a reference obtained with a significant number of subjects. Our data show that deviations from this template are more severe at lower field, with higher stability of RSNs at higher field. Further studies are needed to identify a consistent and robust method for selecting this number. While this work suggests that the expected number of components should be adapted to the field strength and spatial resolution at which data are acquired, further studies are needed to identify a consistent and robust method for selecting this number. Moreover, this study suggests that the expected number of components should be adapted to the field strength and spatial resolution at which data are acquired. This is in line with previous studies (Smith et al., 2009; Li et al., 2007; Abou-Elseoud et al., 2010) showing that biologically relevant networks can be found across a wide range of dimensionalities, possibly reflecting the biological complexity of the signals and systems (Beckmann, 2012). In Kreitz et al. (2023), the authors compared 3 T and 7 T acquisitions and reported findings similar to ours regarding the increased spatial sensitivity of ICA at higher field strengths, but their analysis was limited to 20 components. Additionally, different algorithms can find different ICA on the same dataset (Wei et al., 2022). Moreover, from the perspective of using the ICA approach to study the functional brain organization across development and aging, as well as in various neurological and psychiatric conditions, we have to consider that age differences (Littow et al., 2010) and pathologies (Calhoun, 2009; Du et al., 2015; Hyatt et al., 2015; Öngür et al., 2010; Sorg et al., 2007; Syed et al., 2017; Xiong et al., 2020) can also affect the number of components that should be expected. Therefore, fixing its number might result in overlooking differences across groups or individuals.

The number of components is usually kept low to facilitate visualization and data handling, but our results indicate that constraining the number of components can lead to missing RSNs, as shown in Figure 6. Classifying components by visual inspection, especially when their number is high, can be difficult and time-consuming. A possible strategy that is frequently used is to compare components to an external template through spatial correlation. This allows the identification of the most relevant networks, as we did in Experiment 1, but has limitations (Zuo et al., 2010). Using an external template with a fixed number of components, such as Smith et al. (2009) or Laird et al. (2011), can limit the exploration of the brain functional architecture by missing extra components that may merge with other networks. Additionally, to our knowledge, templates for 7 T rs-fMRI data are not available, which can undermine the added value of increased field strength. For example, the thalamic nuclei in the template RSN from Smith et al. (2009) are included in the occipital visual network (Figure 2), while in our data at 7 T with automatic dimensionality, the thalamic nuclei appear as a separate component (Figure 6). Analogously, the basal ganglia in the template are partially included in the executive control network, but are present as an individual component in our results at 7 T. Moreover, the cerebellar component appears in the template from Smith et al. (2009) as a single component, while in our 7 T data, the cerebellum is not covered when dimensionality is fixed to 20, and it appears as 5 separate components when dimensionality is automatically determined. In general, the biological relevance of RSNs can be based on morphological or functional prior knowledge, as in the case of the aforementioned subcortical components. However, the validity of more complex networks that may be revealed by not fixing a-priori the number of expected components could be verified if they are consistently identified by independent studies. In this case, their functional relevance can be addressed at later stages of investigation, not very differently from what has been done with the DMN, which was first identified in independent studies and only later given a functional significance. On the other hand, reaching a consensus on the plausible number of expected components could ease the harmonization across studies and reduce the computational demands.

It should be noted that we did not discriminate between true activation components and noise components. Noise components do not have any functional relevance, but they can be used as nuisance regressors during pre-processing (Pruim et al., 2015; Thomas et al., 2002). Therefore, the robustness of noise components is also of interest, and consistent splitting of artefactual components can improve data cleaning. Additionally, splitting of true activation components has been reported previously (Hyatt et al., 2015; Hu et al., 2016; Zuo et al., 2010; Biswal et al., 2010; Laird et al., 2009; Uddin et al., 2009). Distinguishing between true activation components and noise components can be challenging, especially when their number is high. In general, many different features need to be addressed to separate true networks and artefactual components, the most important being the spatial map, the time course of the network and their power spectrum. Spatial patterns of true components can be expected to be composed of localized clusters of activity, while noise components might appear more randomly distributed; their time course should reflect BOLD-like signals, therefore exhibiting smooth fluctuations; the frequency power spectrum should be dominated by frequencies below 0.1 Hz. Common artefactual components arise from cardiac or head motions, signals from veins or non-grey matter tissue, susceptibility artefacts or acquisition related issues (Salimi-Khorshidi et al., 2014).

Because no single sequence parameter is optimal for all field strengths, we opted to acquire sequences optimized for each field strength to obtain datasets more representative of each condition (Triantafyllou et al., 2005). Nevertheless, the main limitations of this study include, together with the use of non-uniform rs-fMRI acquisitions, the limited number of participants, and the use of spatial correlation as a metric for ICA similarity. The factor most likely to have affected the results is the different TR at 1.5 T (3 s), mandated by scanner capabilities, compared to 3 T and 7 T (2 s), given the fixed acquisition duration of 6 min. This difference might have affected the number of components estimated automatically, since fewer volumes were acquired within the same time period, and it might have made the analysis less sensitive to components characterized by oscillations in a different frequency range (Gong and Zuo, 2025). However, the difference in the number of components between 3 T and 7 T, where the TR was the same, appears consistent with the difference found for the 1.5 T data. Additionally, no comparison was made between ICA components obtained at different field strengths, so the different decrease in the number of matching components with reduced spatial smoothing is not likely to be caused by the different TR, which was shown to affect only slightly ICA on rs-fMRI (Huotari et al., 2019). The limited number of participants in this study may limit the generalizability of our results, but sample sizes comparable to ours are common in fMRI studies. Further studies with more participants are needed to confirm our results. The comparison with external templates and the robustness of our analysis across smoothing levels is an indication that gICA can provide meaningful results even in a small cohort of participants. The use of Pearson’s spatial correlation was dictated by its simplicity in interpretation, but it can over- or underestimate the similarity between functional maps, especially when comparing maps with a large number of voxels (Zuo et al., 2010). However, in our comparisons, any potential underestimation or overestimation should be comparable and approximately uniform across fields and smoothing. While more elaborate approaches of spatial pattern comparisons might improve the accuracy of network matching, spatial correlation is frequently used for comparison across independent components (Smith et al., 2009; Laird et al., 2011). Finally, we assumed that varying spatial smoothing could be treated as an approximation of different spatial resolutions. Although it is not equivalent to acquiring data at different spatial resolutions, and it has been shown to be actually more beneficial than increasing voxel size, especially at high field (Triantafyllou et al., 2006), it constrains the spatial independence of the data (Hu et al., 2016; White et al., 2001), possibly merging activation clusters into one. The benefit of this approach is that it does not introduce confounding factors arising from repeated acquisitions, such as individual differences across sessions or subjects’ motion, which can be particularly detrimental in rs-fMRI, where signal fluctuations can be small (Maknojia et al., 2019).

## Conclusion

ICA is frequently used to generate RSNs, but the number of components is often limited to facilitate data visualization and interpretation. We have shown that this restriction can provide incomplete representations of the resting brain. The expected number of components should instead be based on the level of spatial detail or estimated from the data under consideration. Additionally, our results highlight the added value of ultra-high field in rs-fMRI, as results are less degraded by minimal smoothing at 7 T than at lower field strengths, even when tSNR is matched across fields. This work contributes to rs-fMRI research by clarifying how magnetic field strength, spatial smoothing, and dimensionality affect ICA outcomes, thereby guiding future methodological choices.

## Data Availability

Data may be provided to interested researchers upon request to the corresponding author, after clearance from the IRB.
