# Supplementary material for: Robustness, spatial detail, and pitfalls of fixed ICA dimensionality in resting-state fMRI networks at 1.5, 3, and 7 T
Source: Front Neurosci. 2026 Jan 6;19:1731143. doi: 10.3389/fnins.2025.1731143 (PMC12816346; doi:10.3389/fnins.2025.1731143)
Supplement: Supplementary file 1 [file Data_Sheet_1.PDF]

Supplementary Figures

In the following, we report all the components found with dimensionality fixed to 20, at all smoothing levels and magnetic field strength. All figures show the Z-statistics as output by MELODIC on 20 axial slices superimposed to the MNI template. Figures were obtained by thresholding probability maps at 0.95, with a minimum cluster size of 80 voxels.

Group ICA at 1.5T with 12mm smoothing and Dimensionality 20

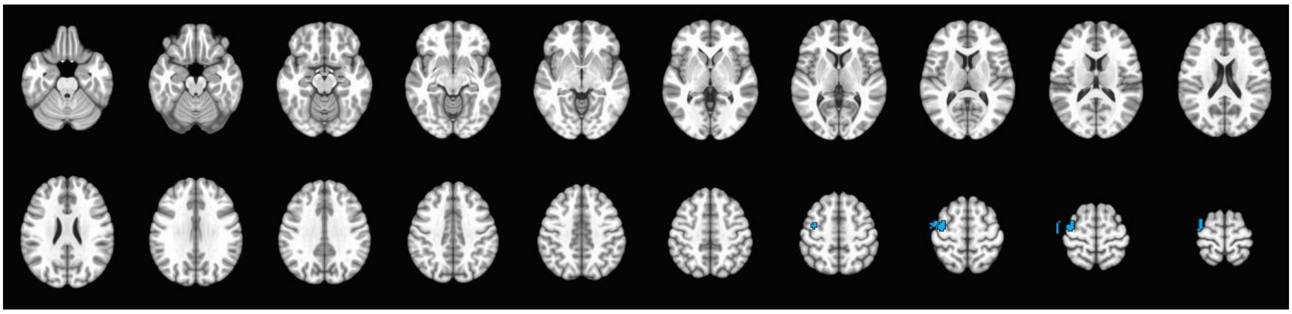

Figure S1.1

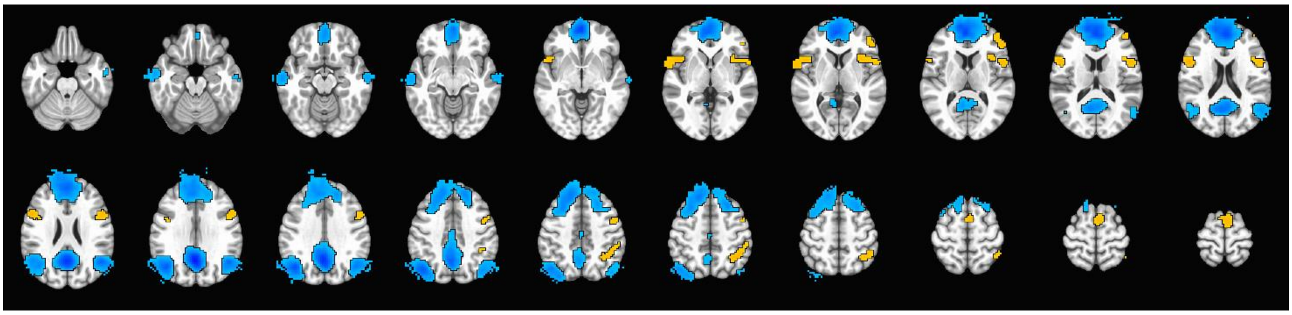

Figure S1.2

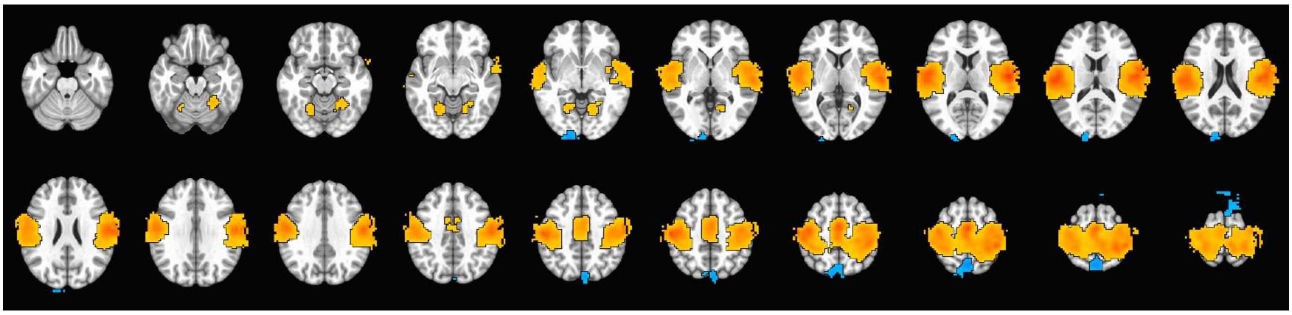

Figure S1.3

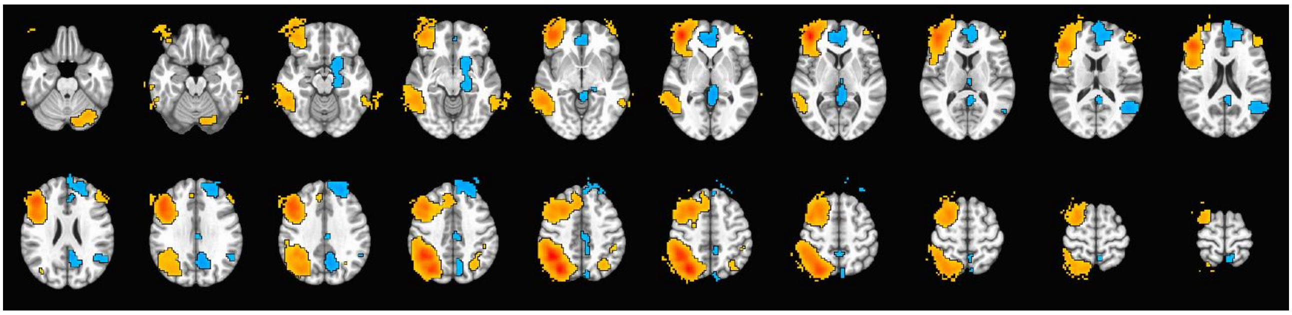

Figure S1.4

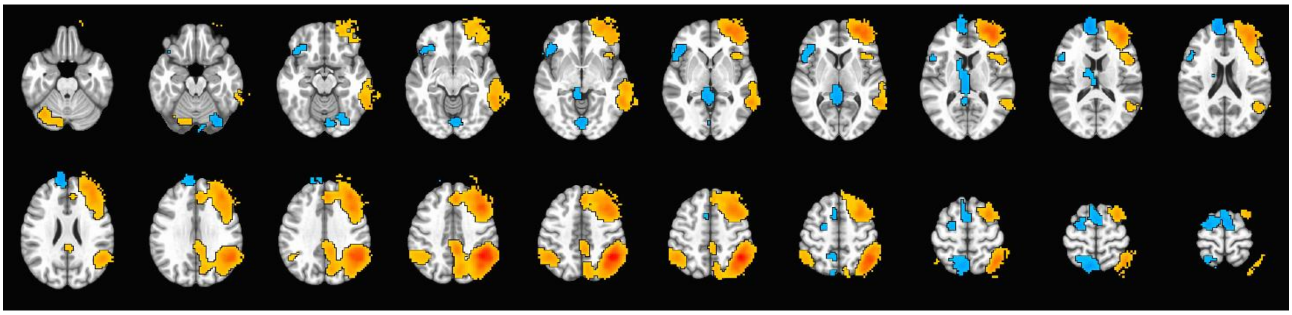

Figure S1.5

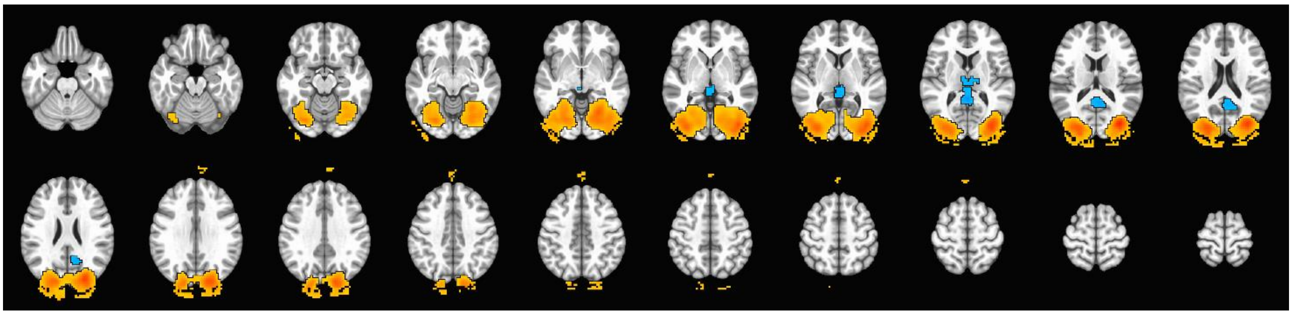

Figure S1.6

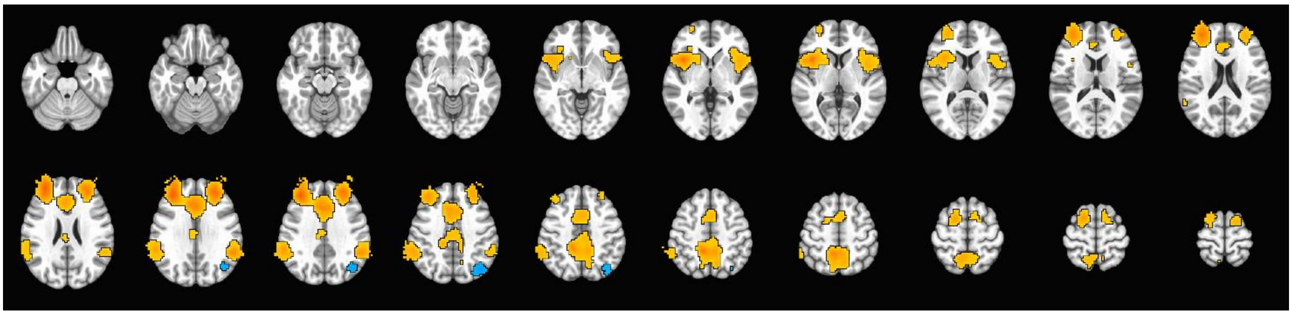

Figure S1.7

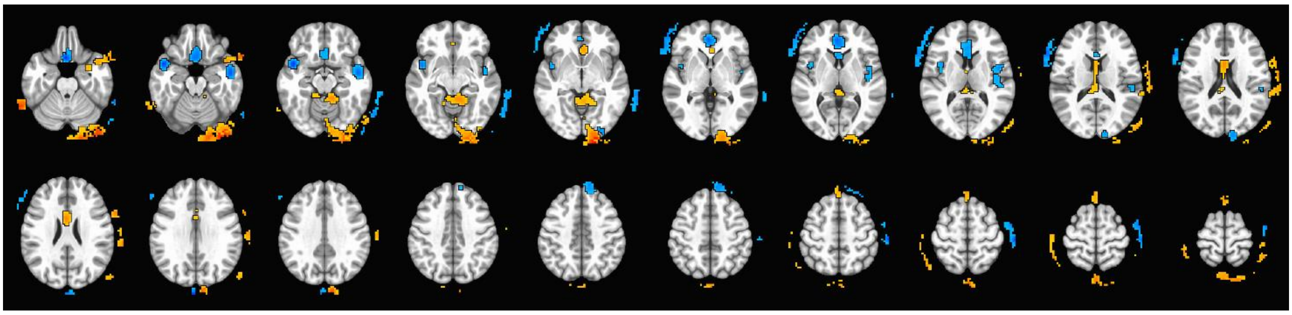

Figure S1.8

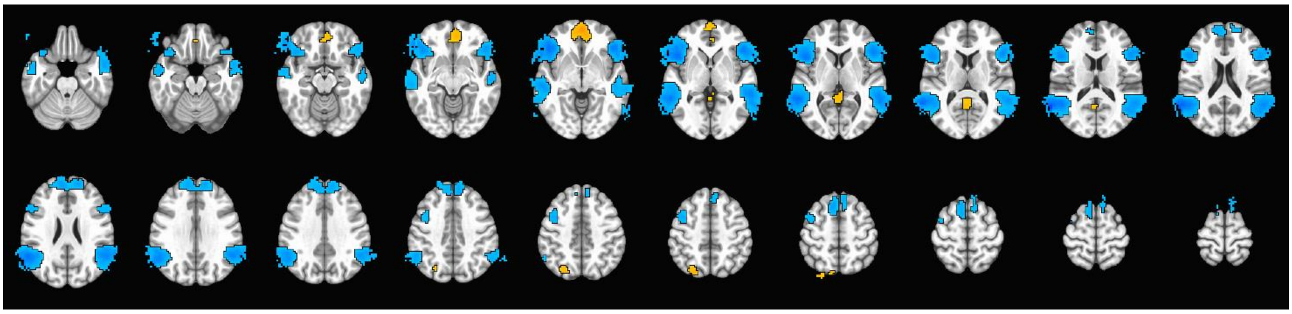

Figure S1.9

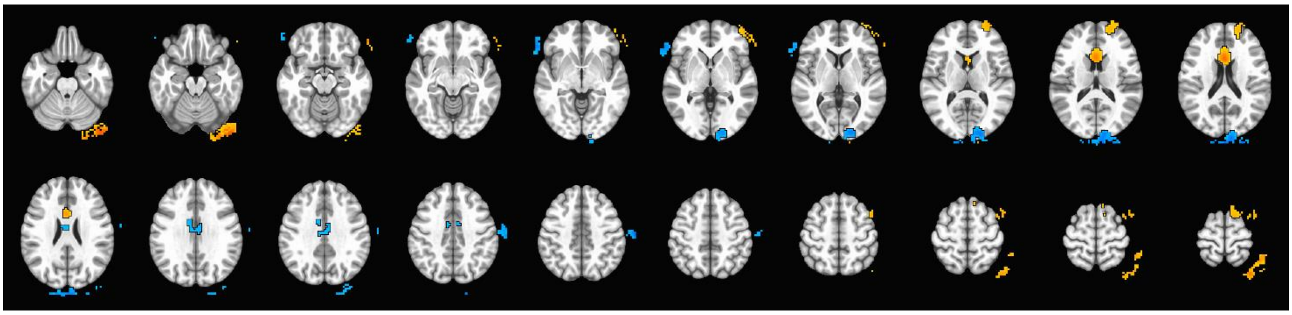

Figure S1.10

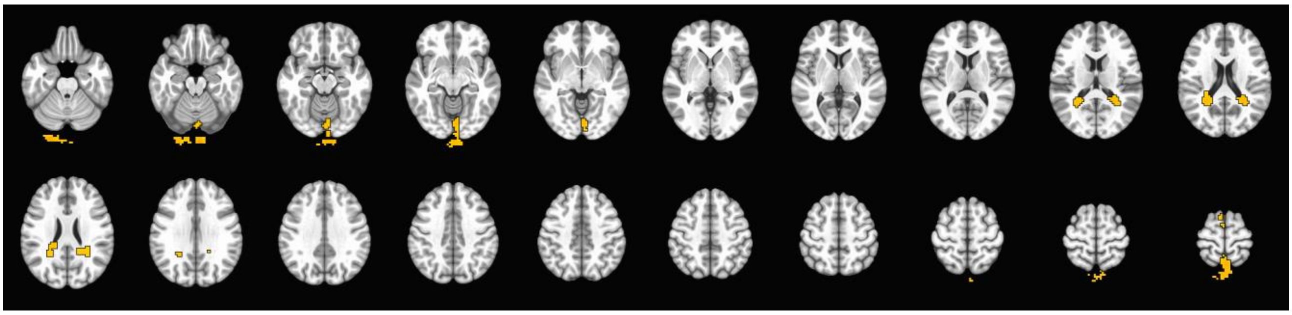

Figure S1.11

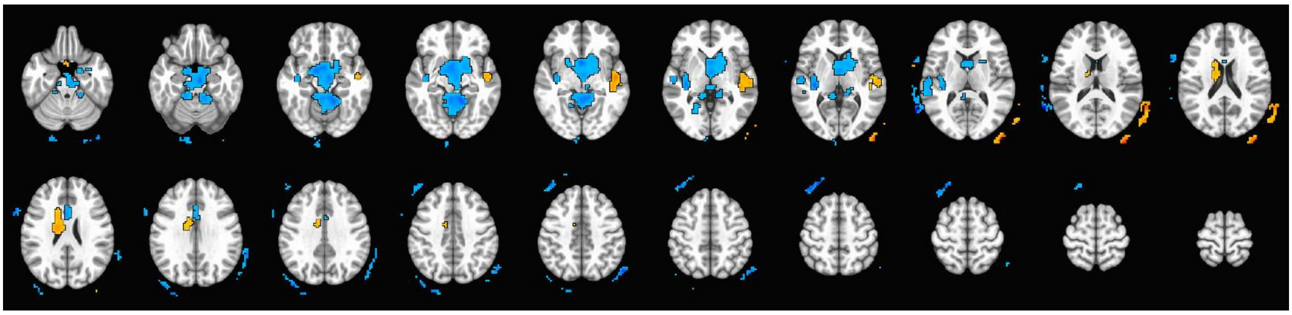

Figure S1.12

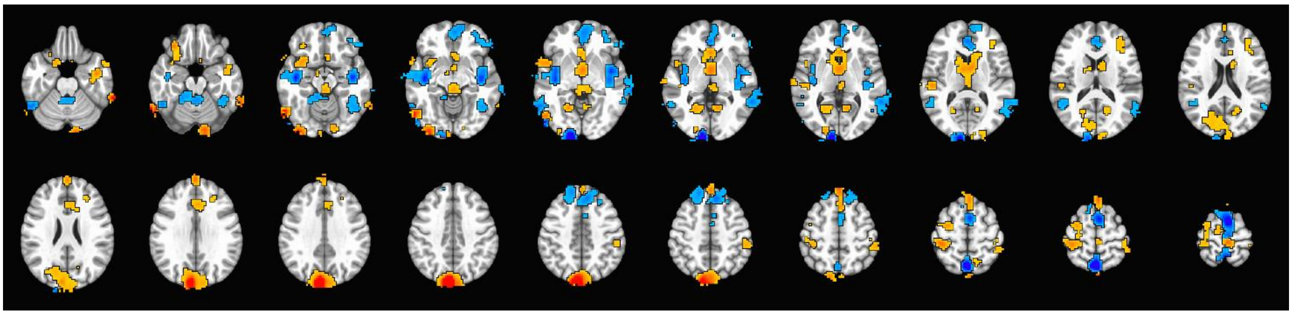

Figure S1.13

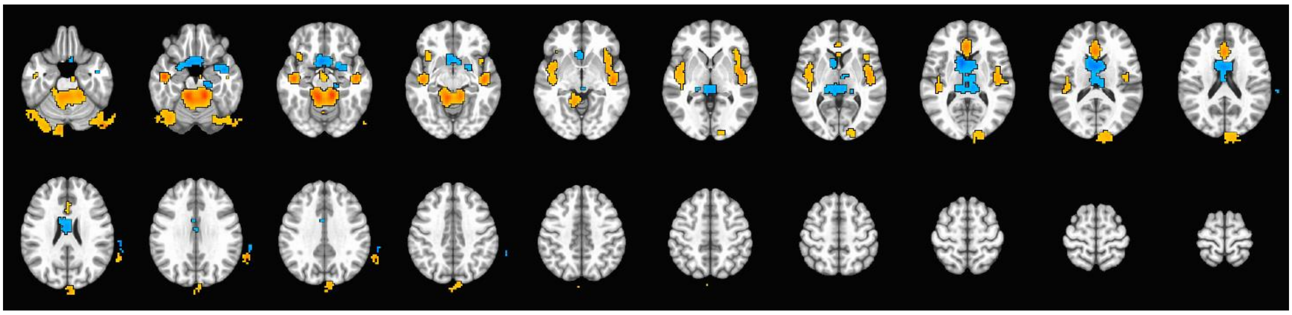

Figure S1.14

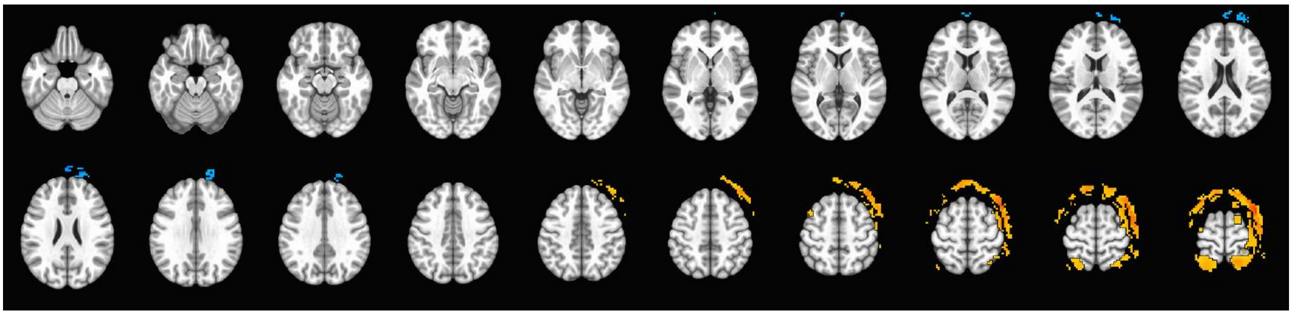

Figure S1.15

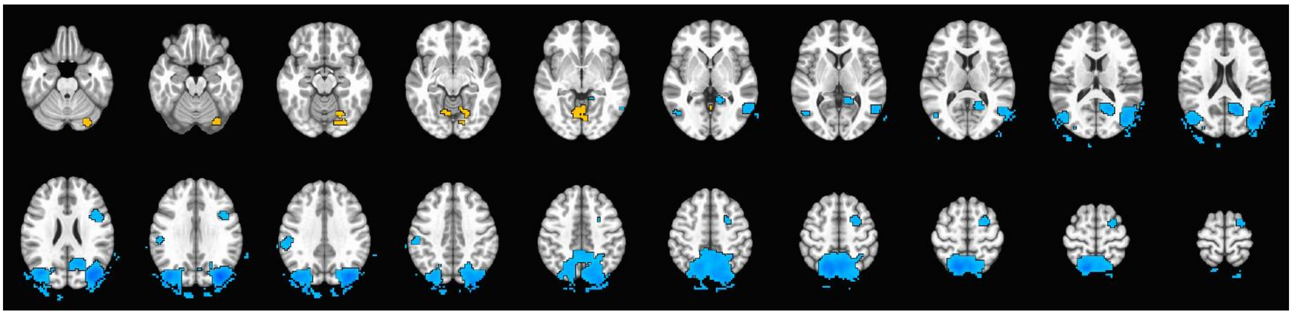

Figure S1.16

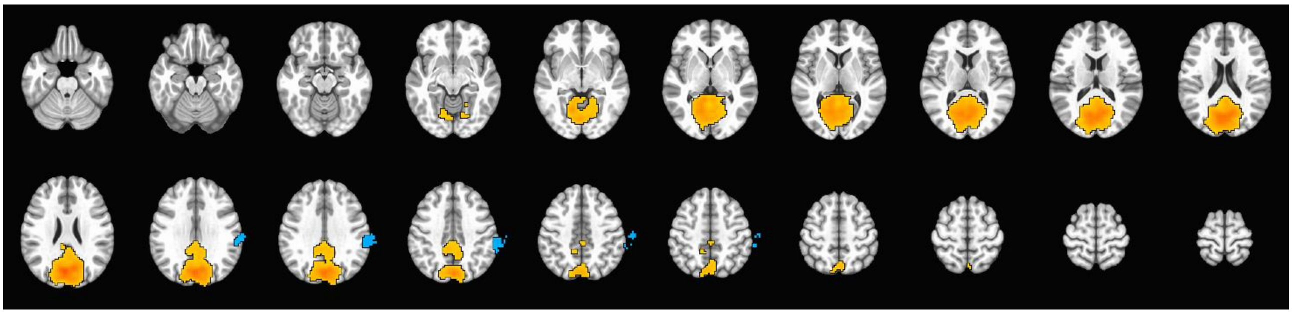

Figure S1.17

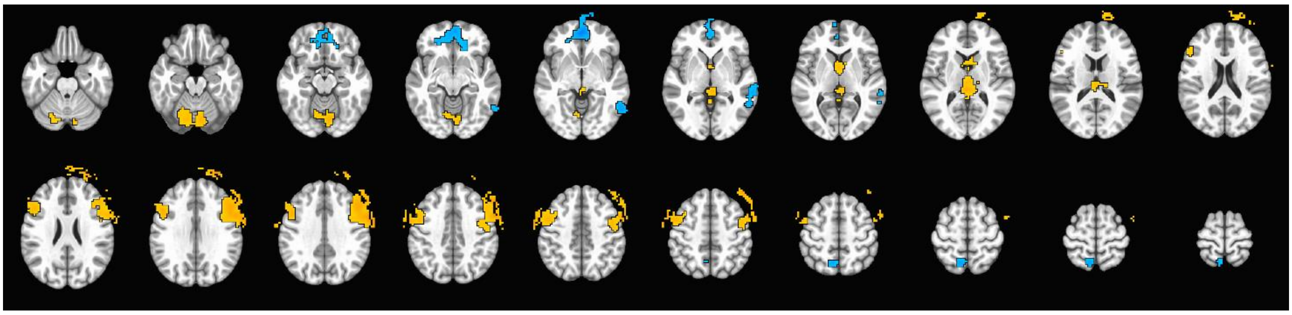

Figure S1.18

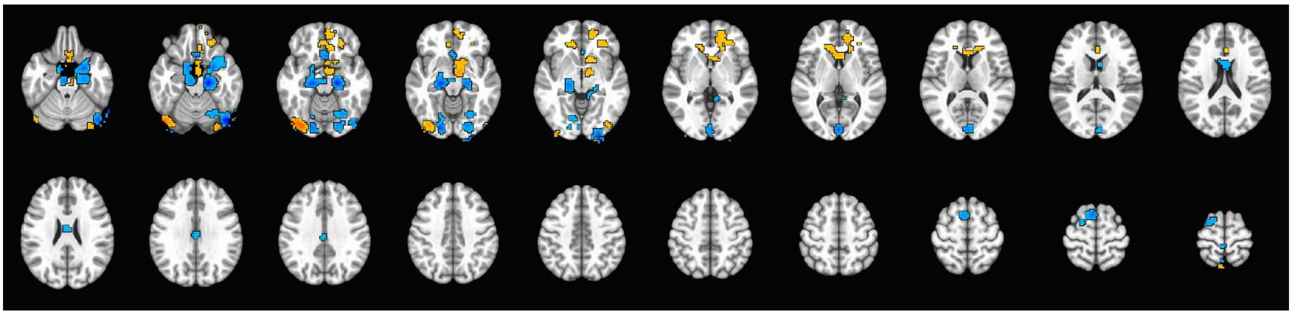

Figure S1.19

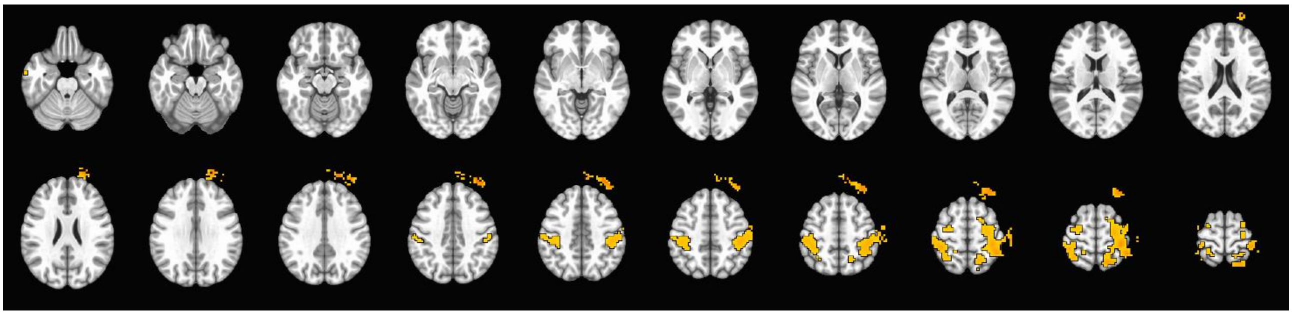

Figure S1.20

Group ICA at 1.5T with 8mm smoothing and Dimensionality 20

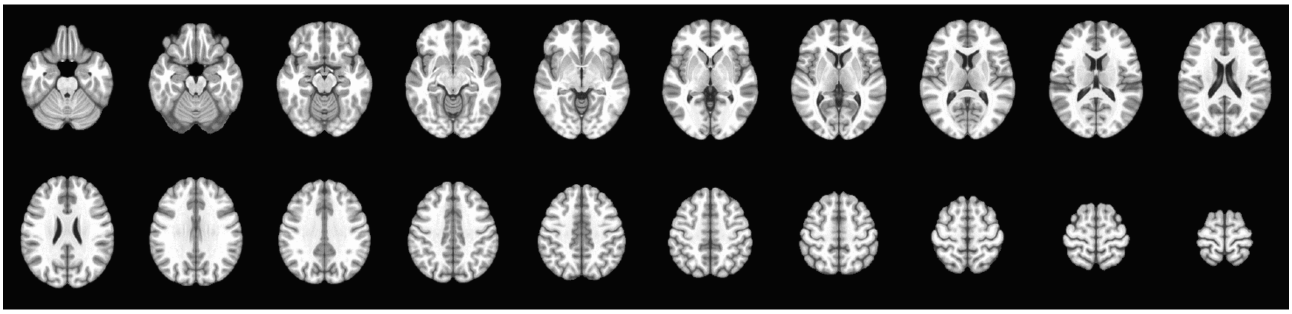

Figure S2.1

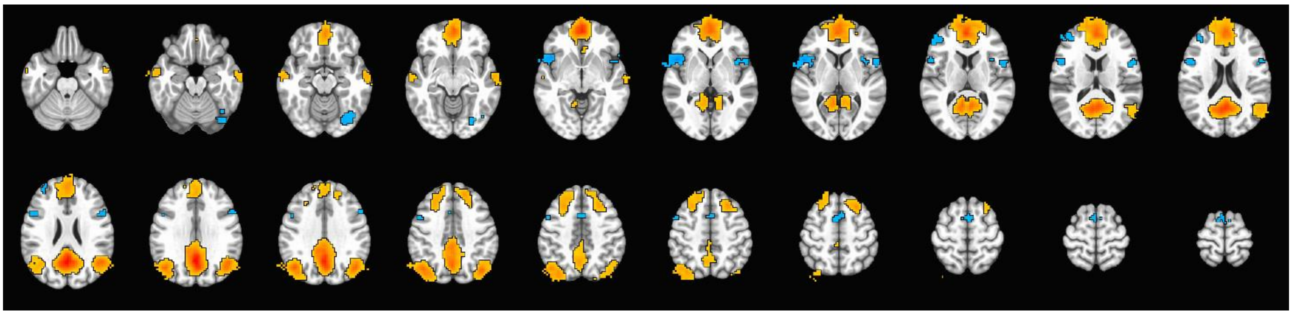

Figure S2.21

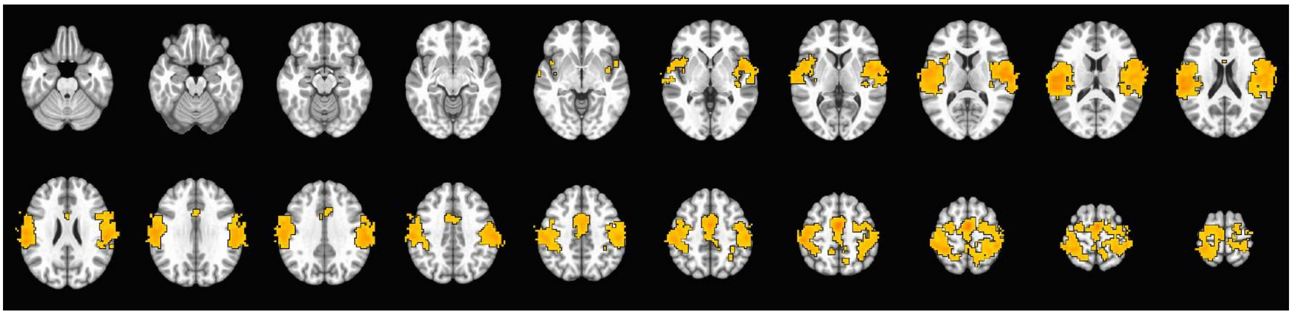

Figure S2.22

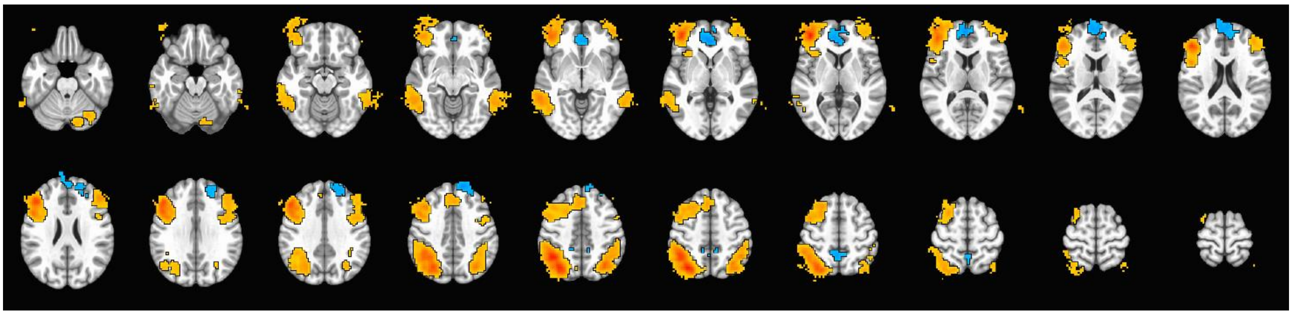

Figure S2.23

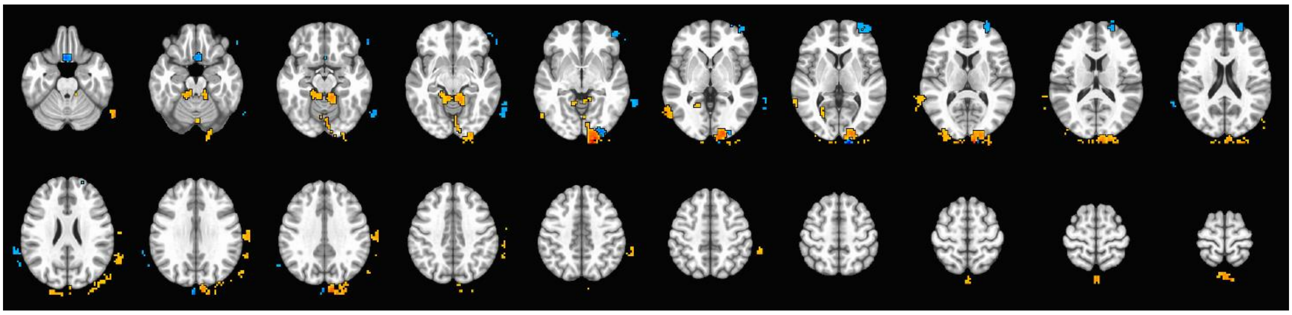

Figure S2.24

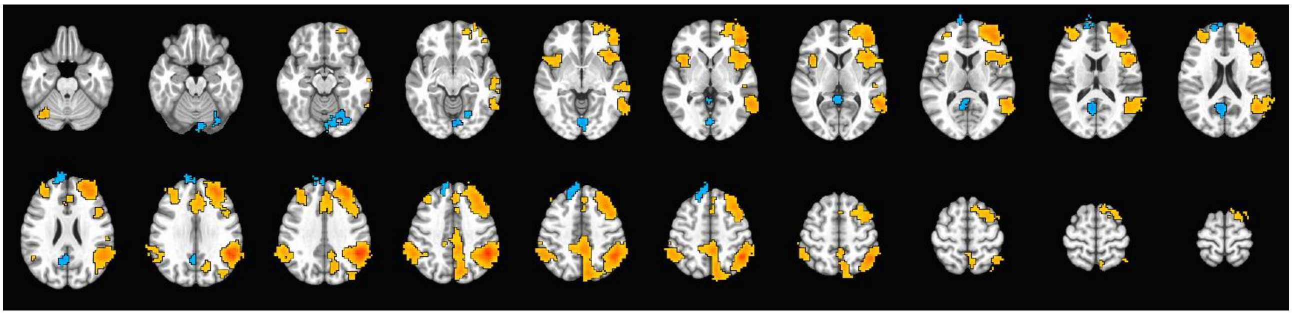

Figure S2.25

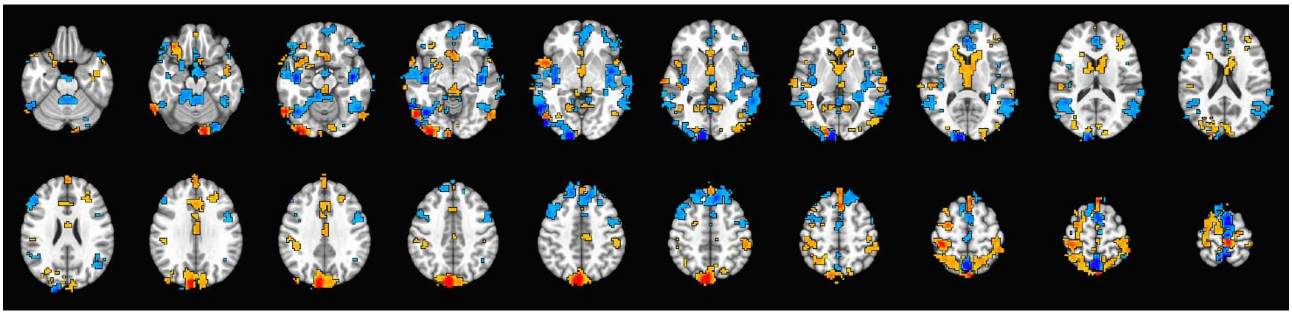

Figure S2.26

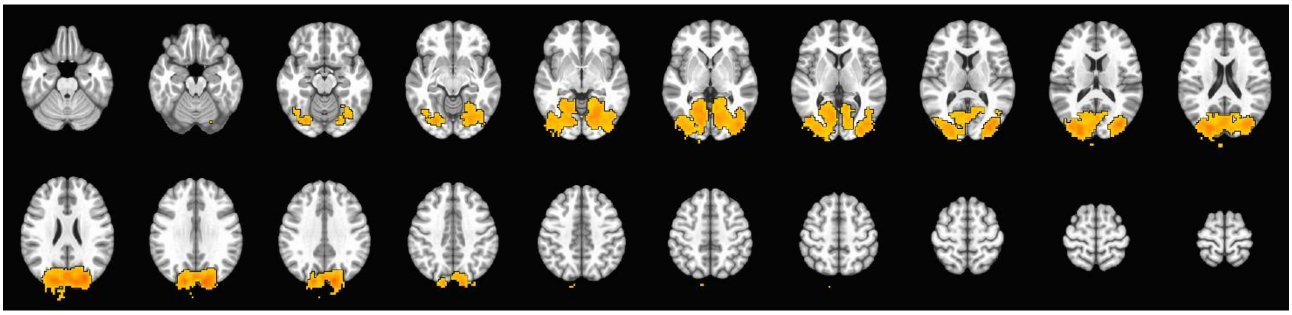

Figure S2.27

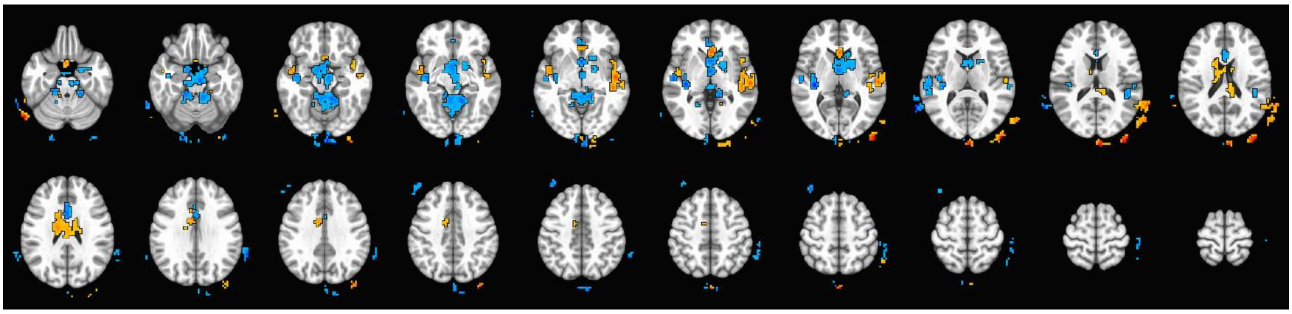

Figure S2.28

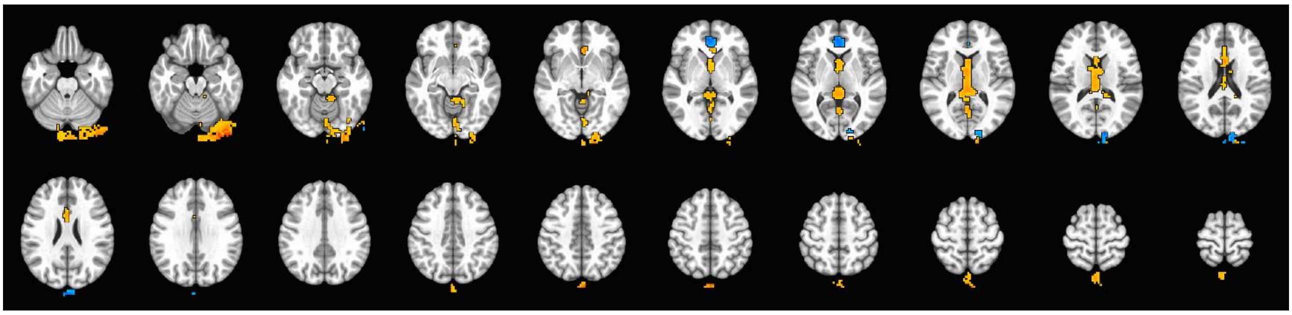

Figure S2.29

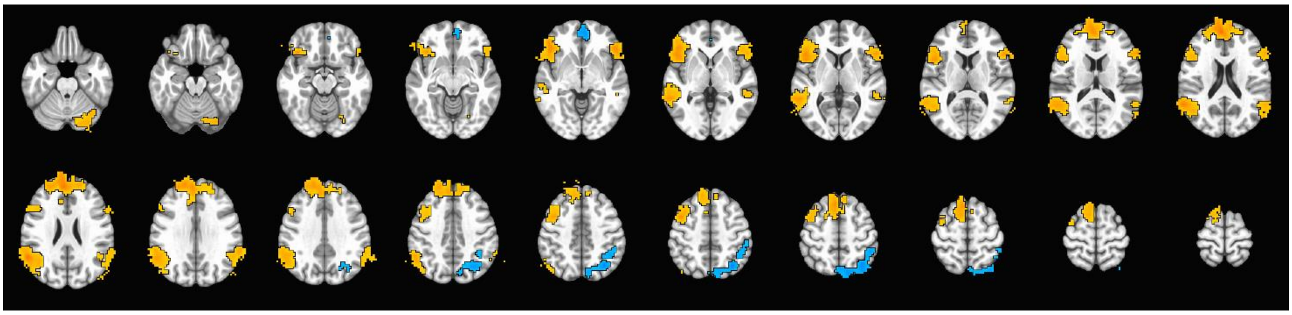

Figure S2.30

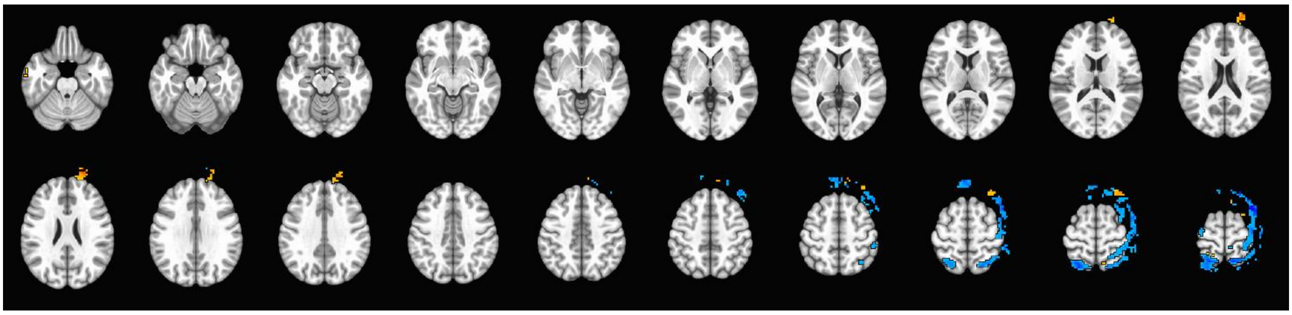

Figure S2.31

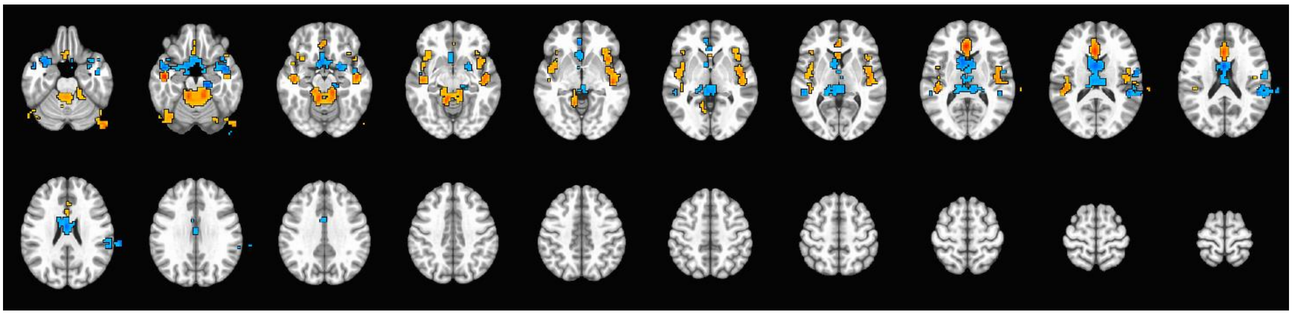

Figure S2.32

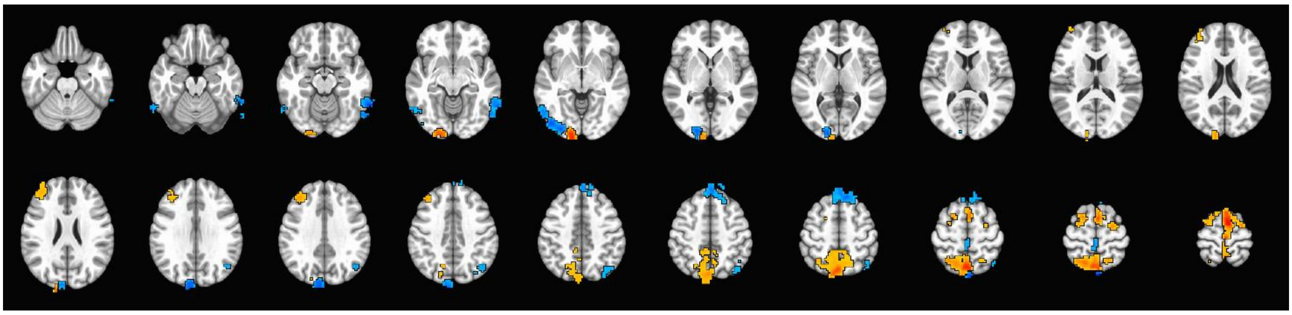

Figure S2.33

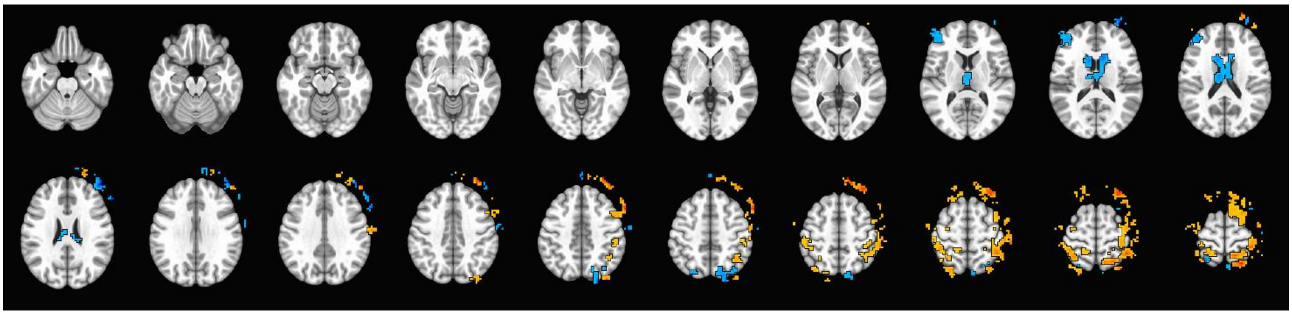

Figure S2.34

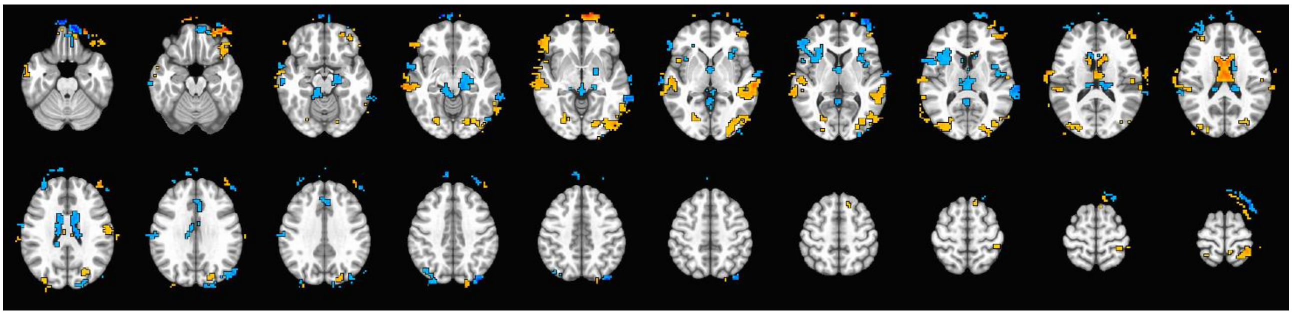

Figure S2.35

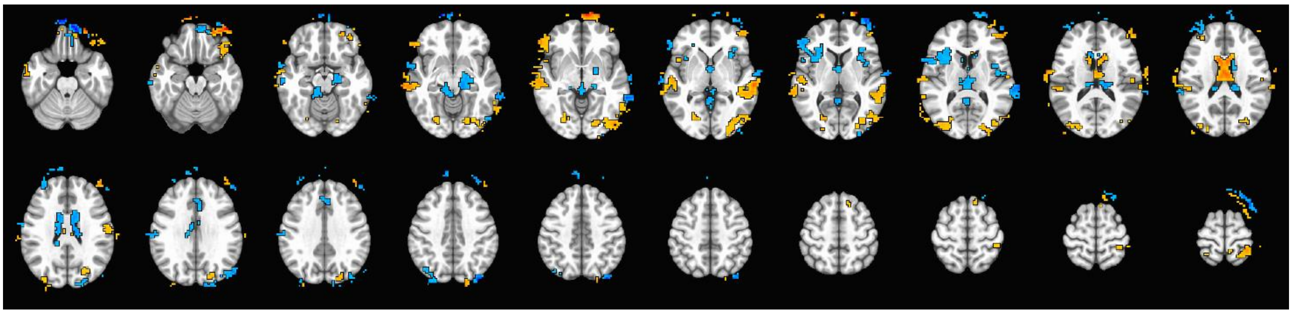

Figure S2.36

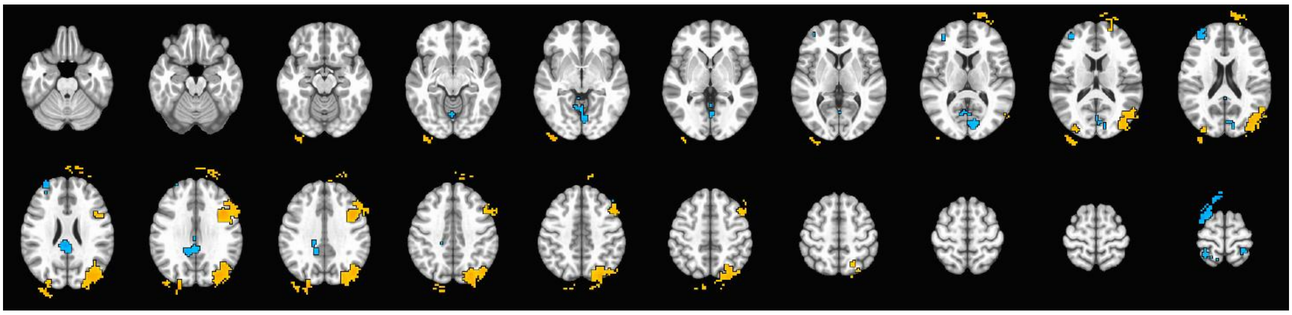

Figure S2.37

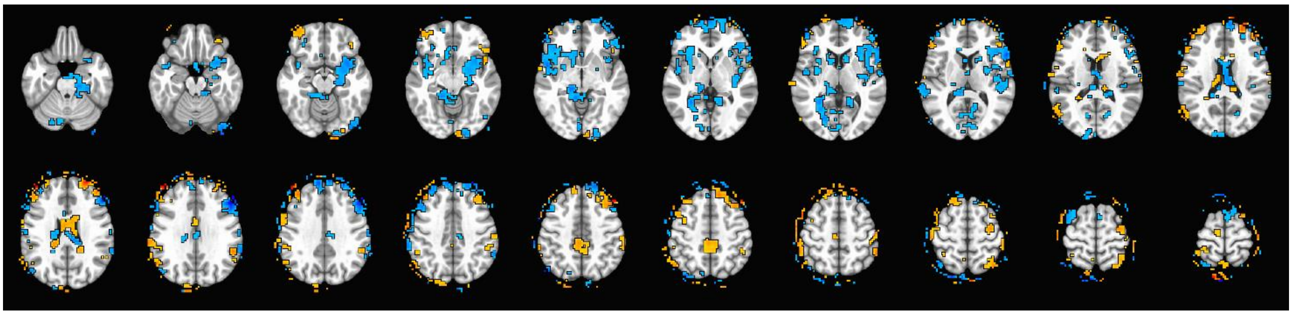

Figure S2.38

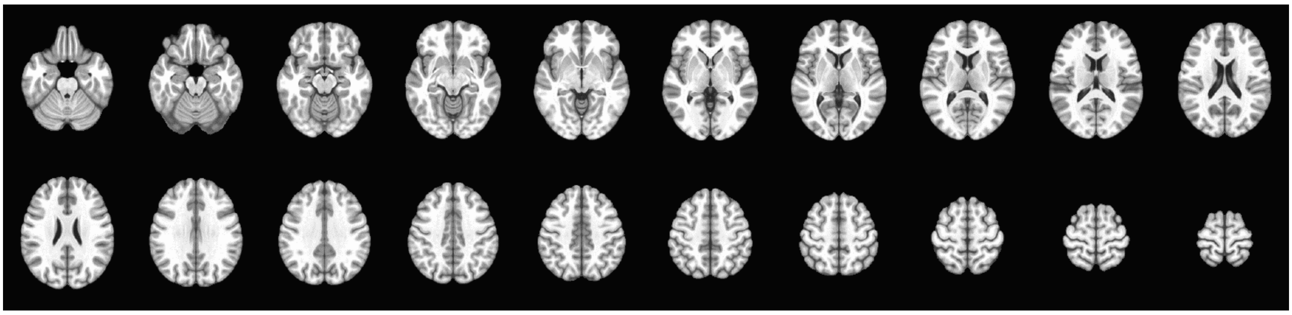

Figure S2.39

Group ICA at 1.5T with 6mm smoothing and Dimensionality 20

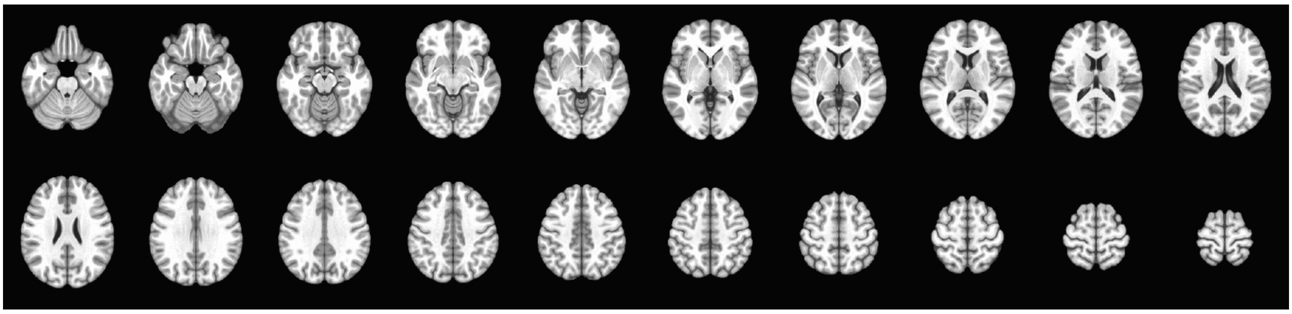

Figure S3.1

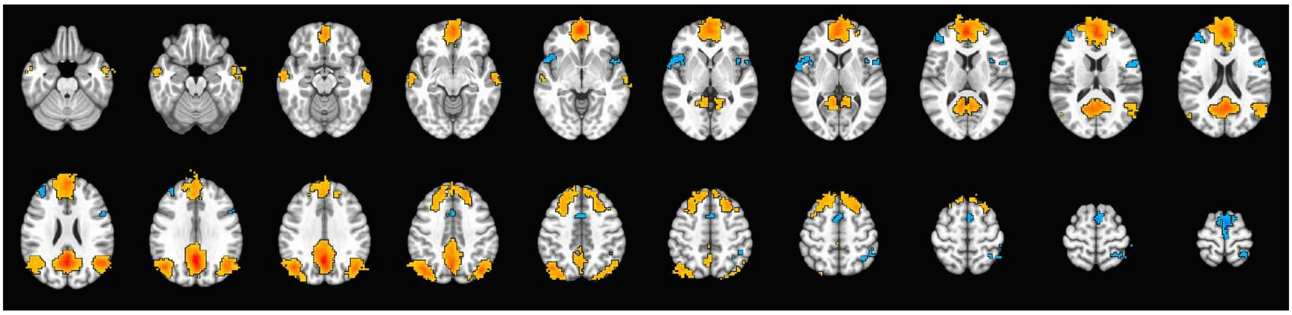

Figure S3.40

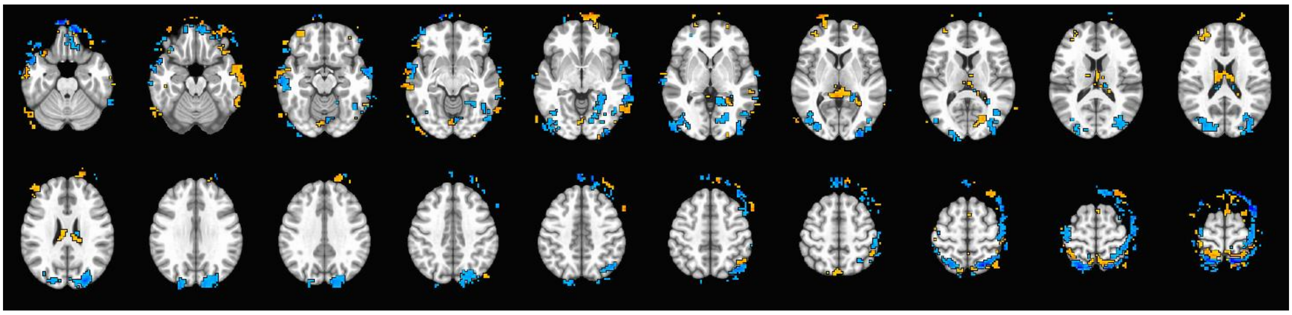

Figure S3.41

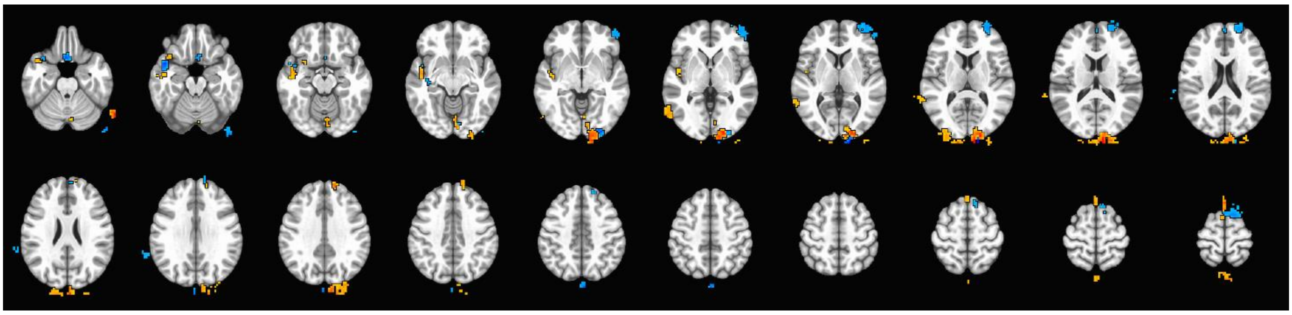

Figure S3.42

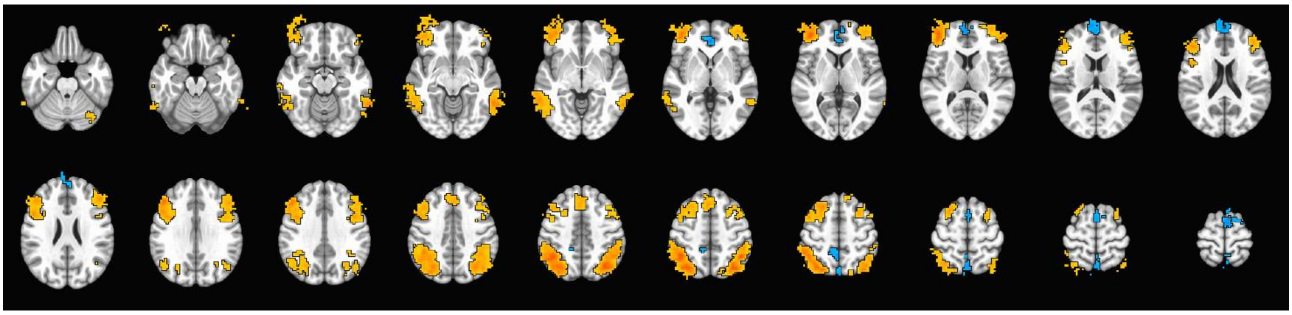

Figure S3.43

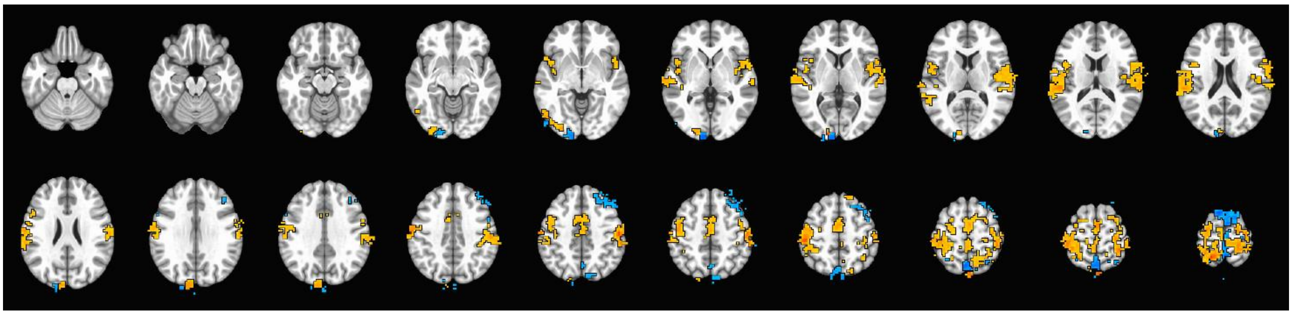

Figure S3.44

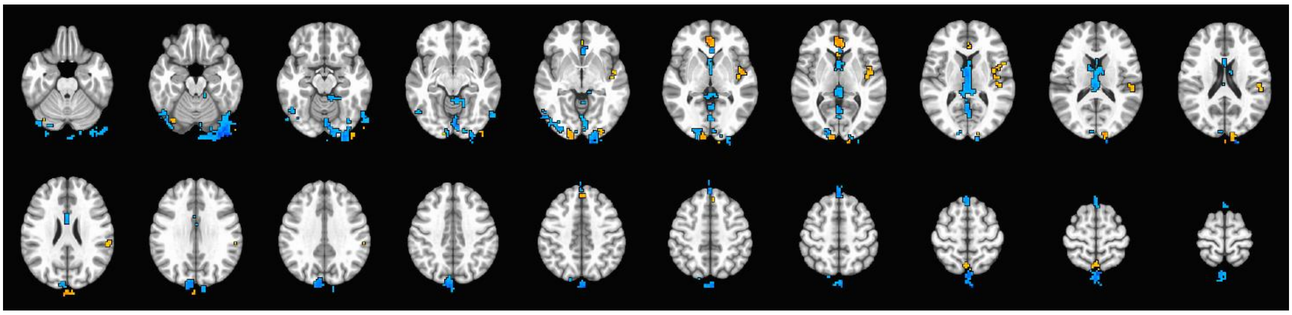

Figure S3.45

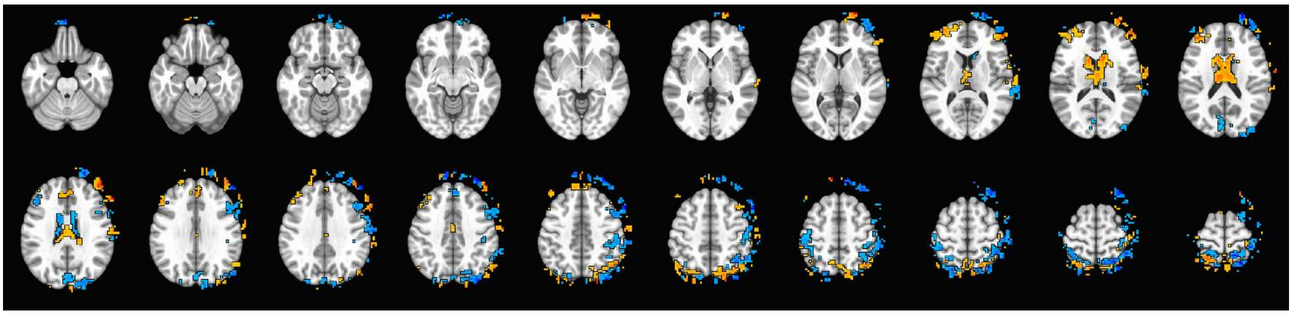

Figure S3.46

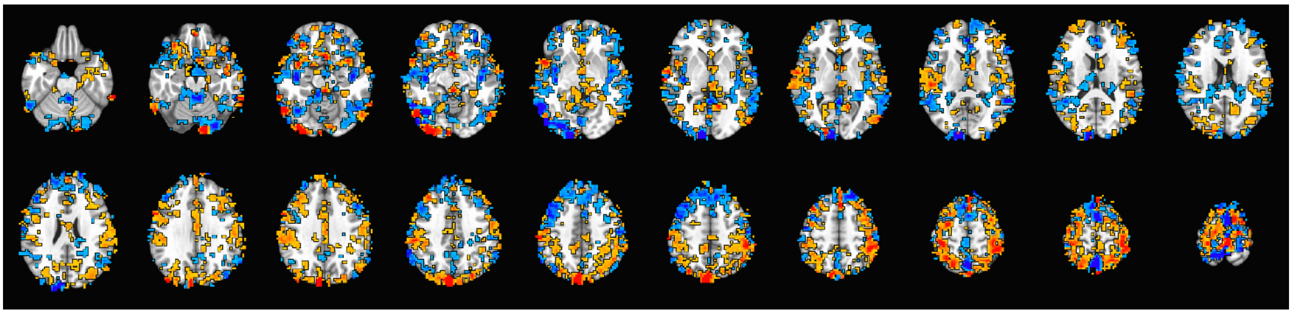

Figure S3.47

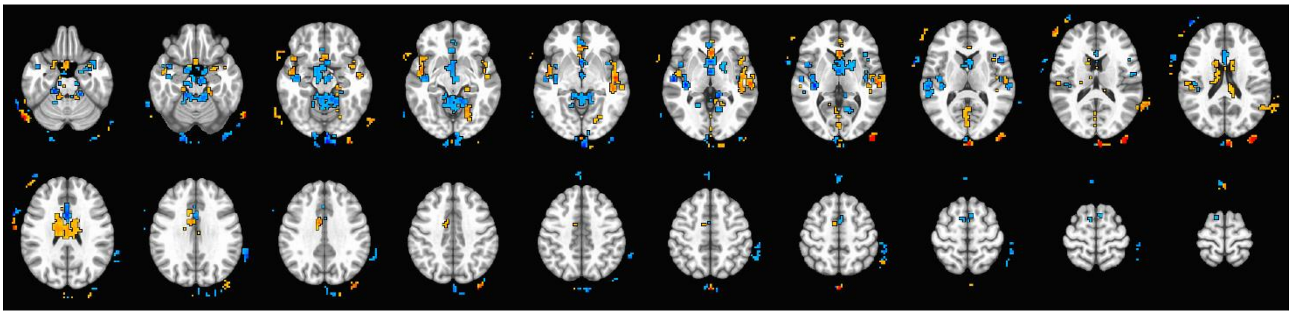

Figure S3.48

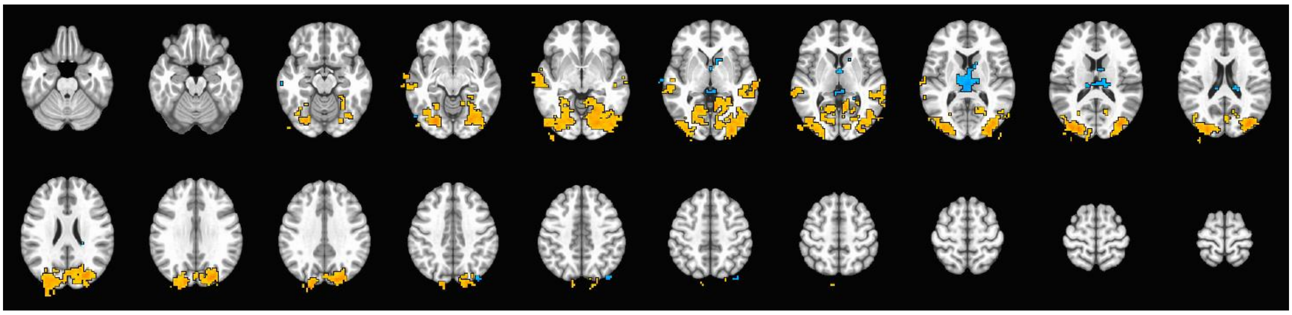

Figure S3.49

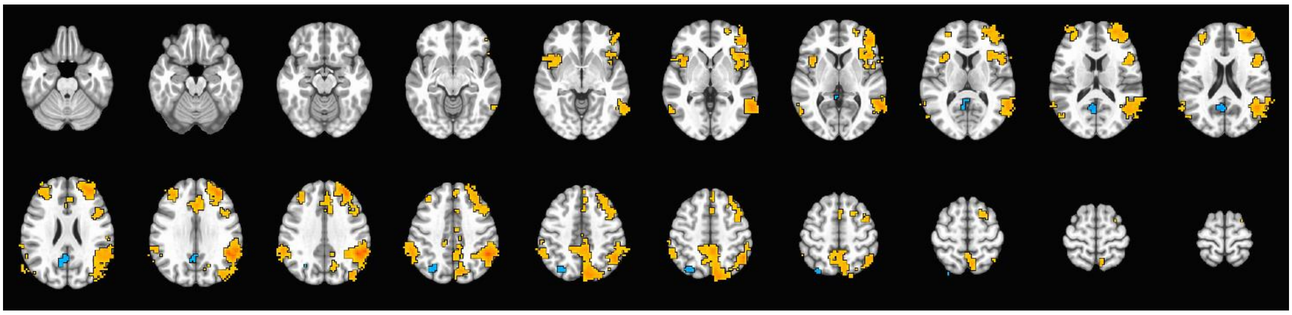

Figure S3.50

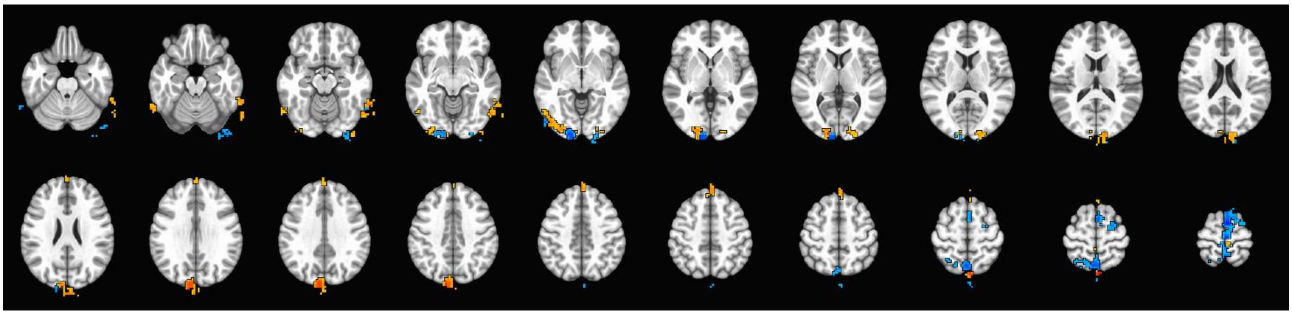

Figure S3.51

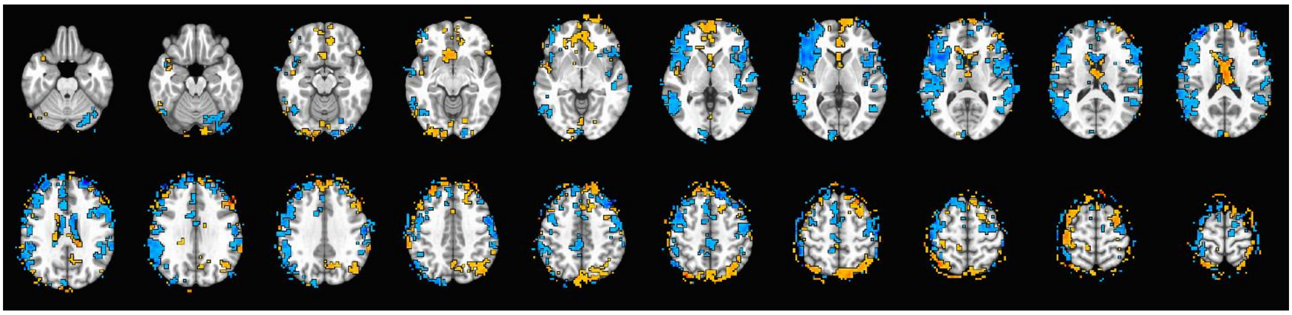

Figure S3.52

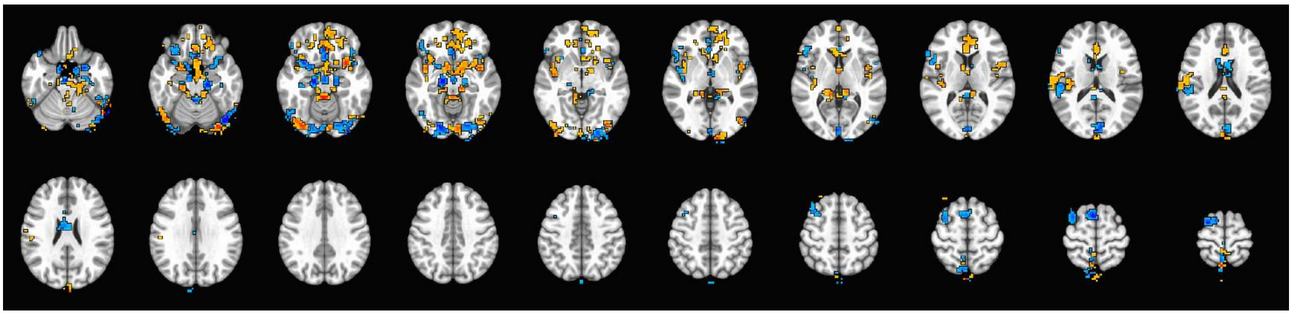

Figure S3.53

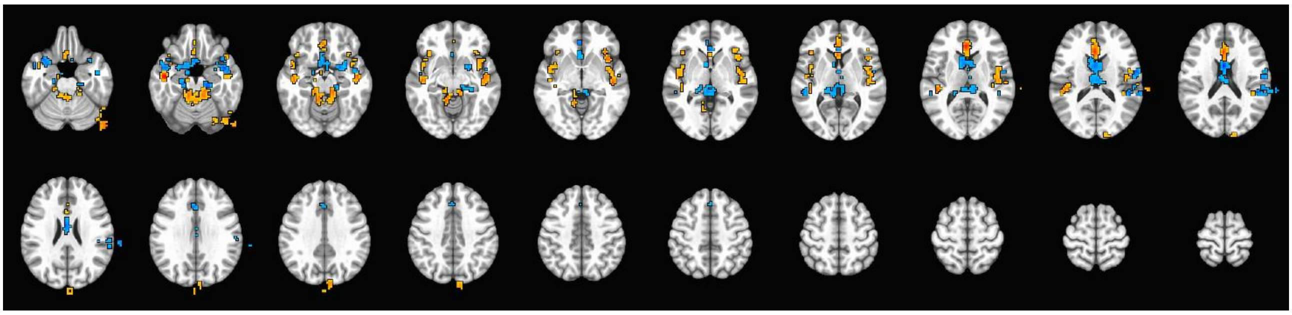

Figure S3.54

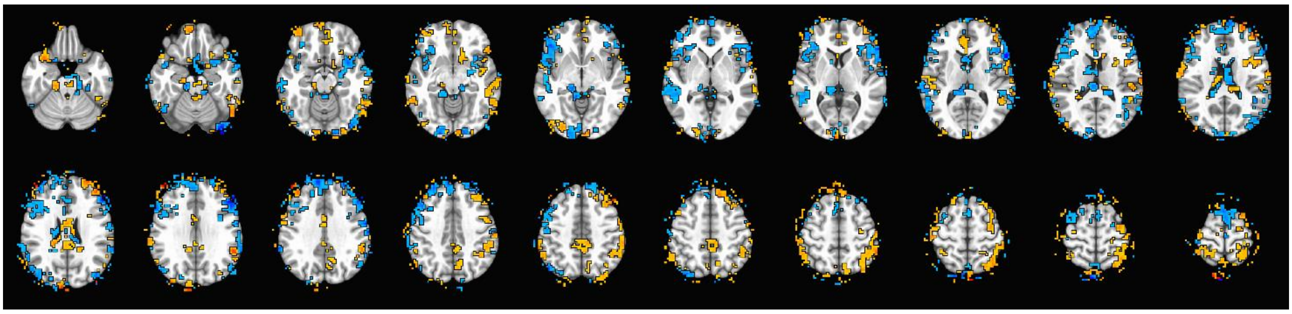

Figure S3.55

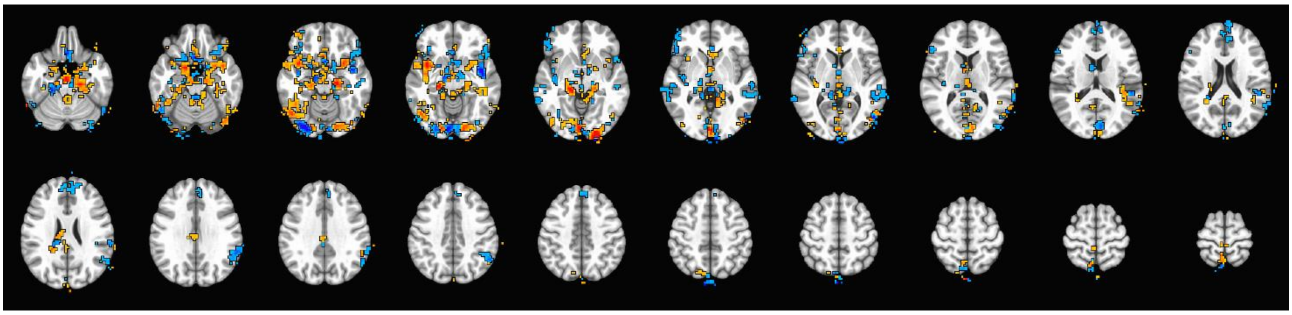

Figure S3.56

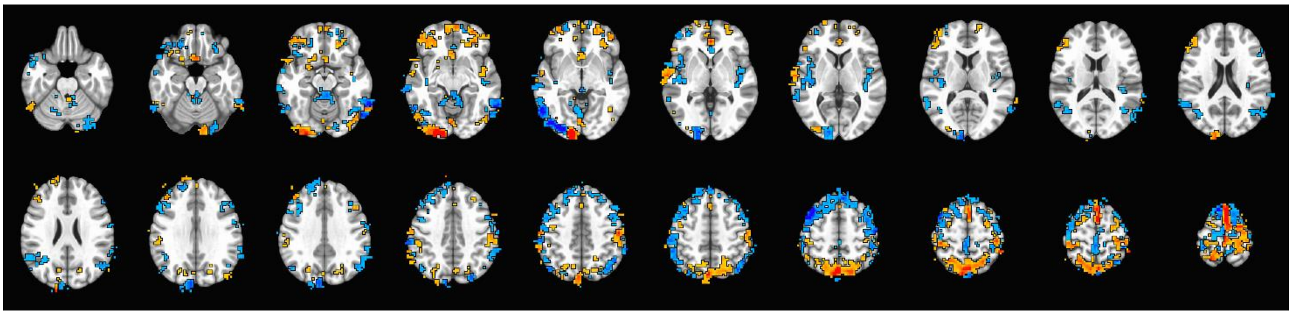

Figure S3.57

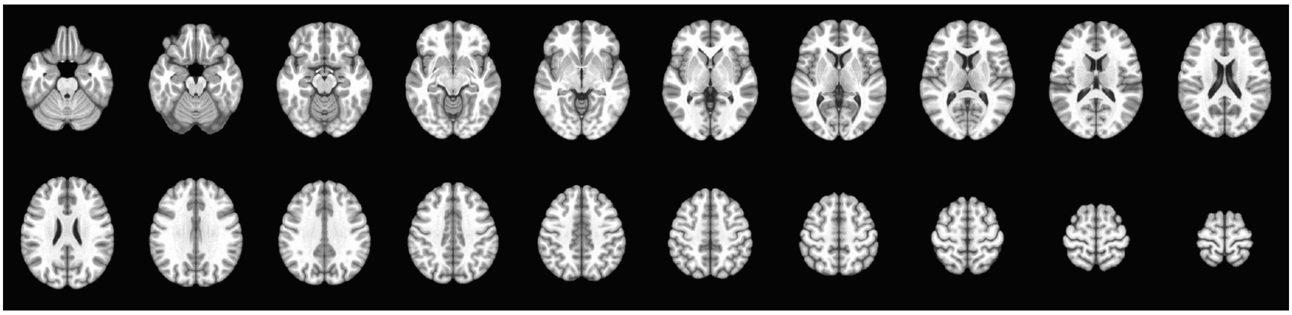

Figure S3.58

Group ICA at 1.5T with 4mm smoothing and Dimensionality 20

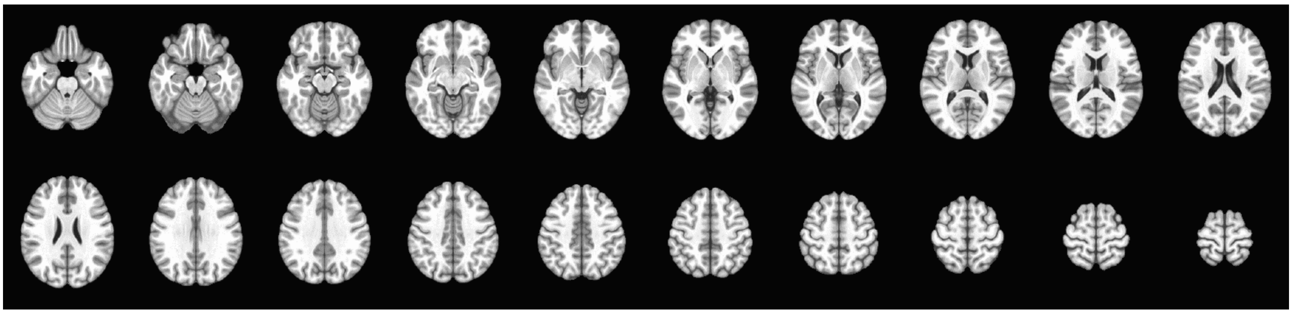

Figure S4.1

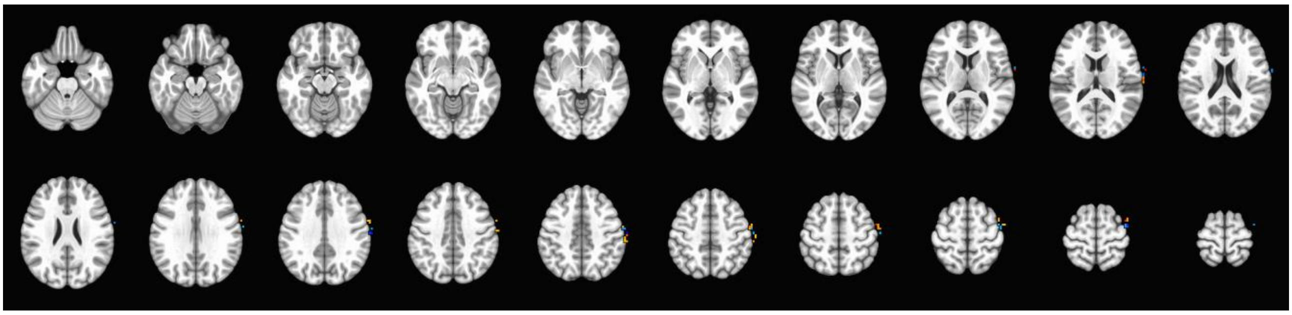

Figure S4.59

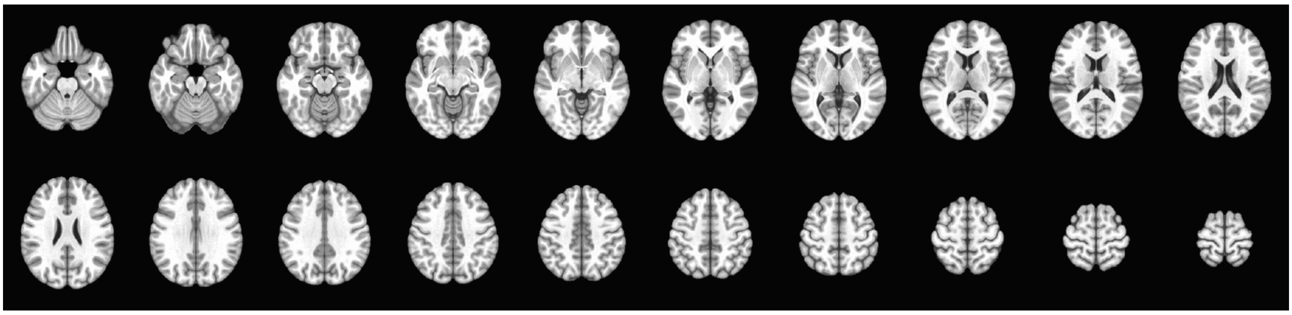

Figure S4.60

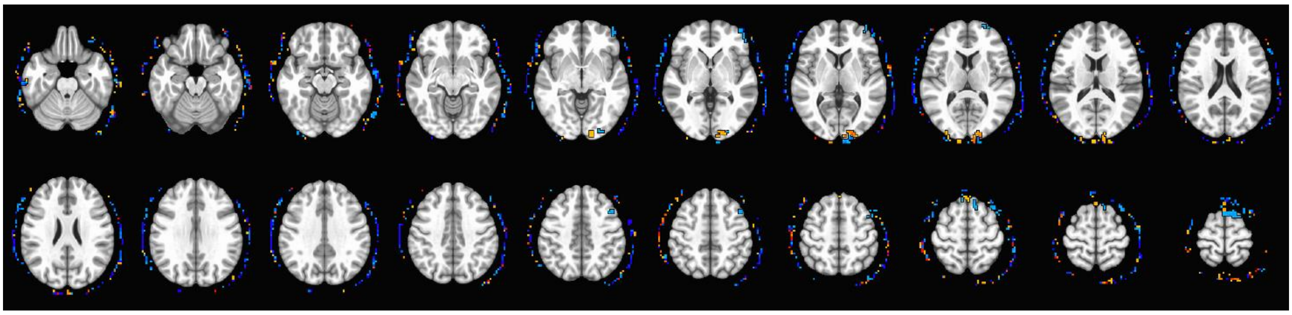

Figure S4.61

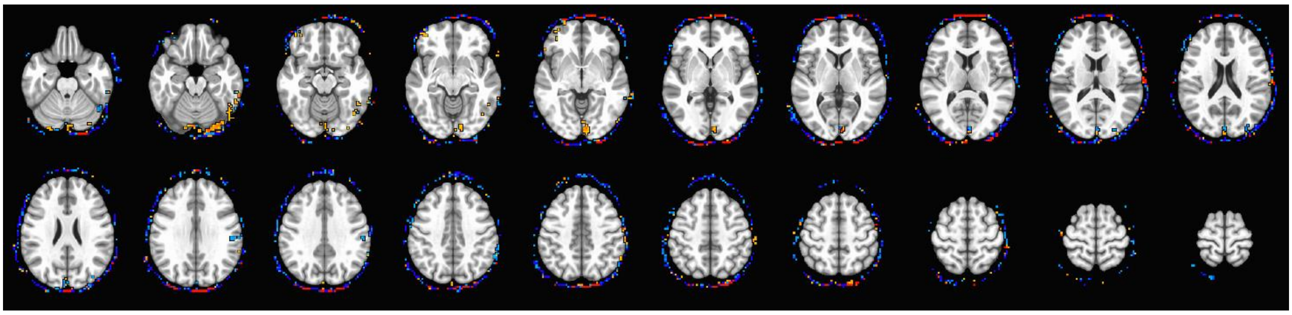

Figure S4.62

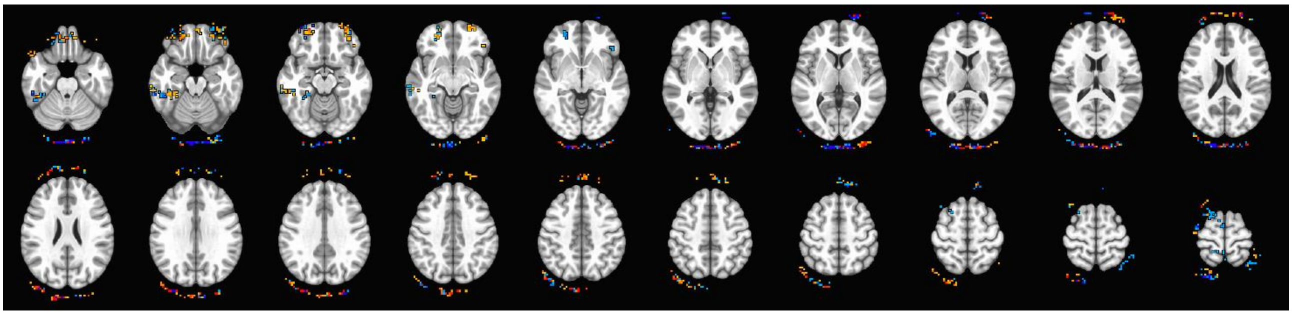

Figure S4.63

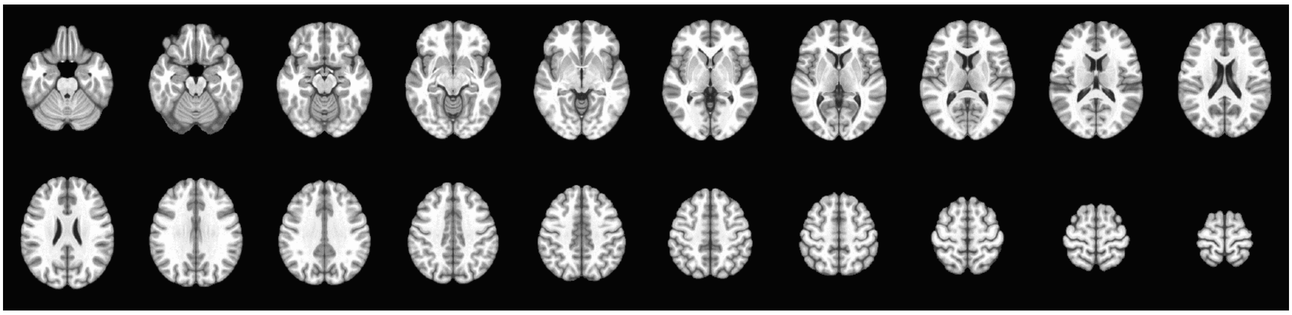

Figure S4.64

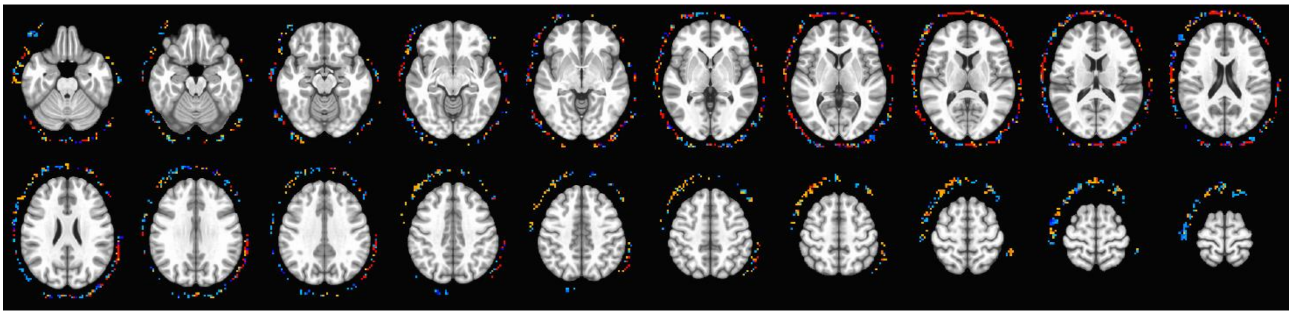

Figure S4.65

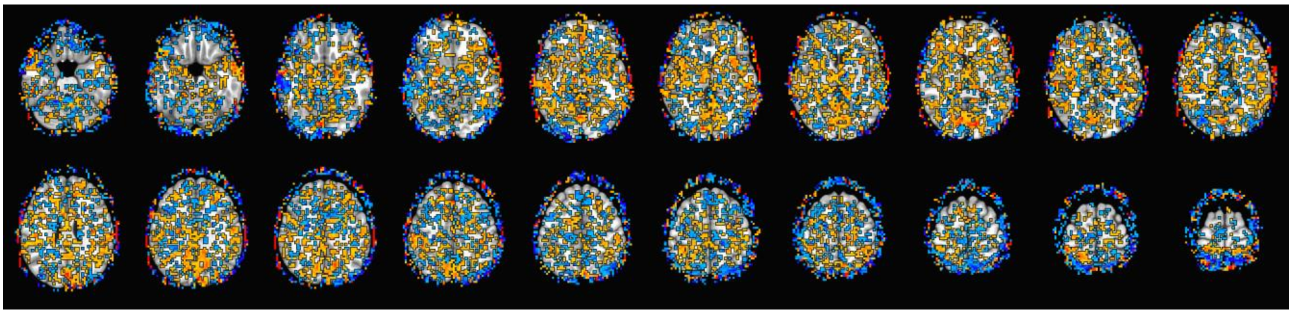

Figure S4.66

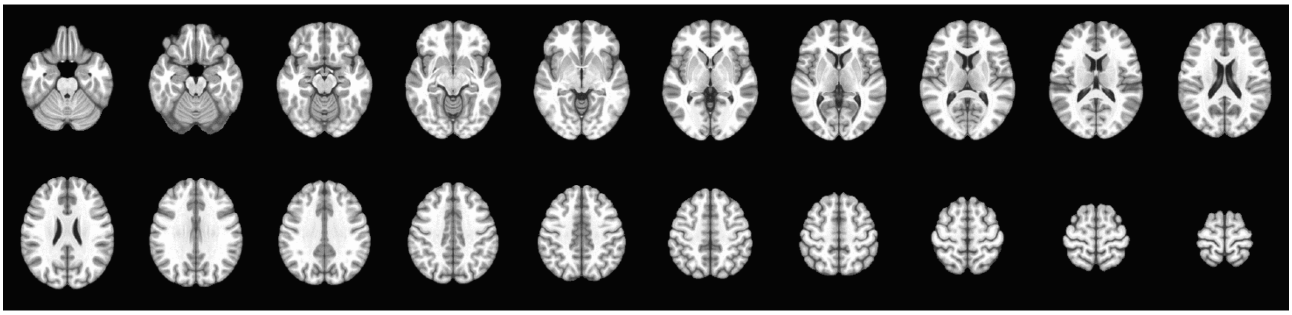

Figure S4.67

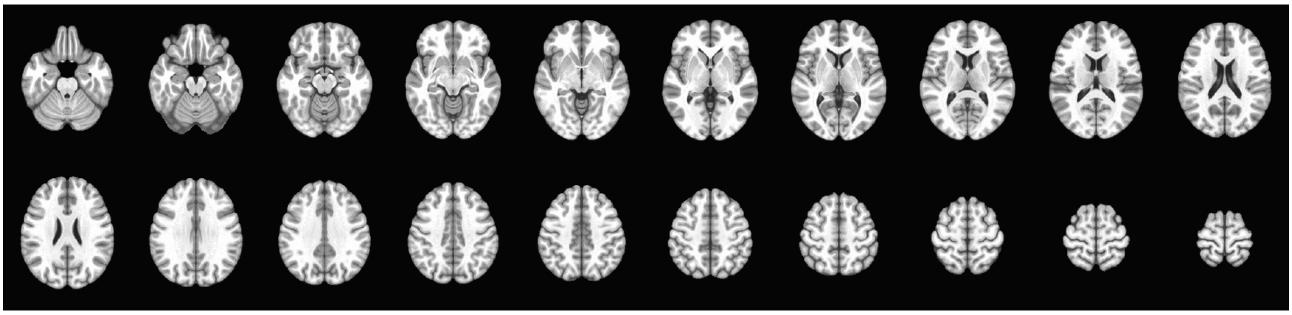

Figure S4.68

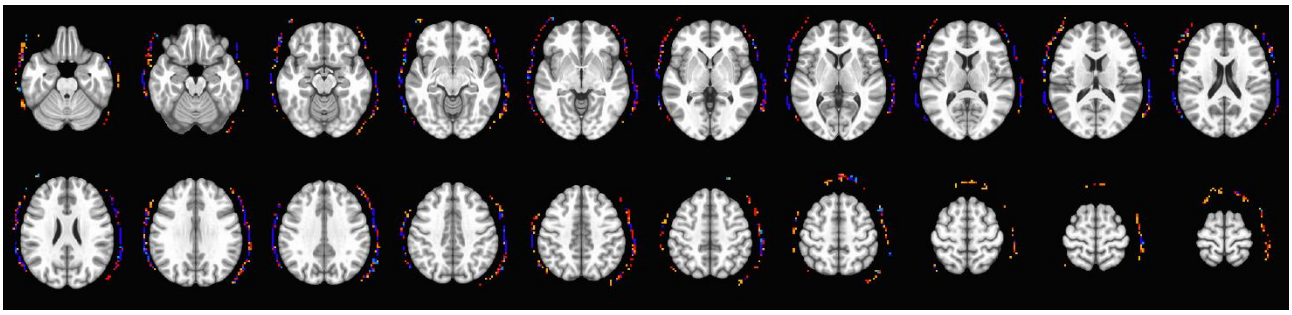

Figure S4.69

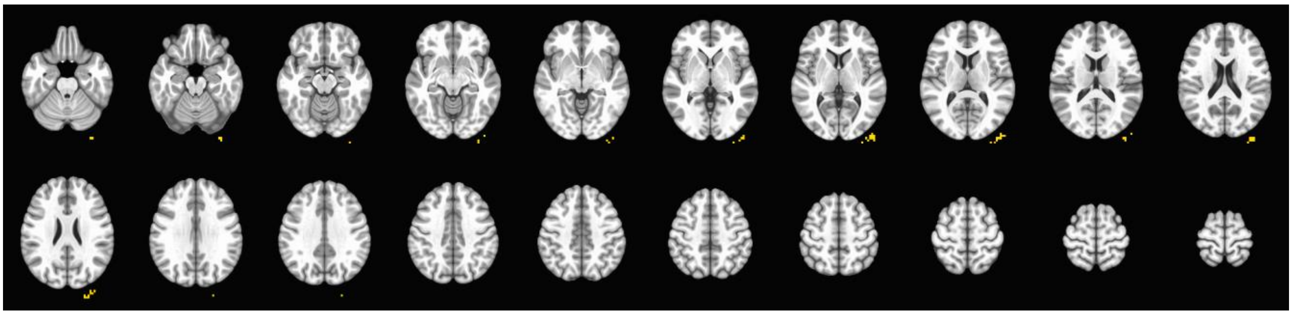

Figure S4.70

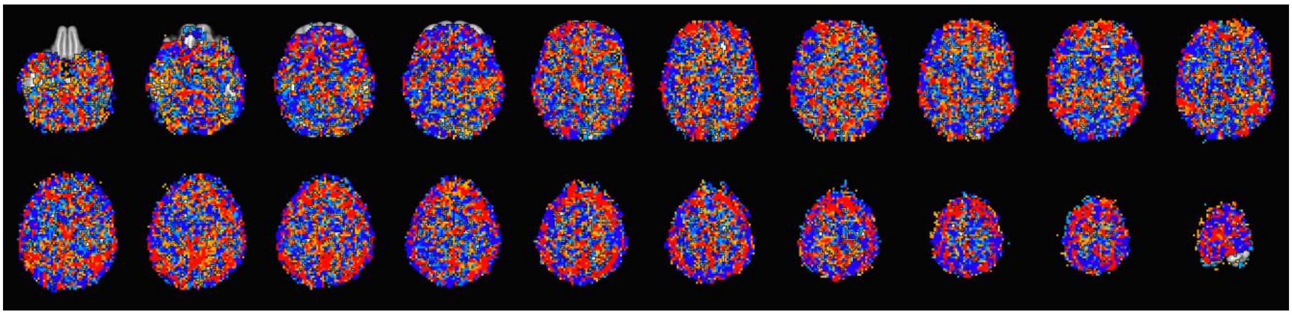

Figure S4.71

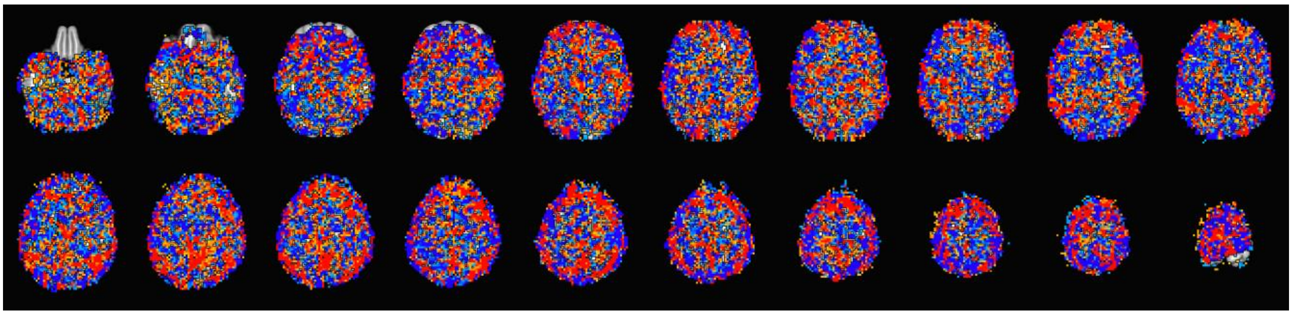

Figure S4.72

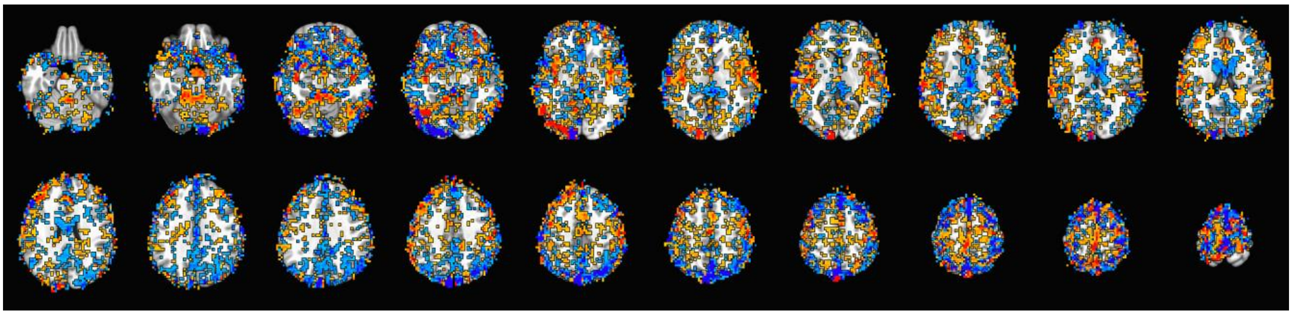

Figure S4.73

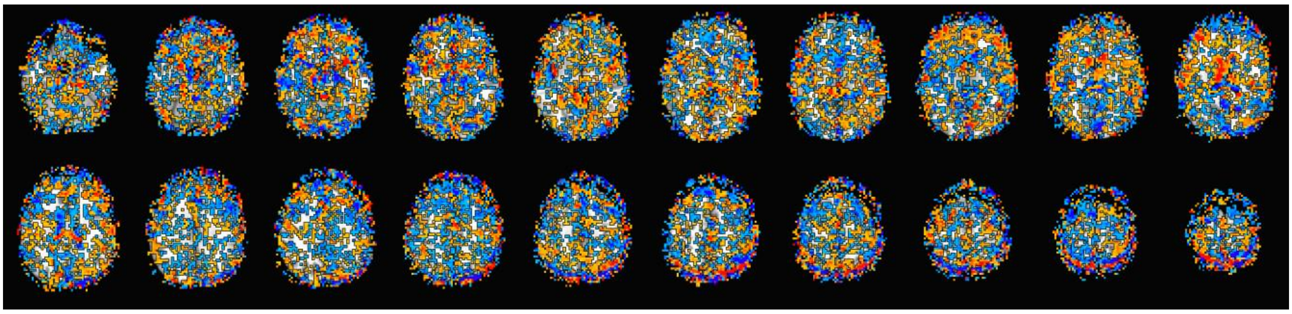

Figure S4.74

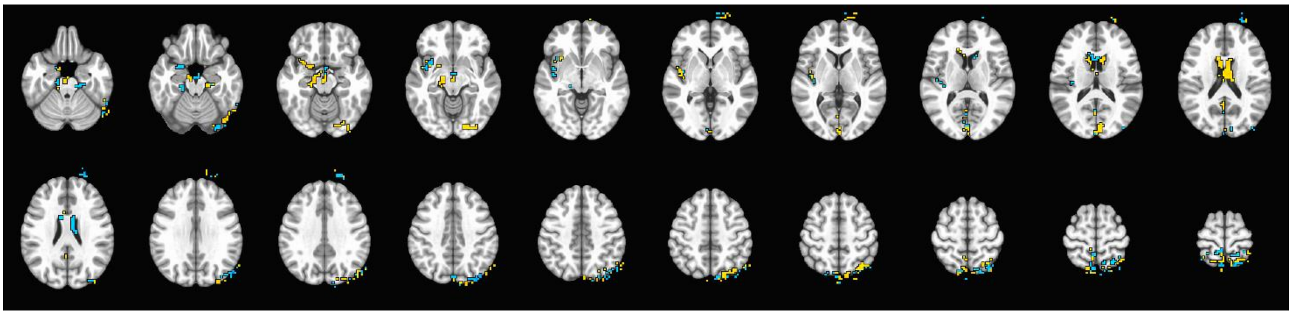

Figure S4.75

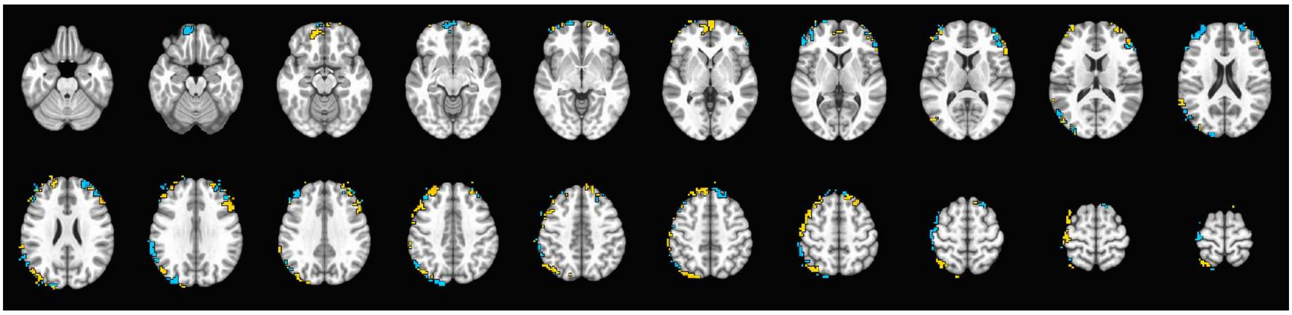

Figure S4.76

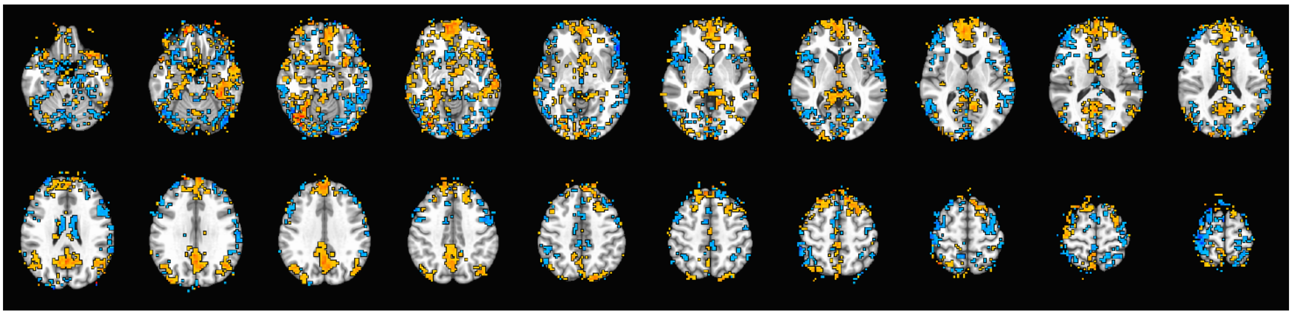

Figure S4.77

Group ICA at 3T with 12mm smoothing and Dimensionality 20

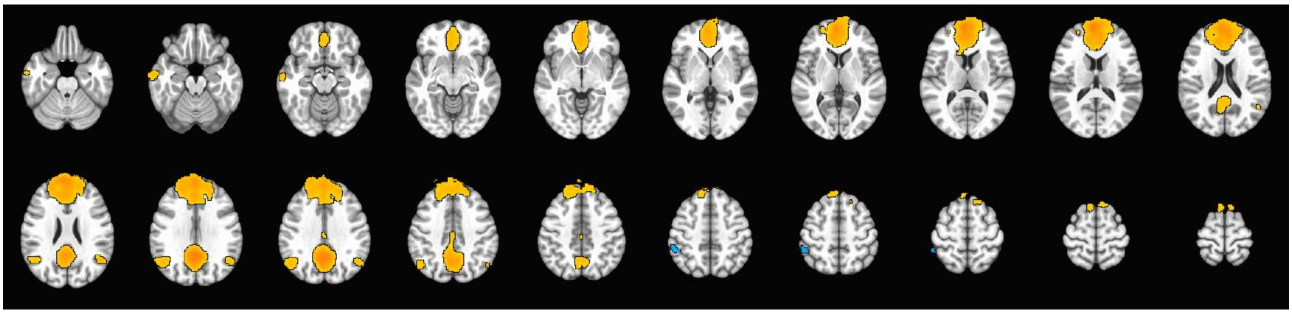

Figure S5.1

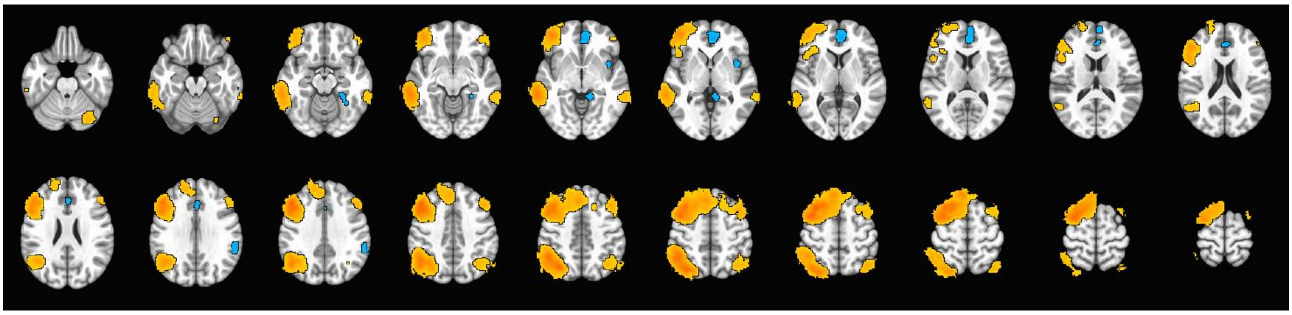

Figure S5.78

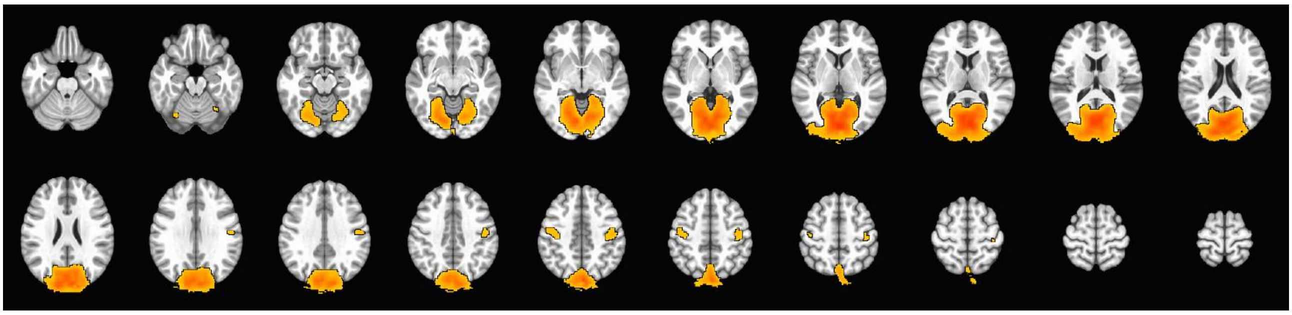

Figure S5.79

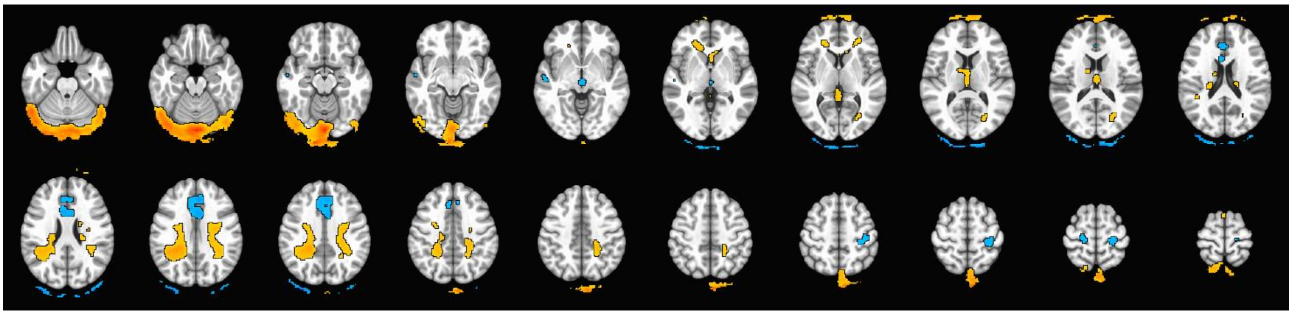

Figure S5.80

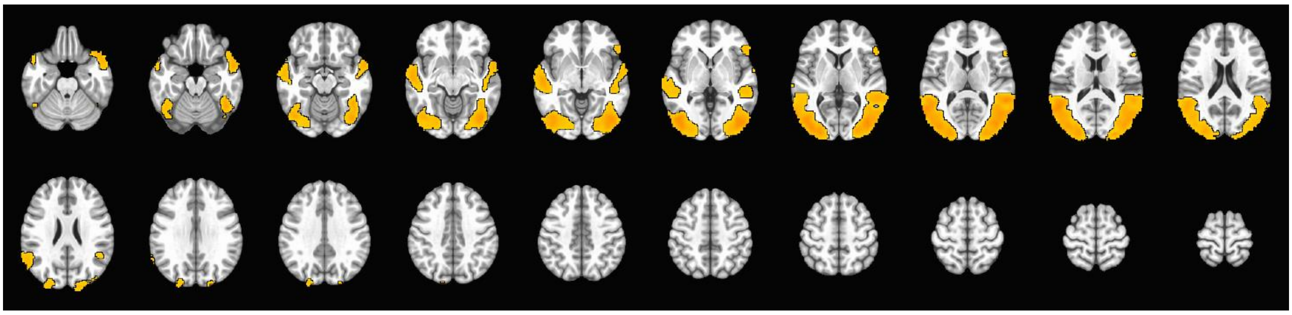

Figure S5.81

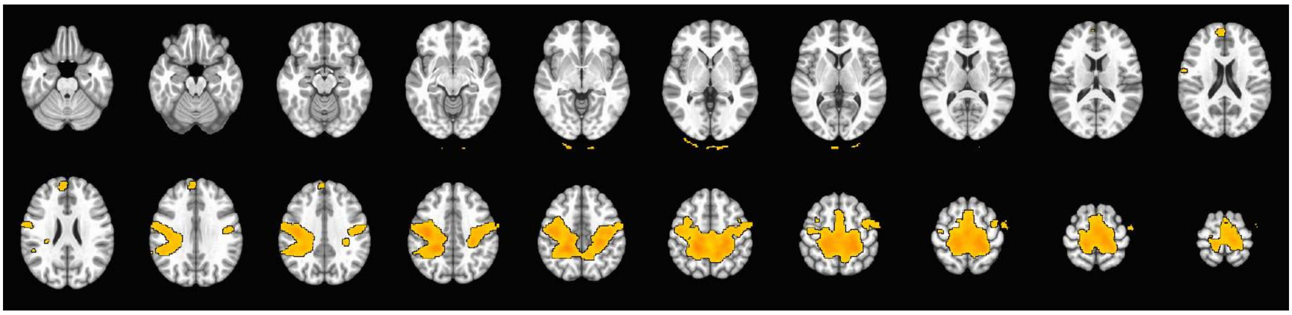

Figure S5.82

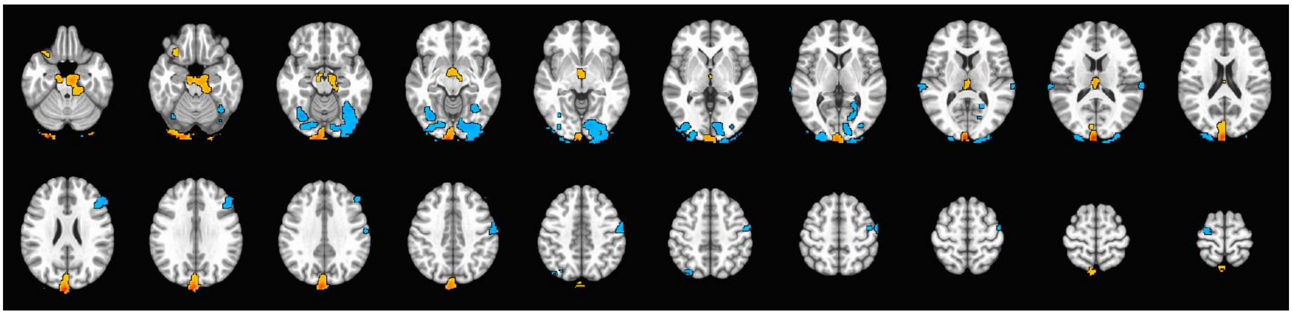

Figure S5.83

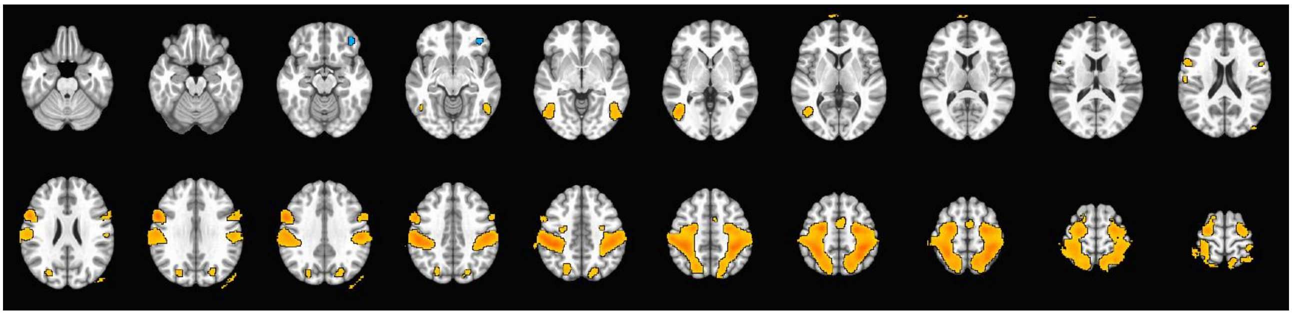

Figure S5.84

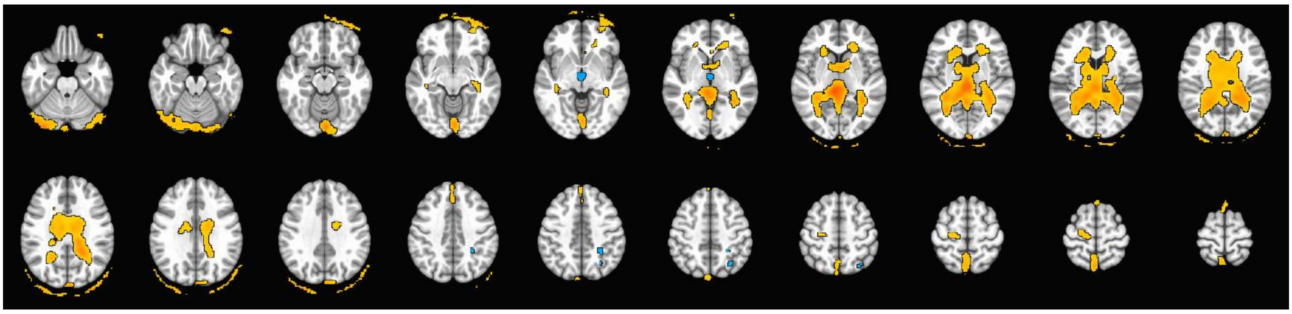

Figure S5.85

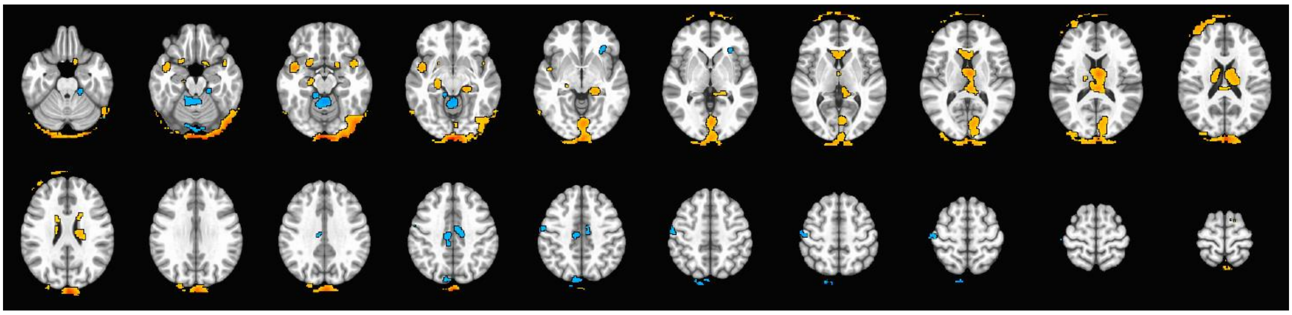

Figure S5.86

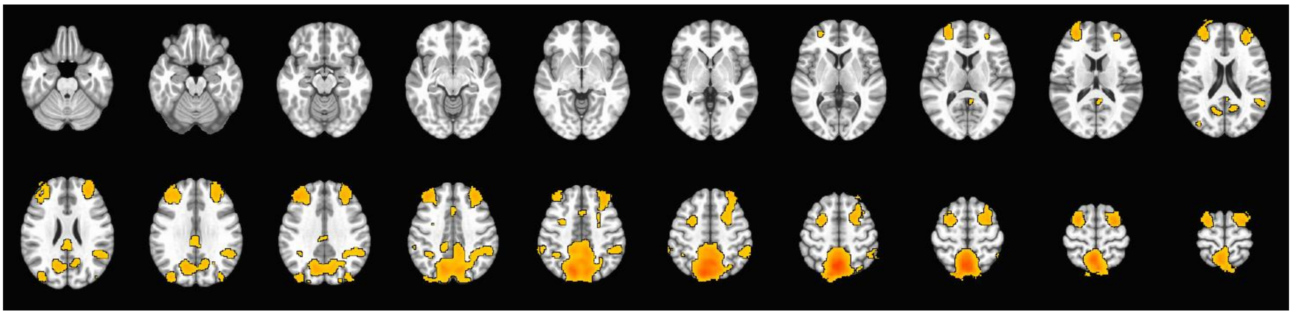

Figure S5.87

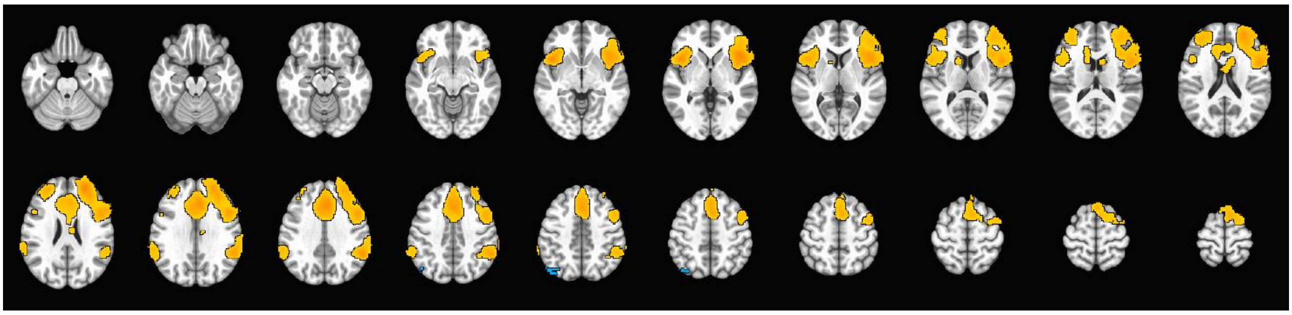

Figure S5.88

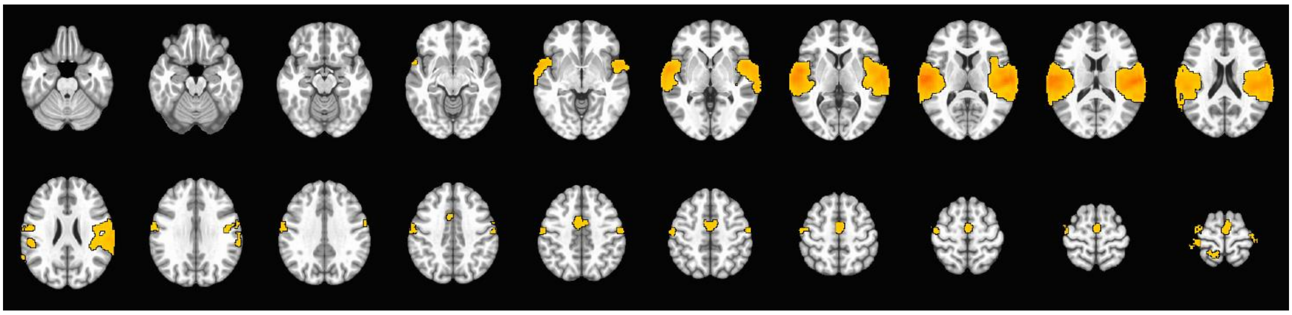

Figure S5.89

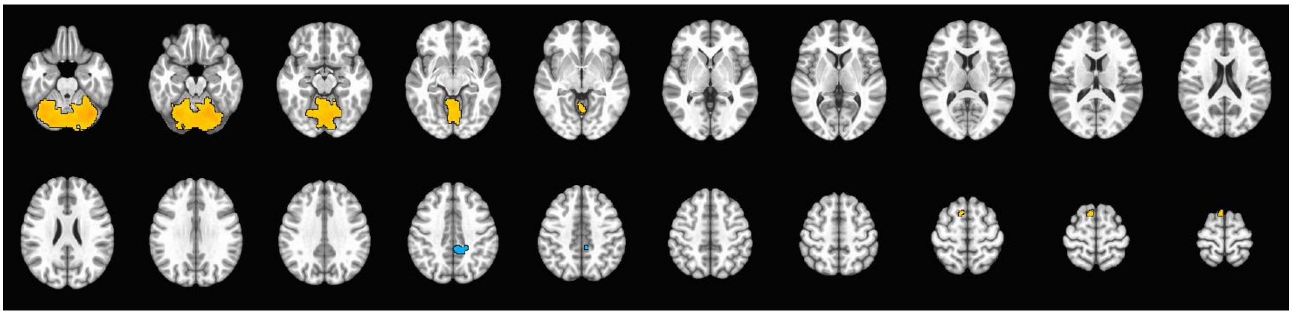

Figure S5.90

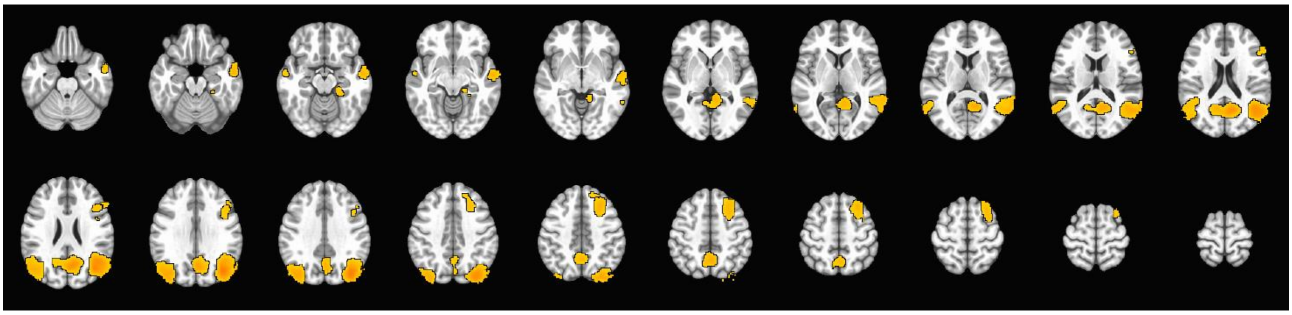

Figure S5.91

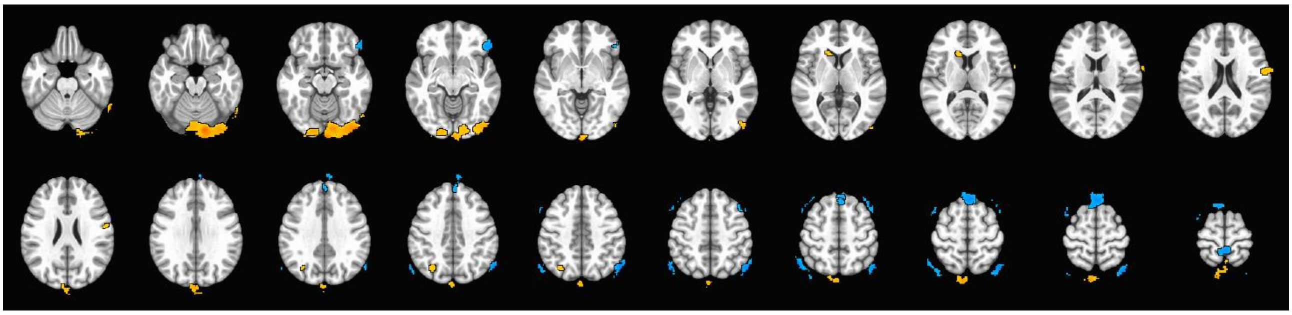

Figure S5.92

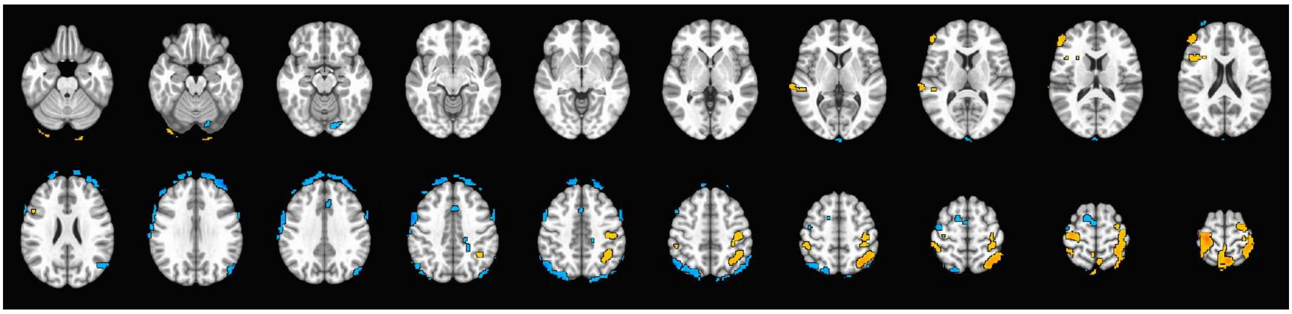

Figure S5.93

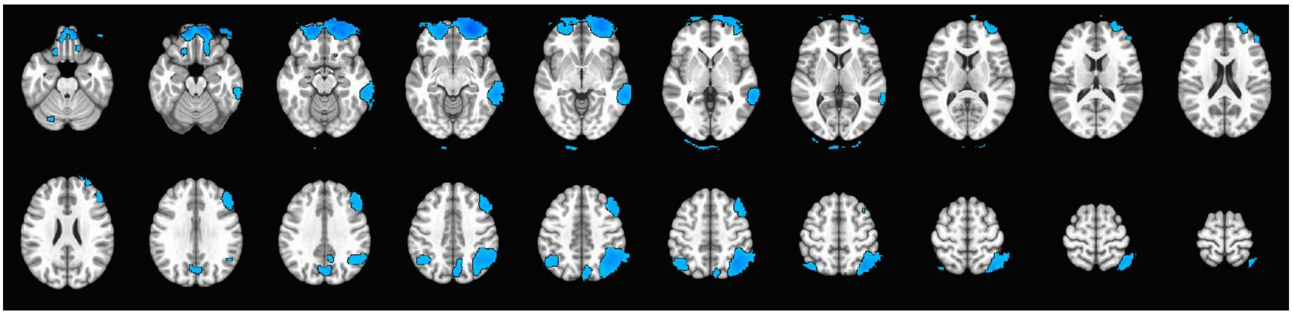

Figure S5.94

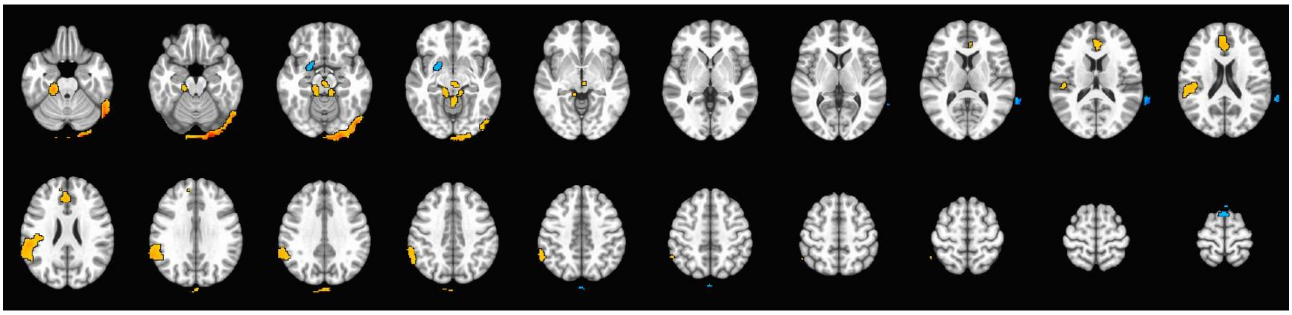

Figure S5.95

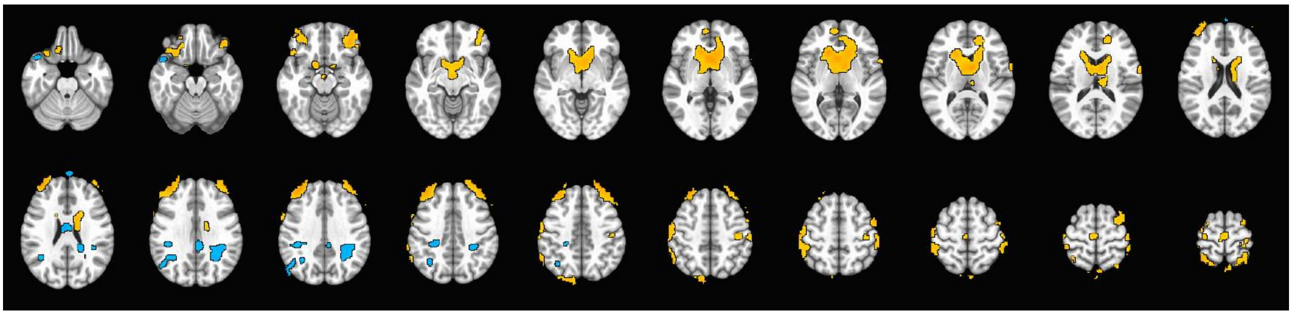

Figure S5.96

Group ICA at 3T with 8mm smoothing and Dimensionality 20

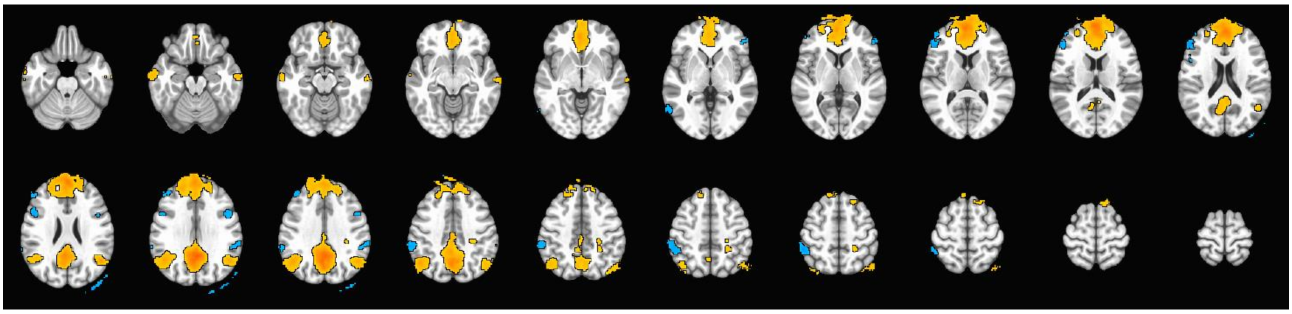

Figure S6.1

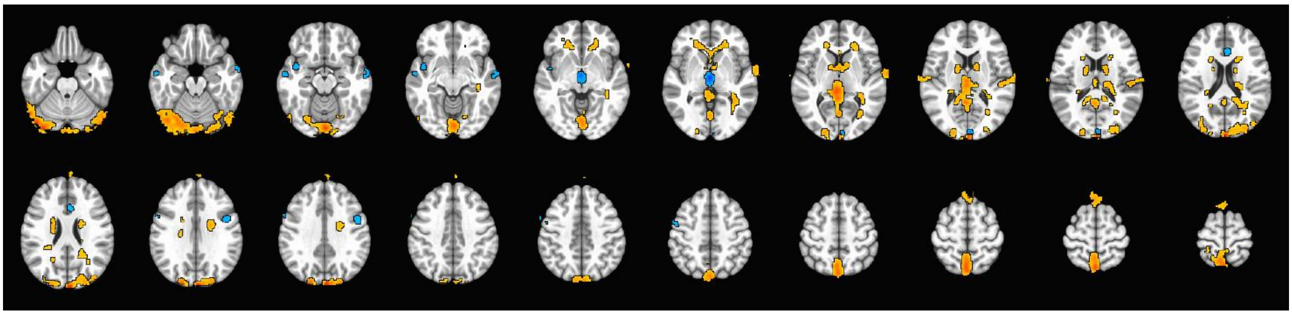

Figure S6.97

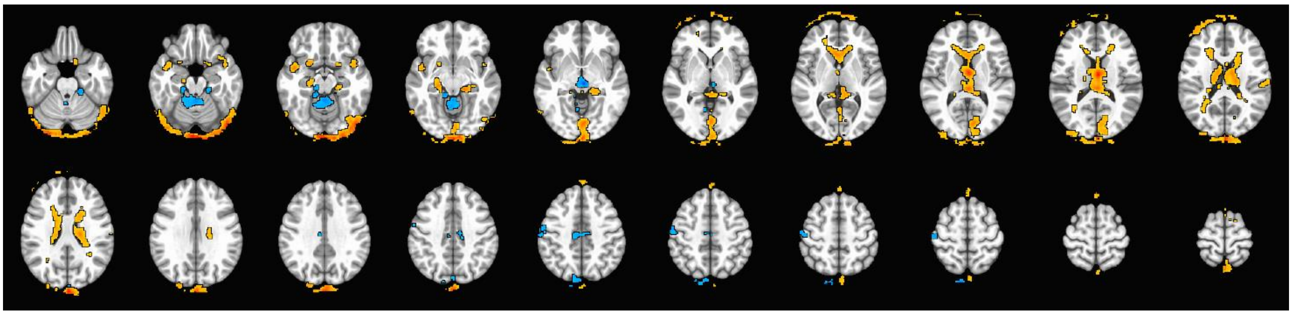

Figure S6.98

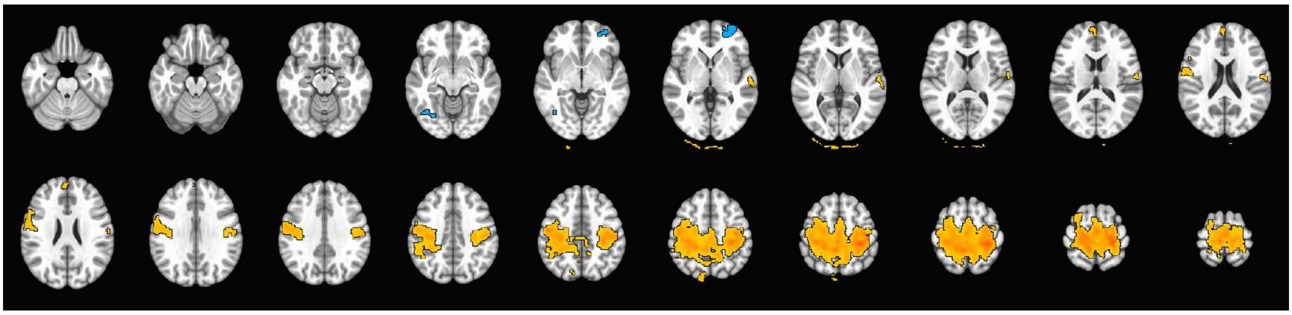

Figure S6.99

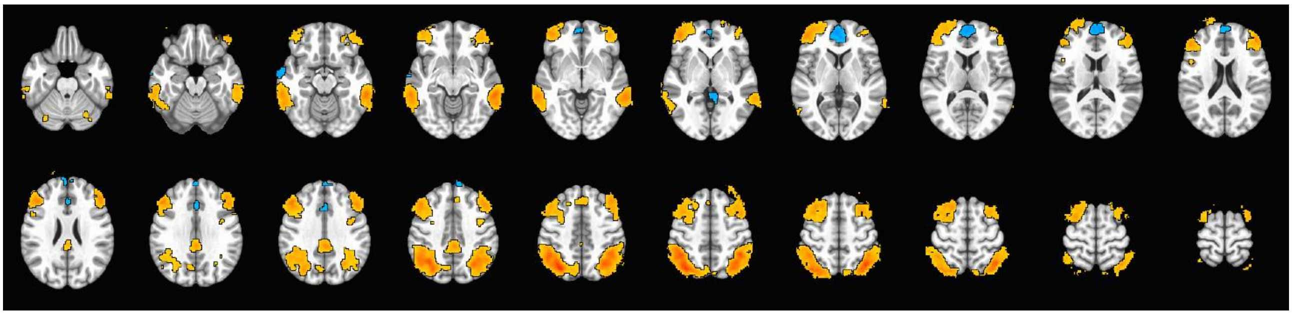

Figure S6.100

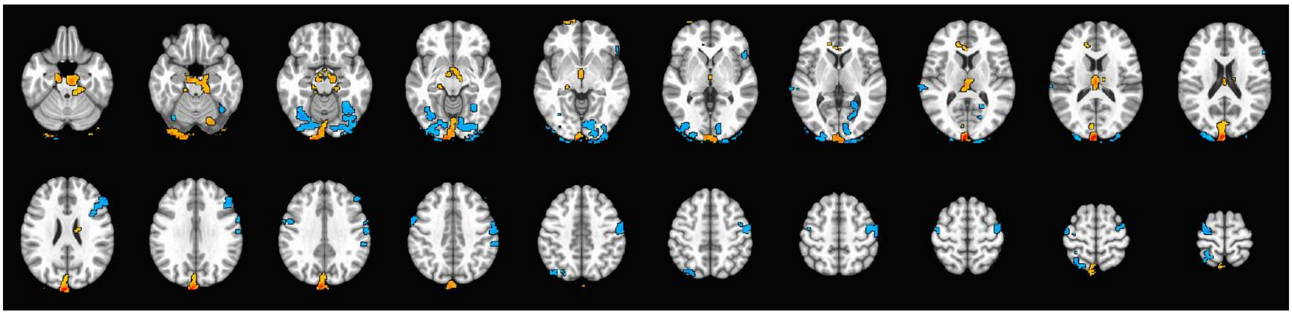

Figure S6.101

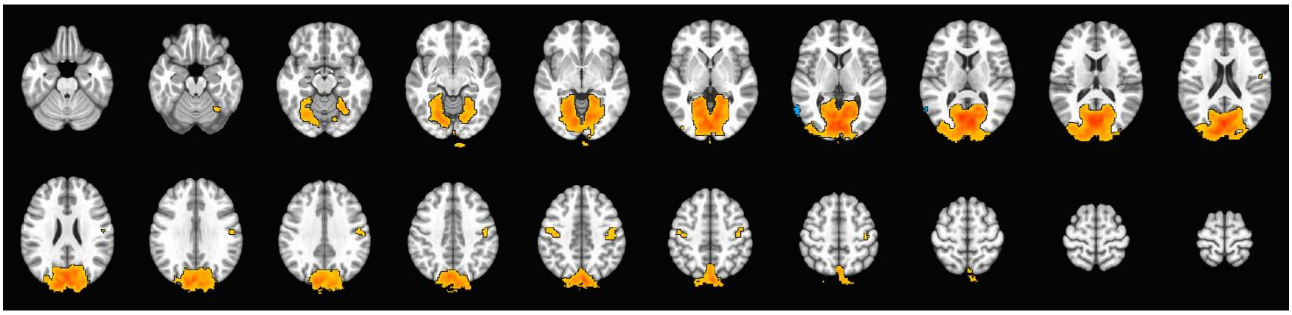

Figure S6.102

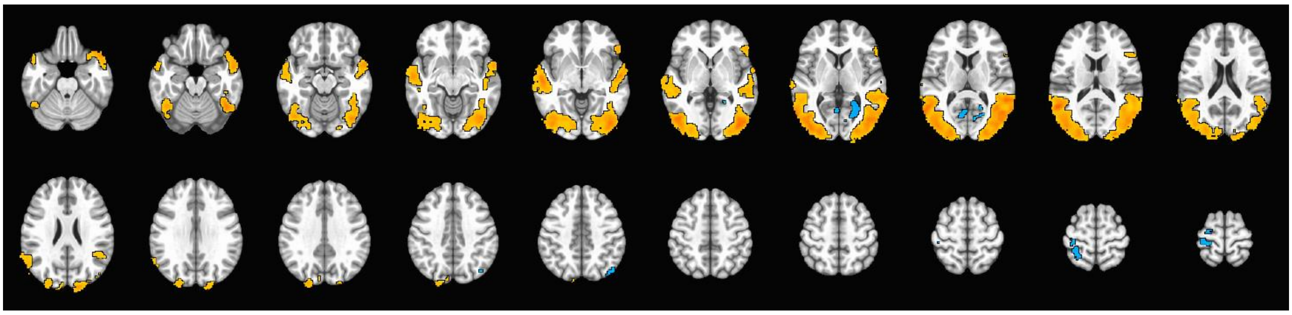

Figure S6.103

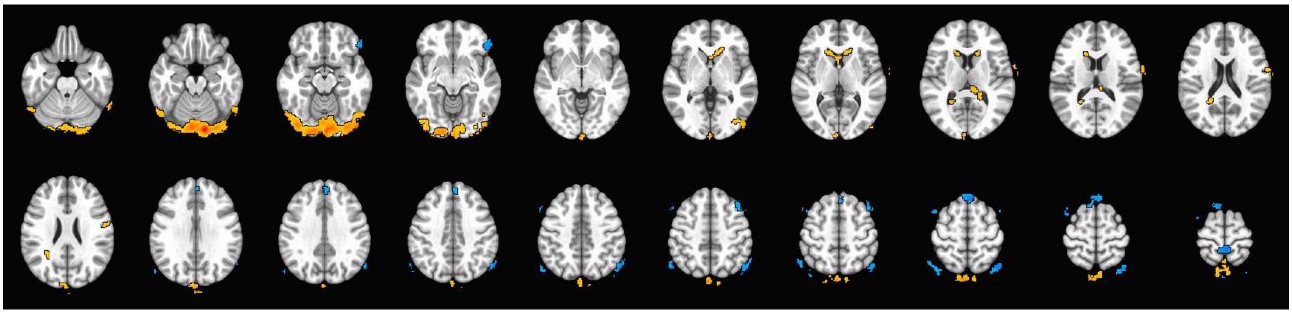

Figure S6.104

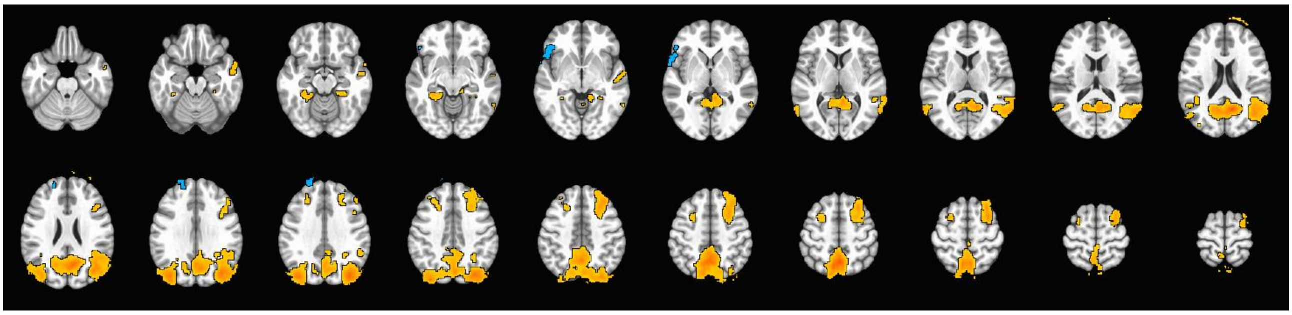

Figure S6.105

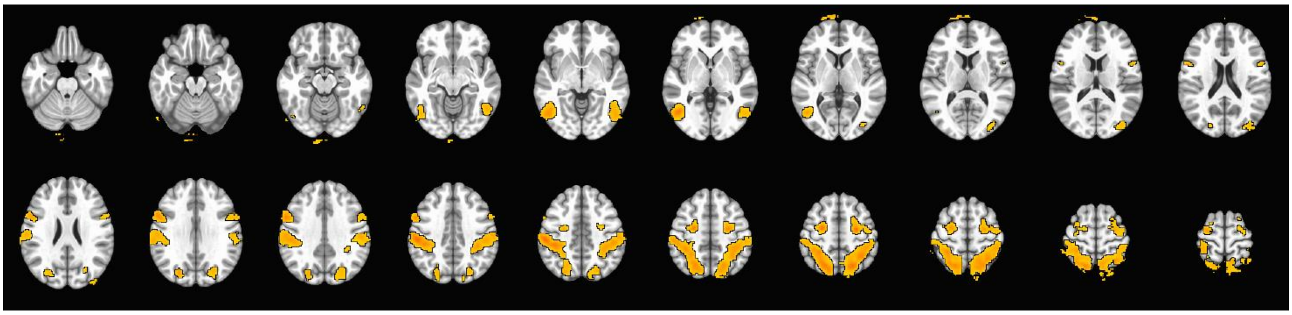

Figure S6.106

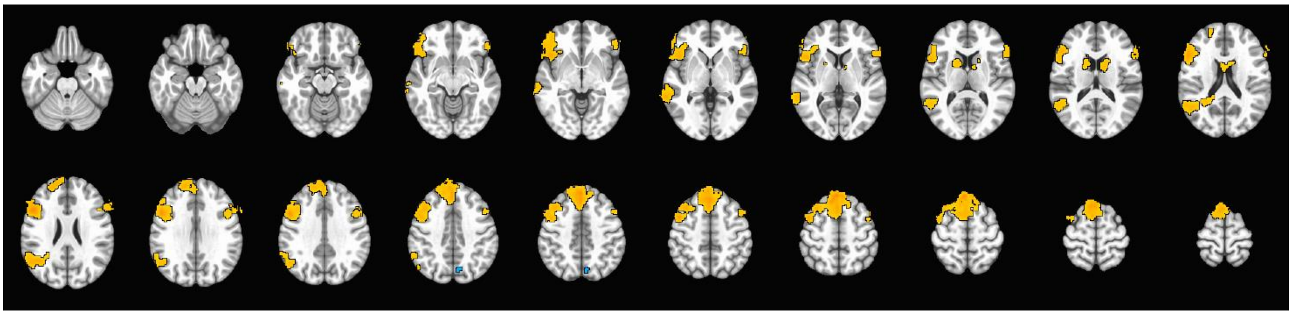

Figure S6.107

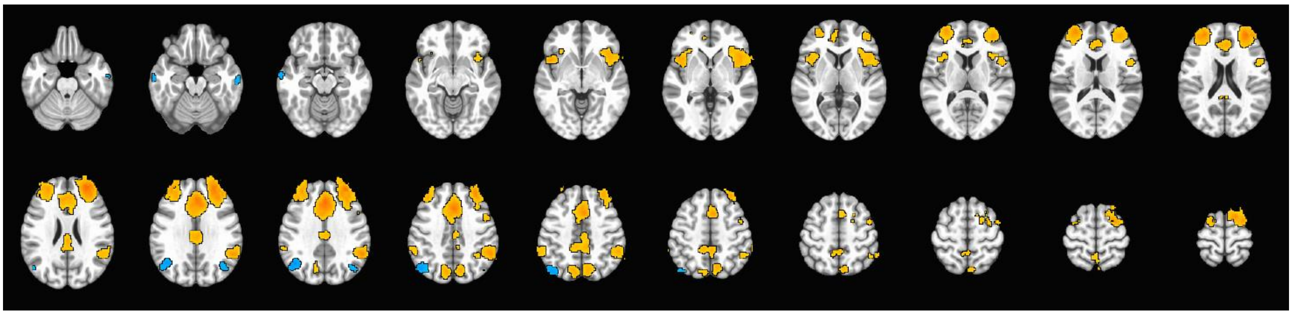

Figure S6.108

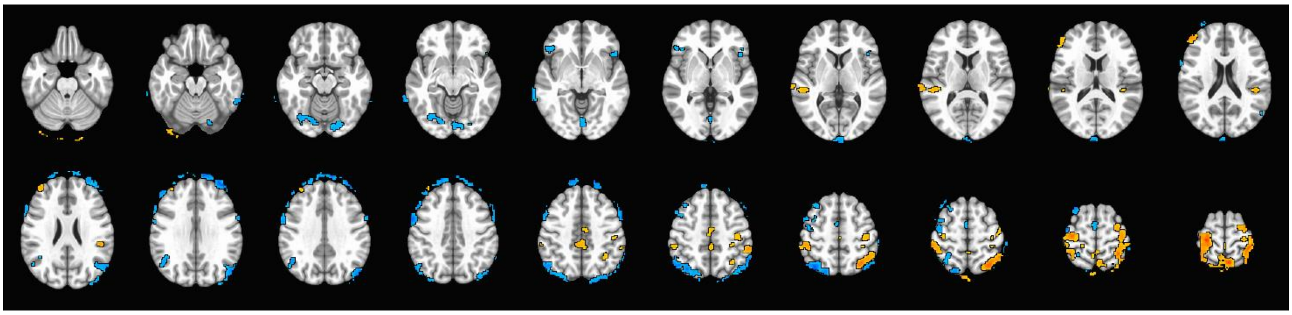

Figure S6.109

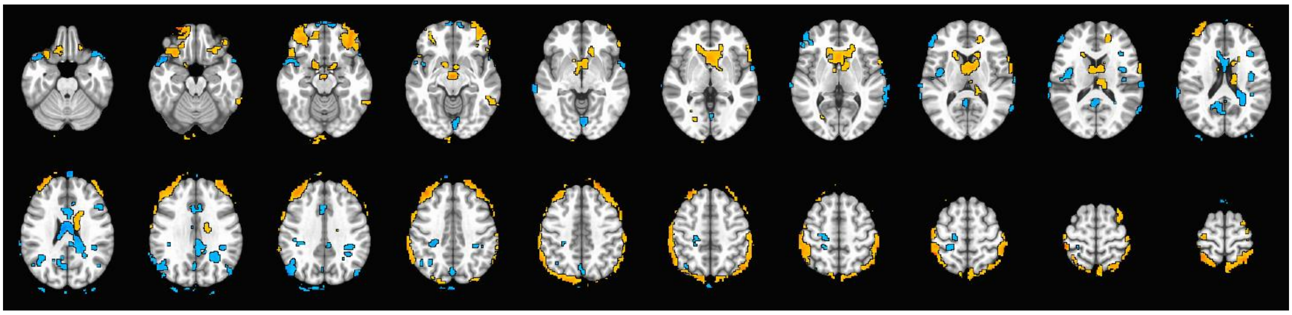

Figure S6.110

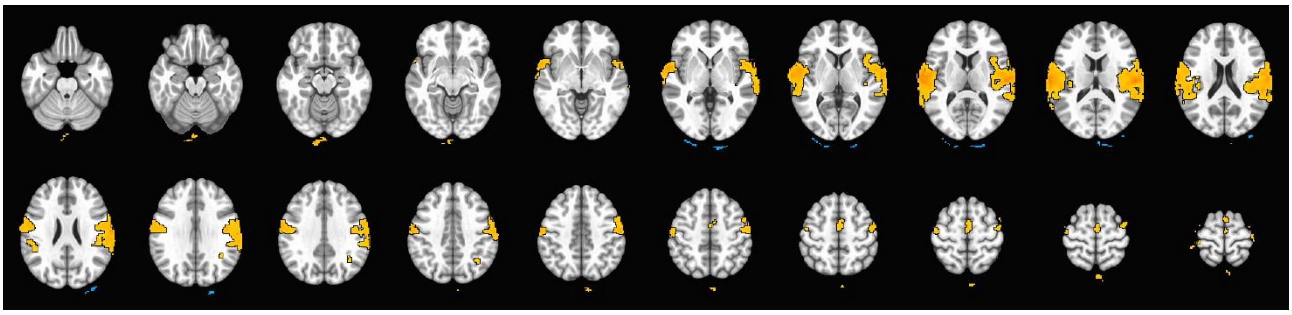

Figure S6.111

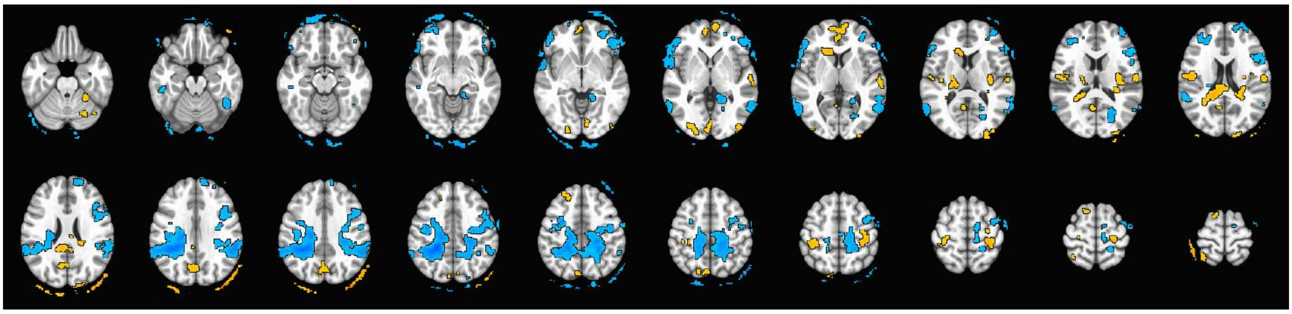

Figure S6.112

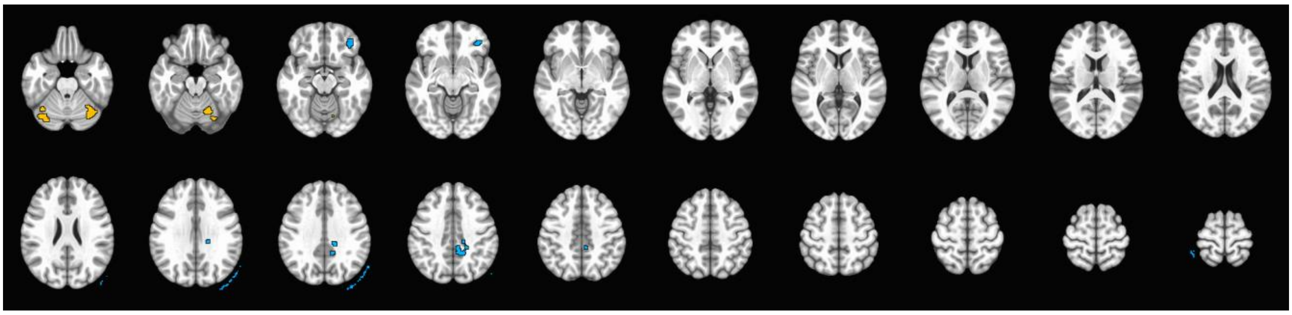

Figure S6.113

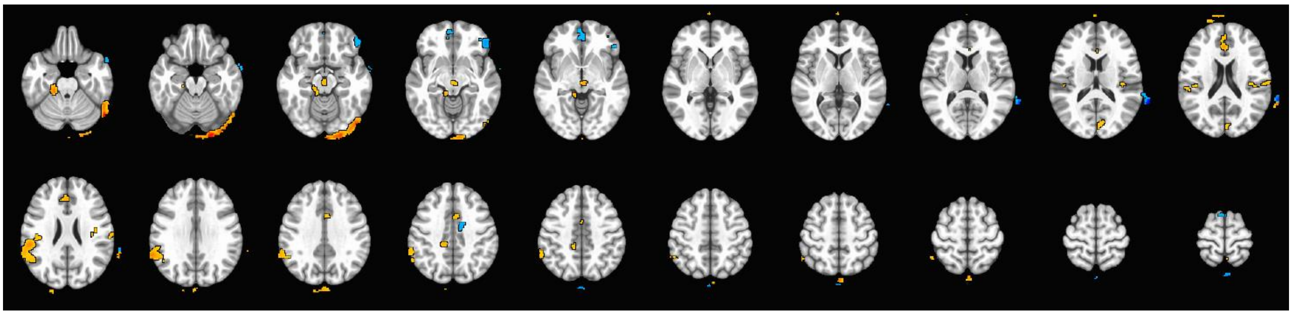

Figure S6.114

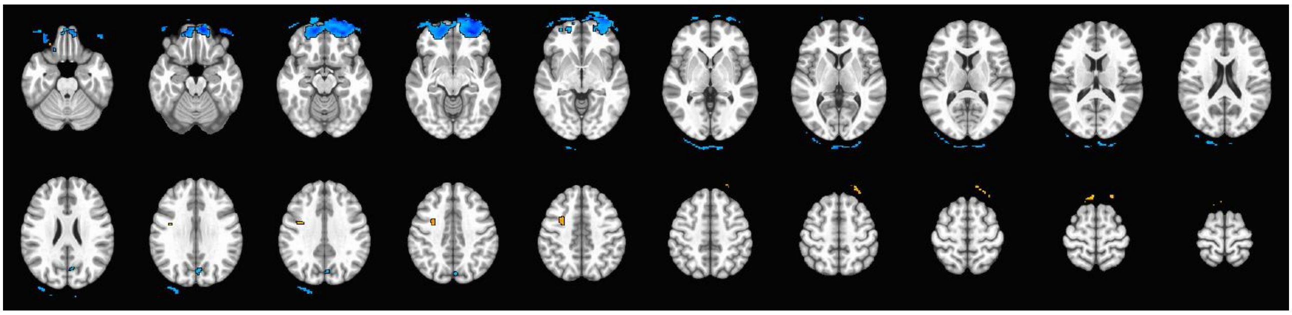

Figure S6.115

Group ICA at 3T with 6mm smoothing and Dimensionality 20

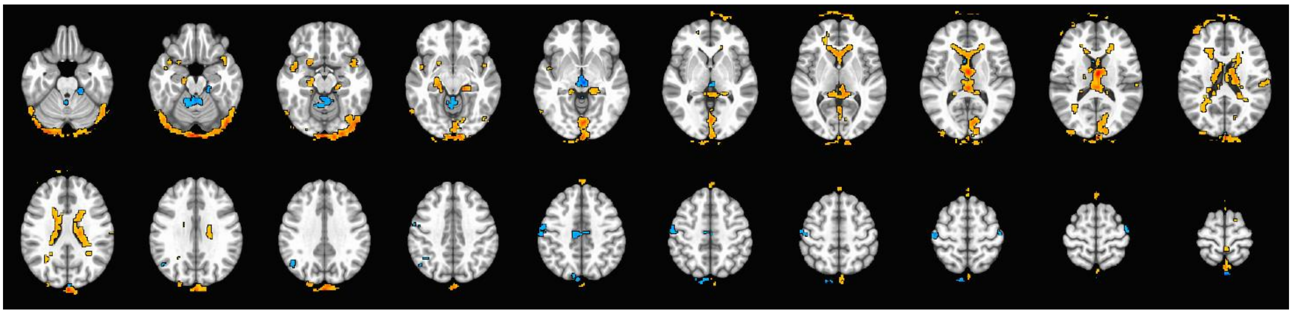

Figure S7.1

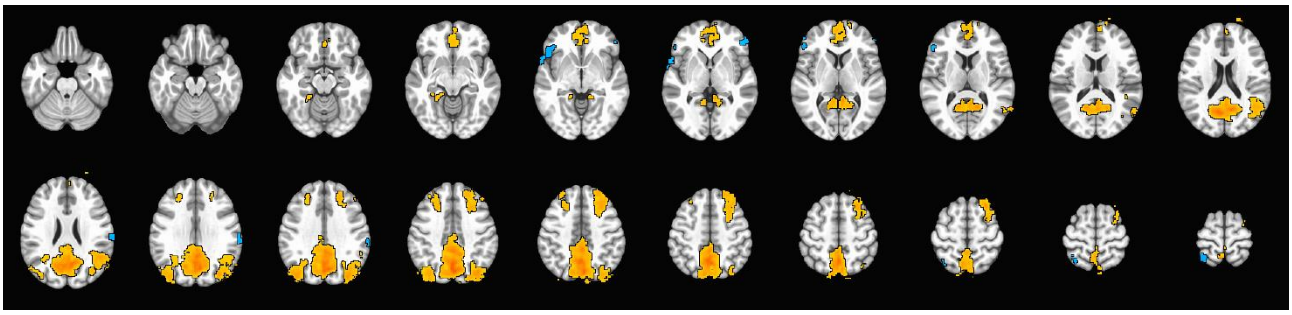

Figure S7.116

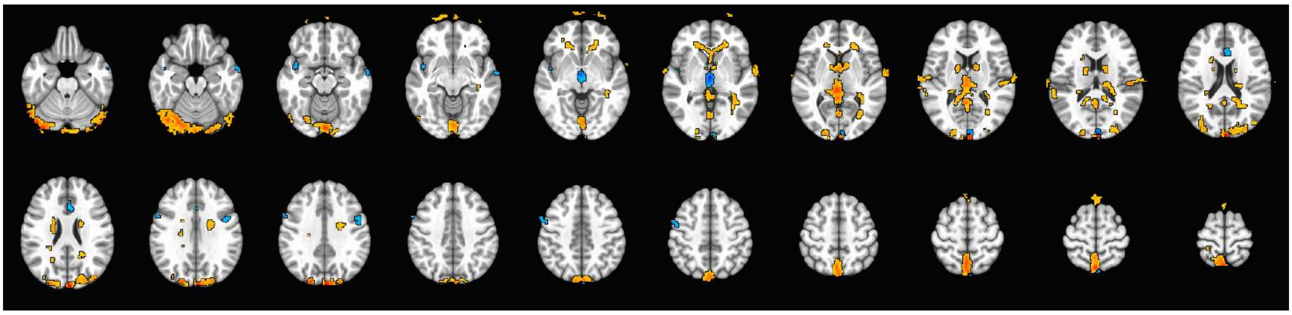

Figure S7.117

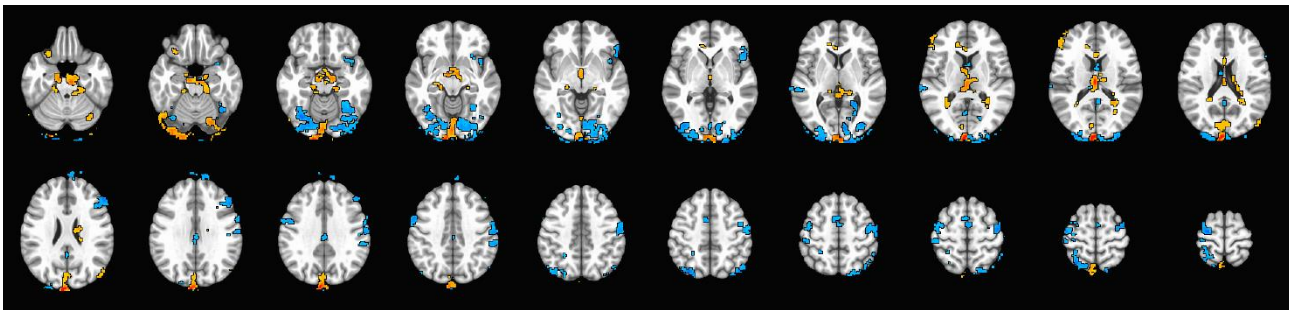

Figure S7.118

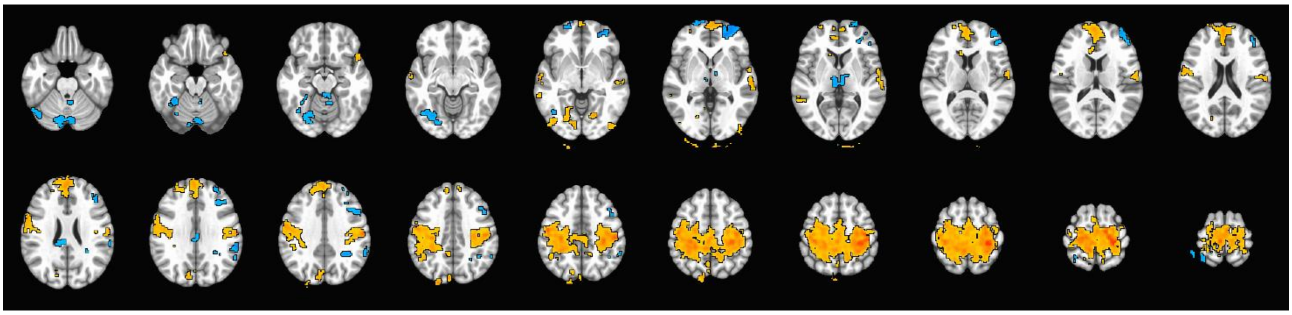

Figure S7.119

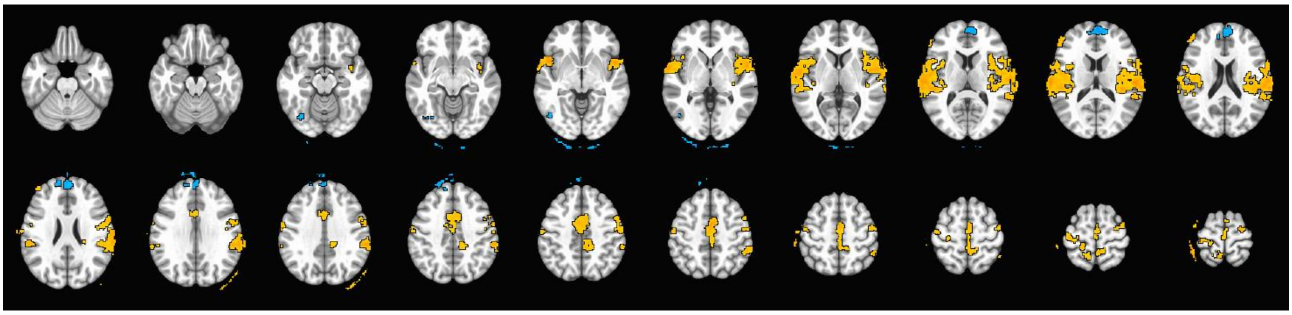

Figure S7.120

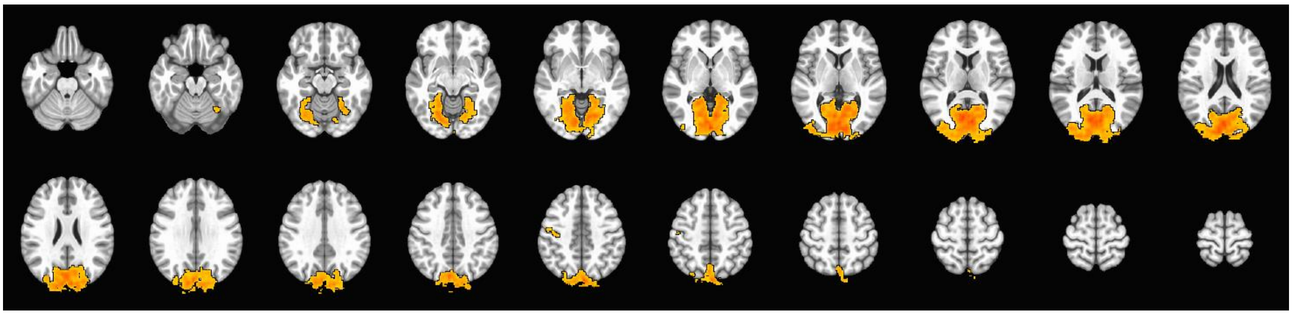

Figure S7.121

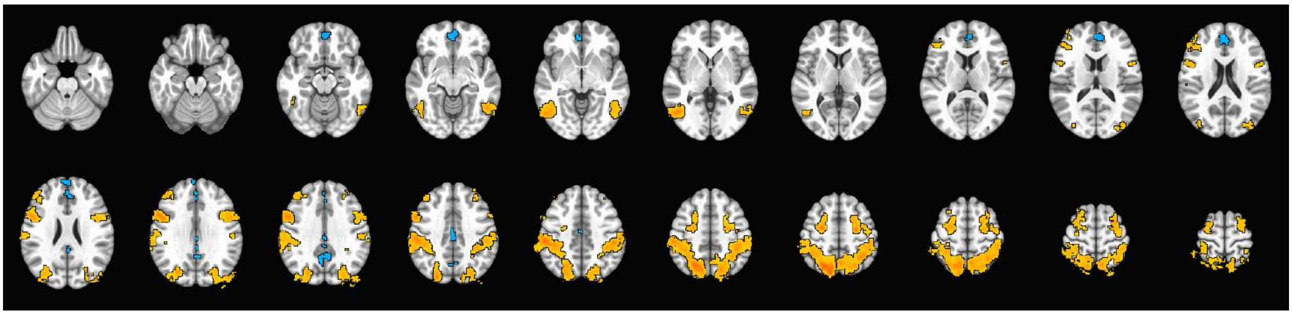

Figure S7.122

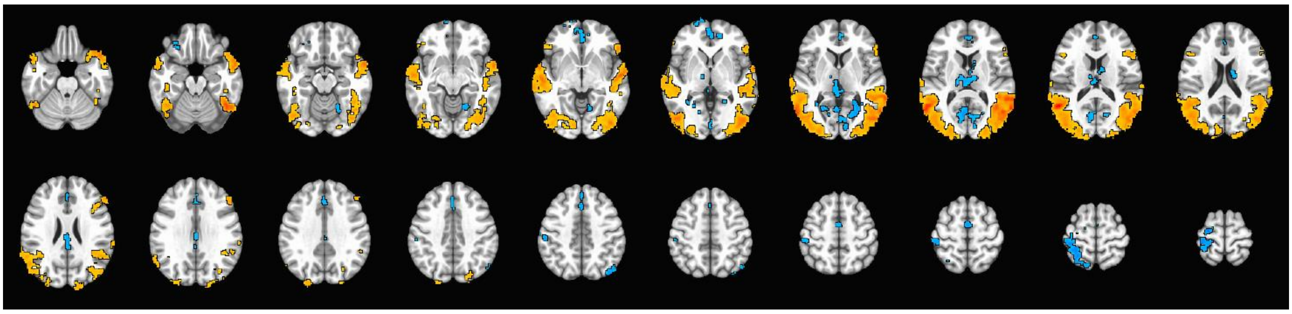

Figure S7.123

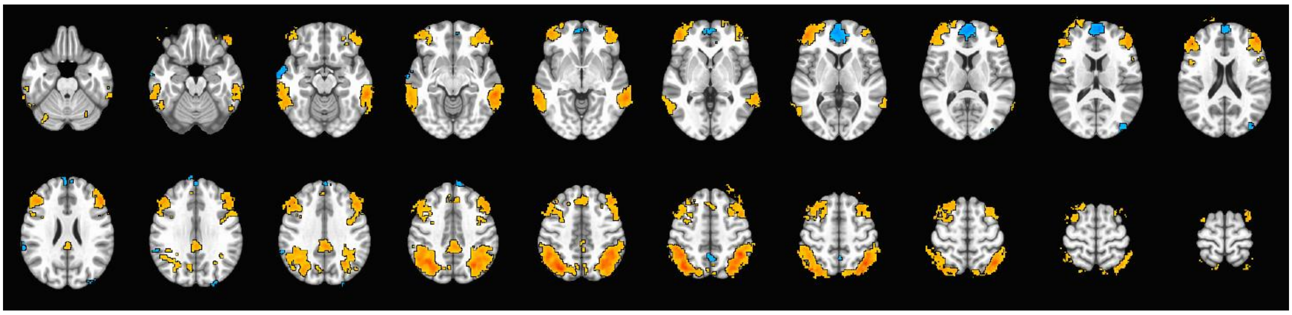

Figure S7.124

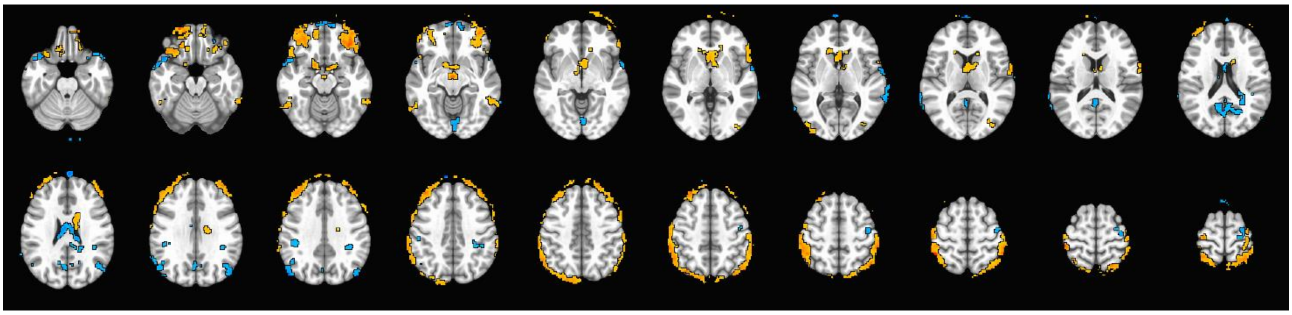

Figure S7.125

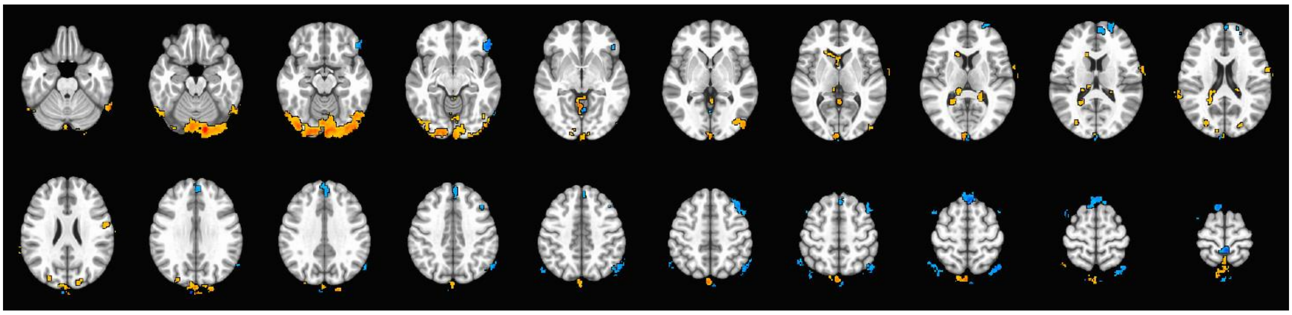

Figure S7.126

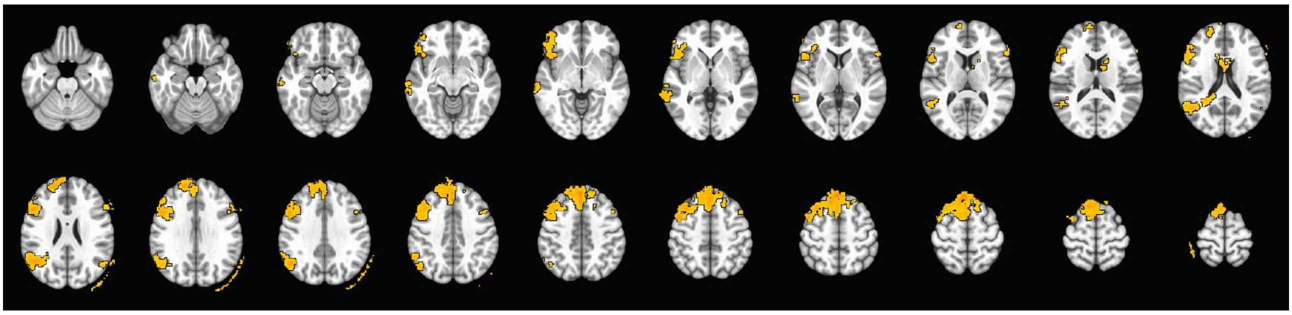

Figure S7.127

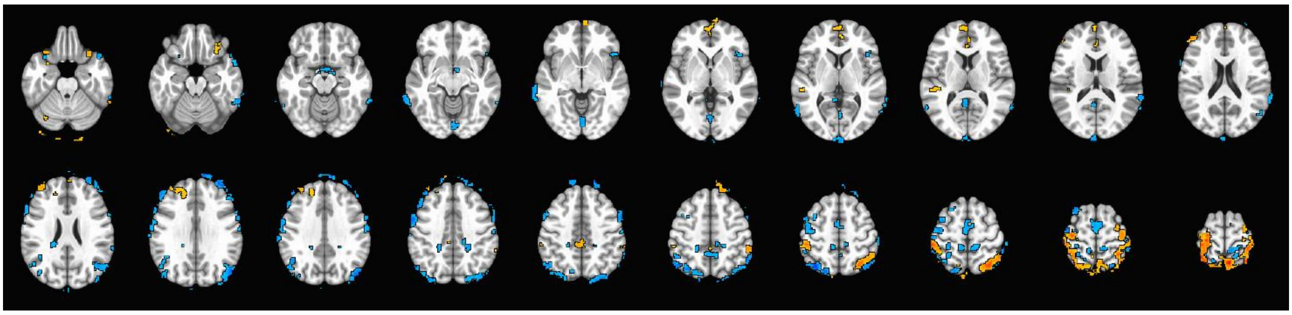

Figure S7.128

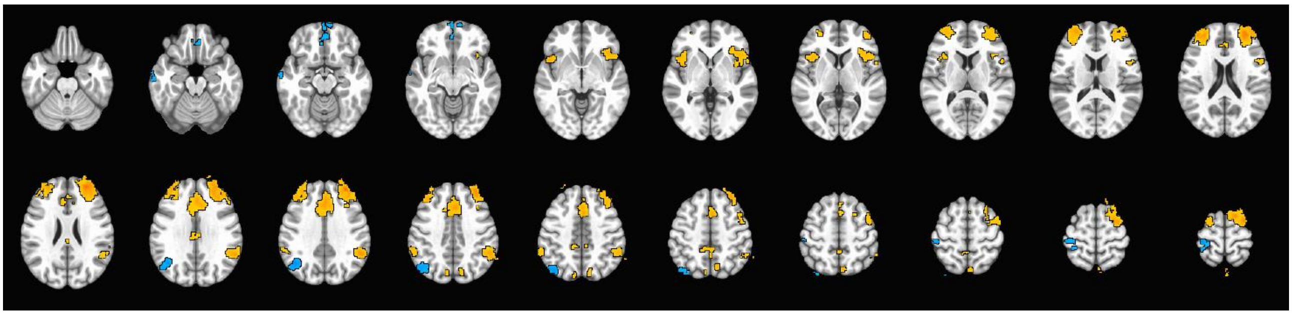

Figure S7.129

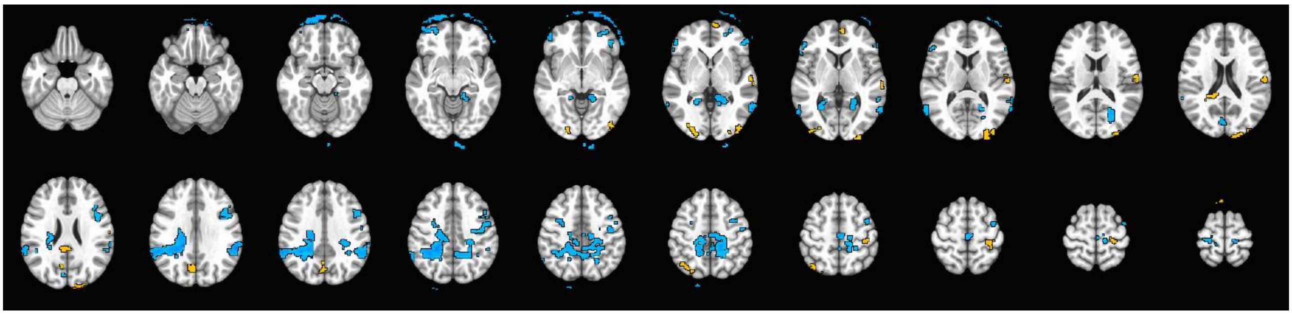

Figure S7.130

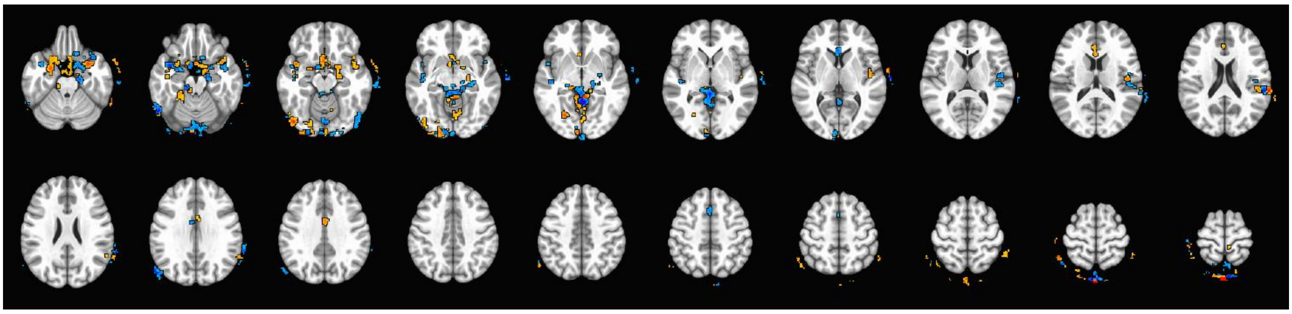

Figure S7.131

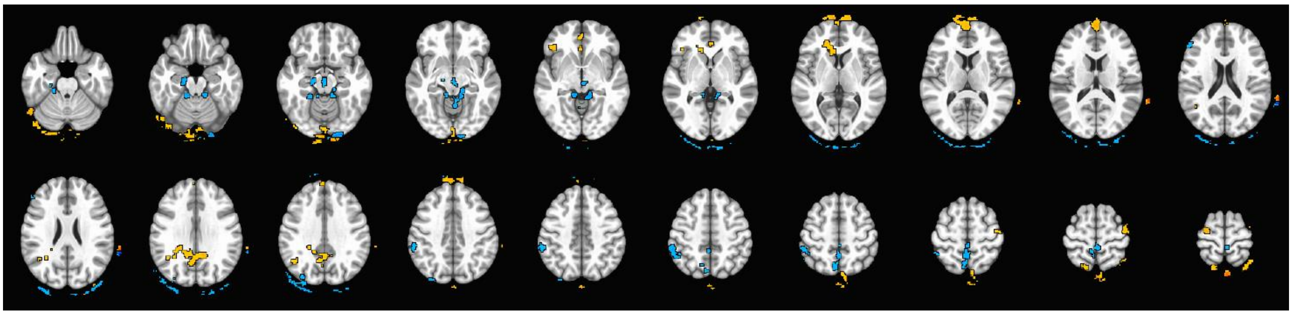

Figure S7.132

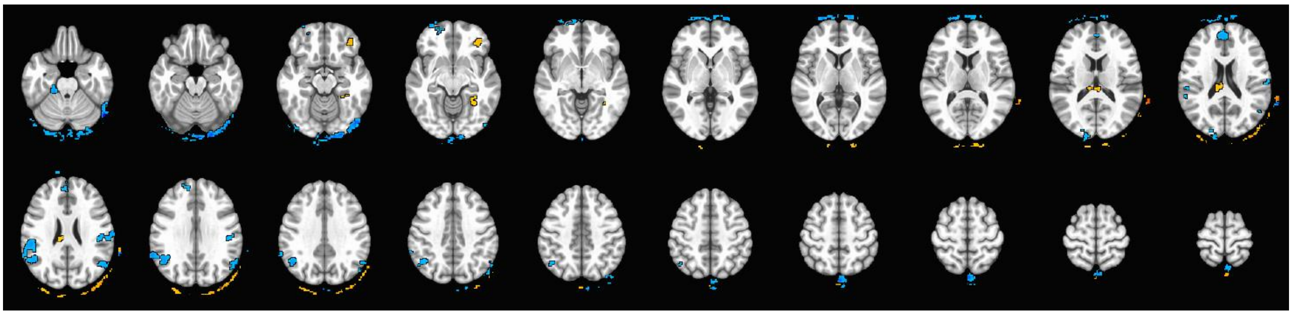

Figure S7.133

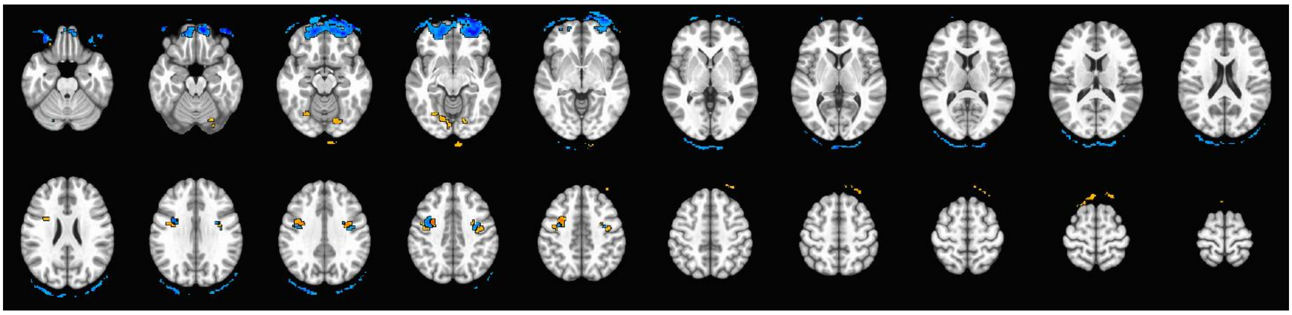

Figure S7.134

Group ICA at 3T with 4mm smoothing and Dimensionality 20

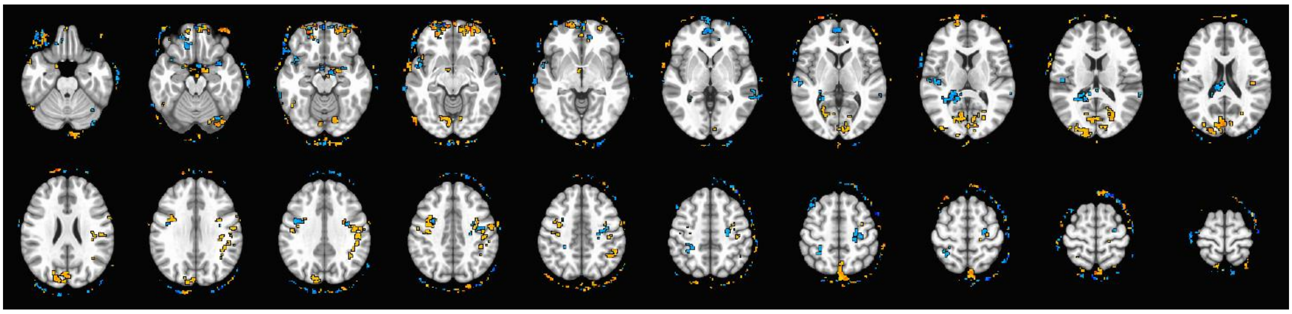

Figure S8.1

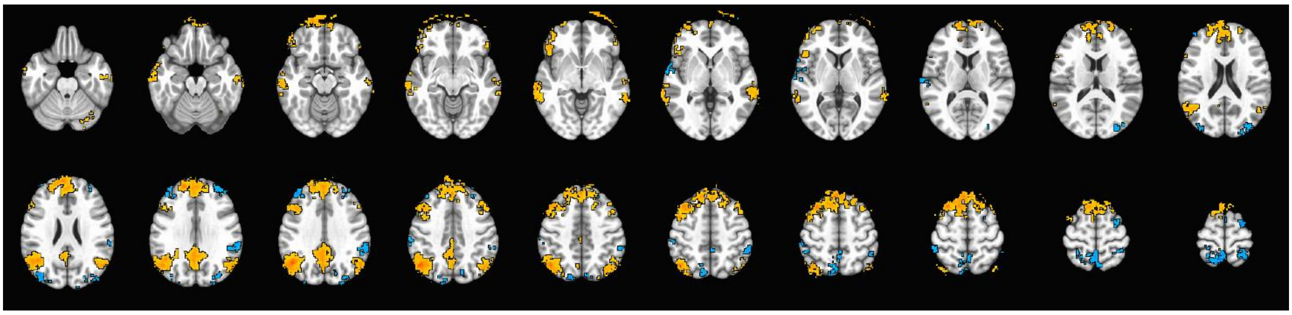

Figure S8.135

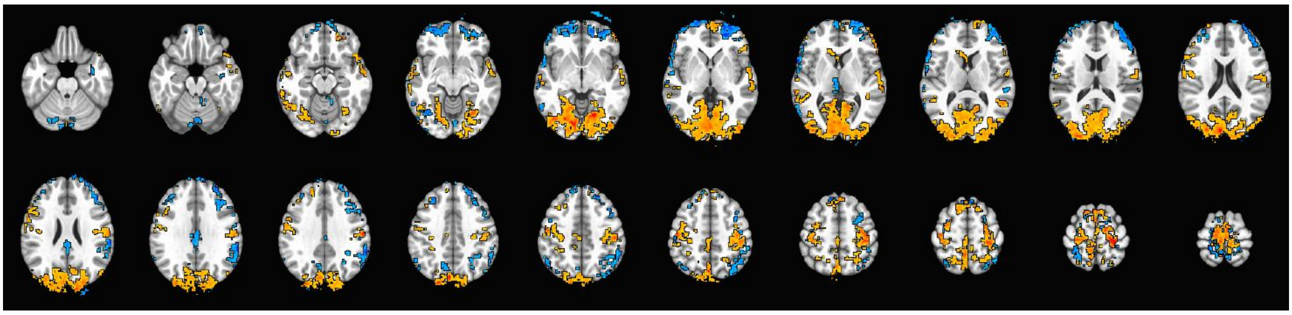

Figure S8.136

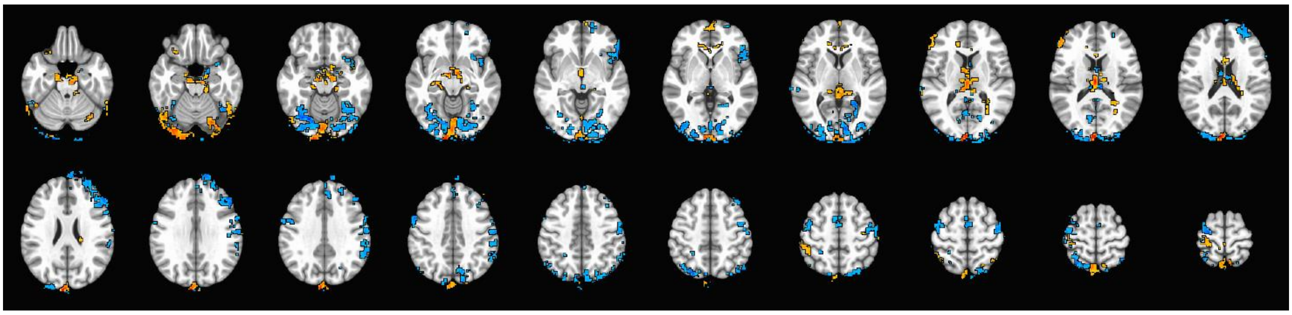

Figure S8.137

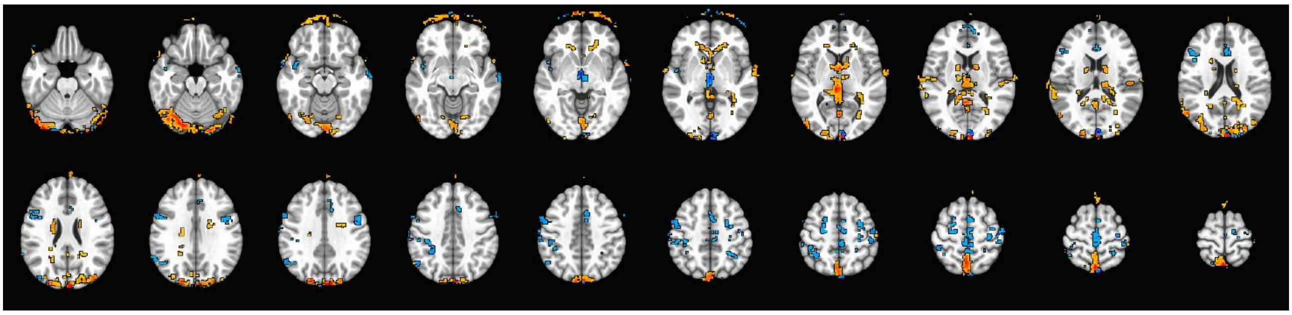

Figure S8.138

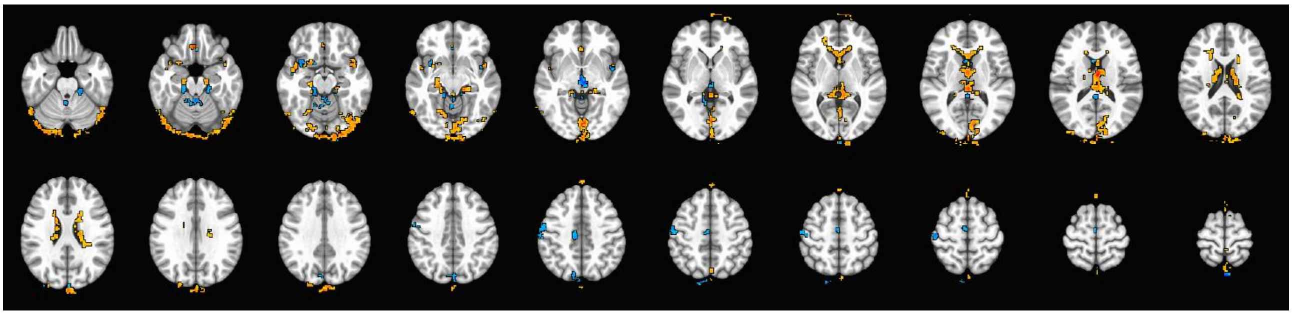

Figure S8.139

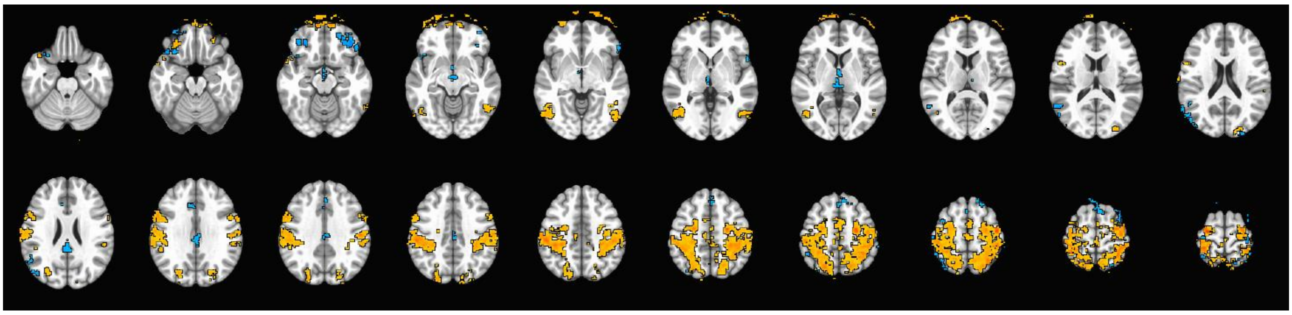

Figure S8.140

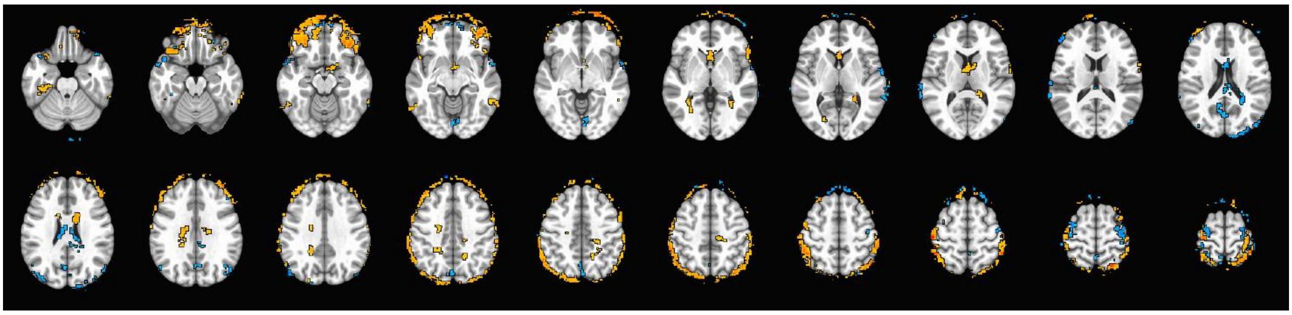

Figure S8.141

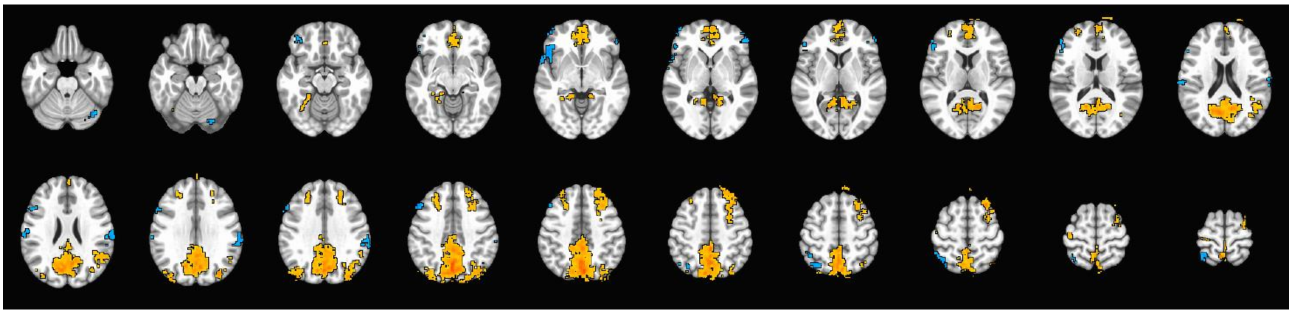
Figure S8.142

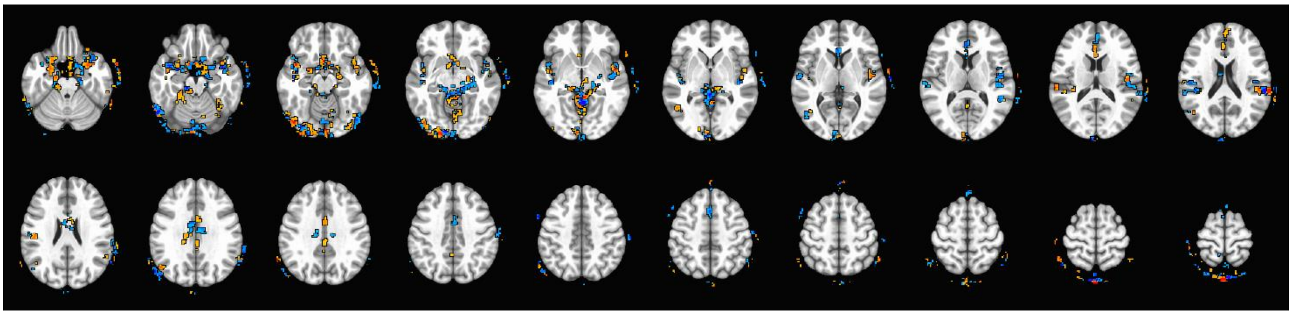
Figure S8.143

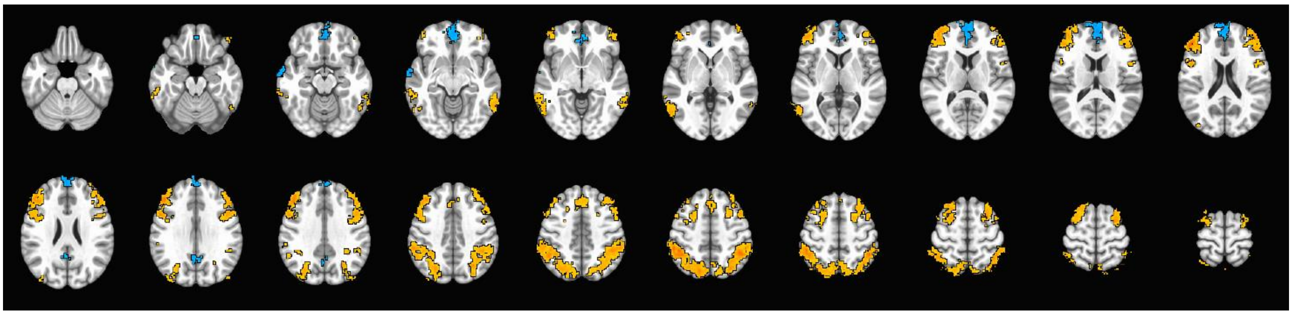
Figure S8.144

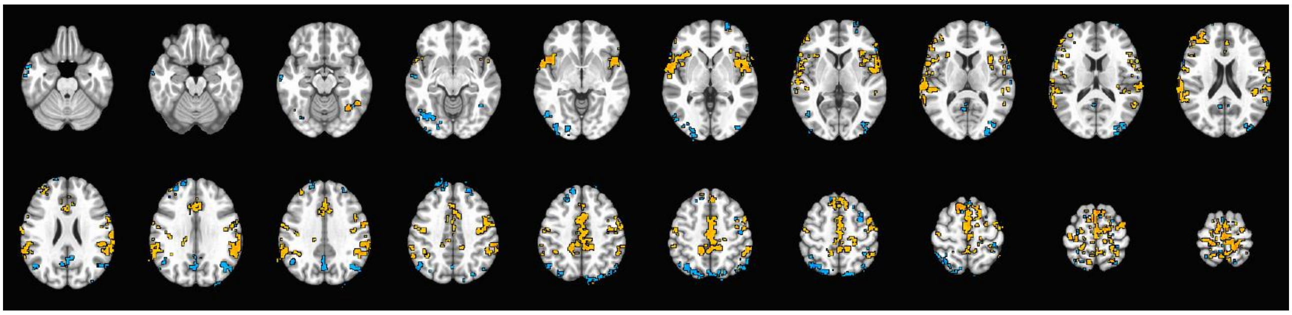
Figure S8.145

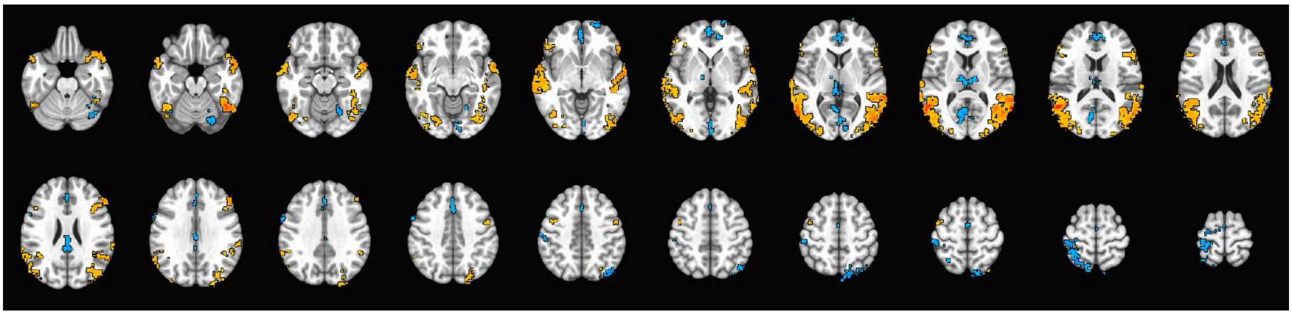

Figure S8.146

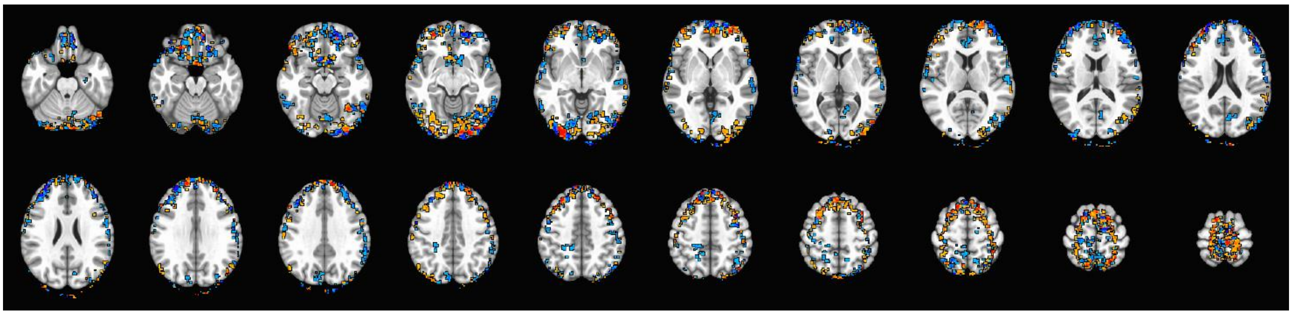

Figure S8.147

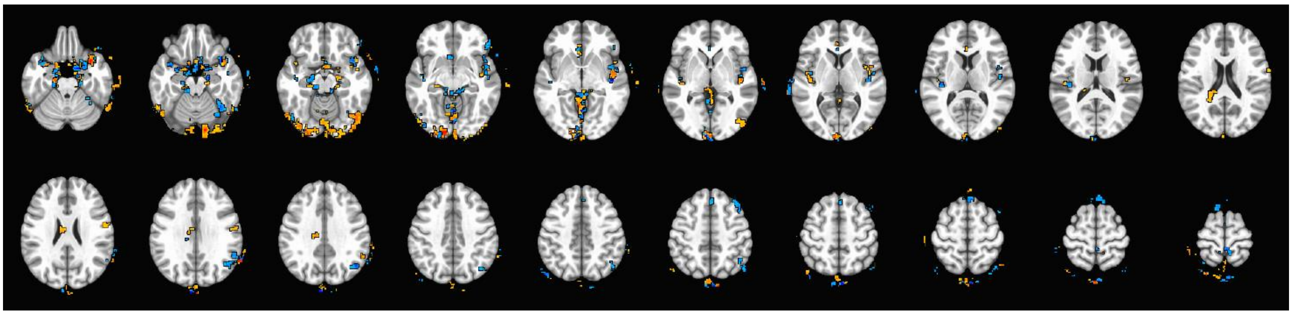

Figure S8.148

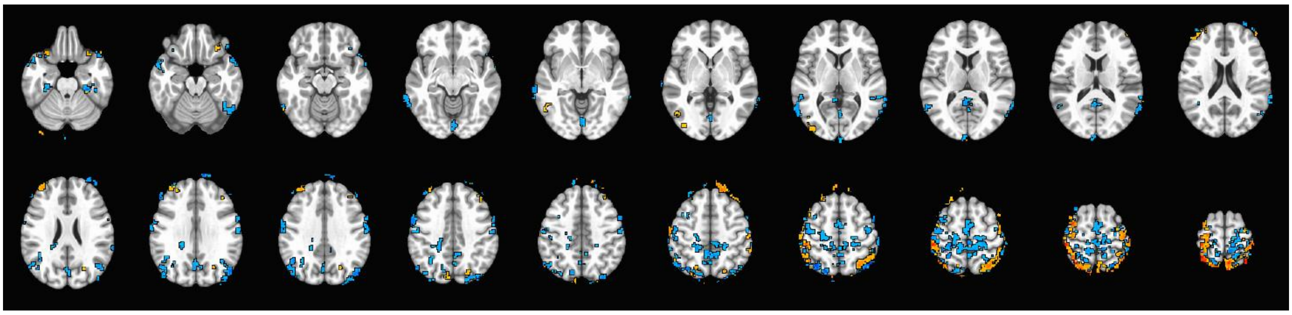

Figure S8.149

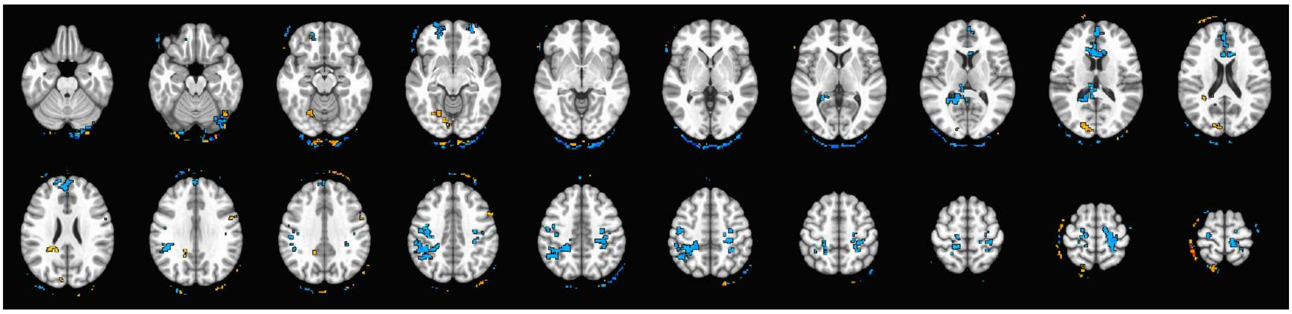

Figure S8.150

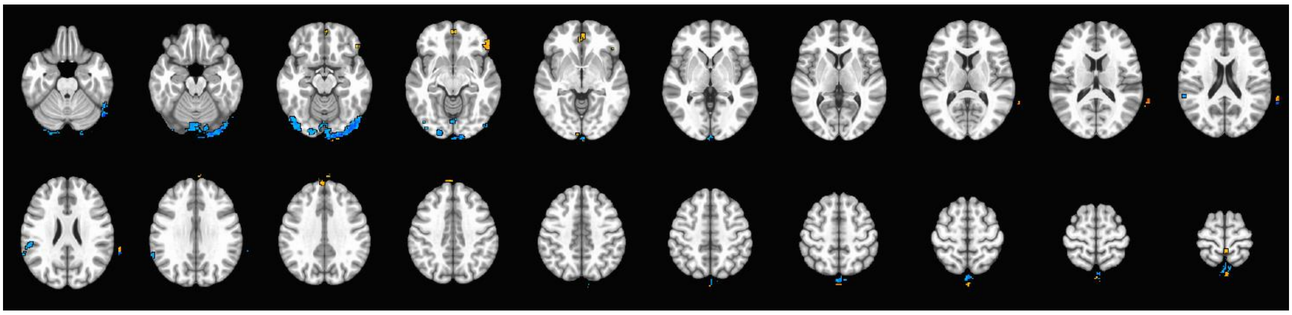

Figure S8.151

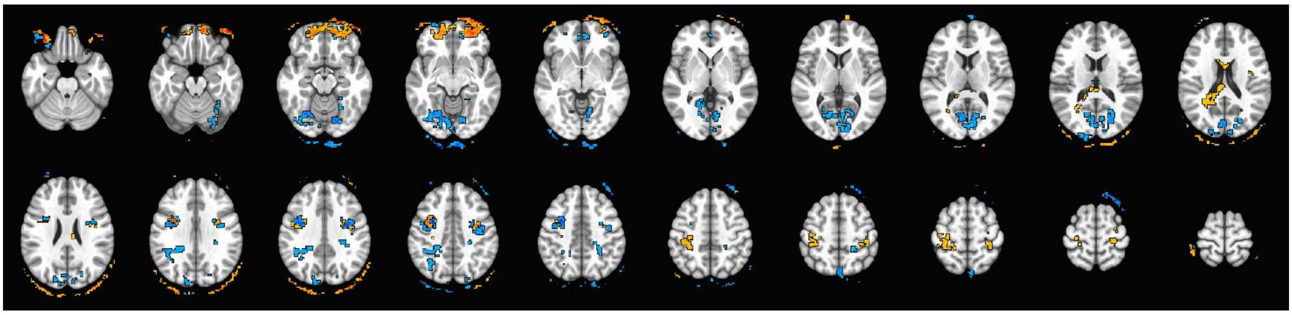

Figure S8.152

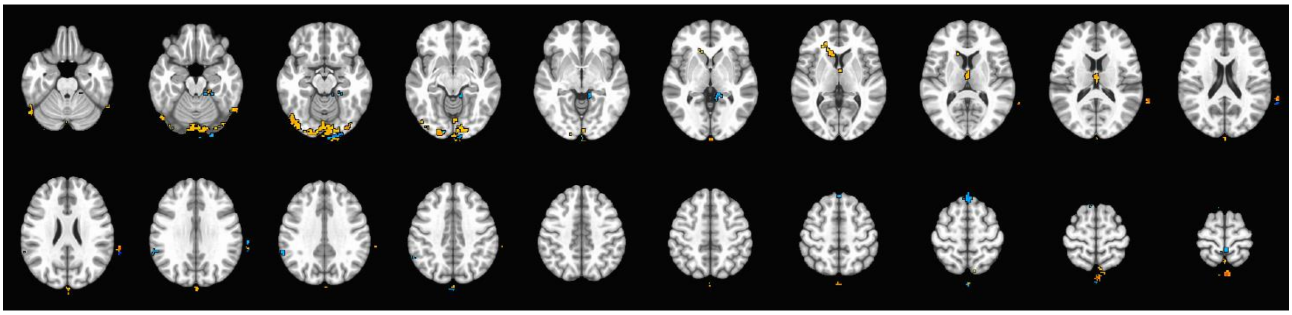

Figure S8.153

Group ICA at 7T with 12mm smoothing and Dimensionality 20

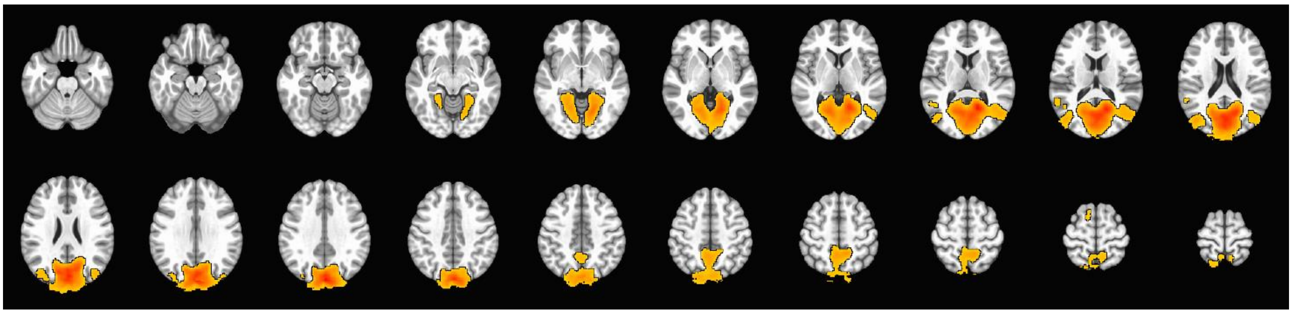

Figure S9.1

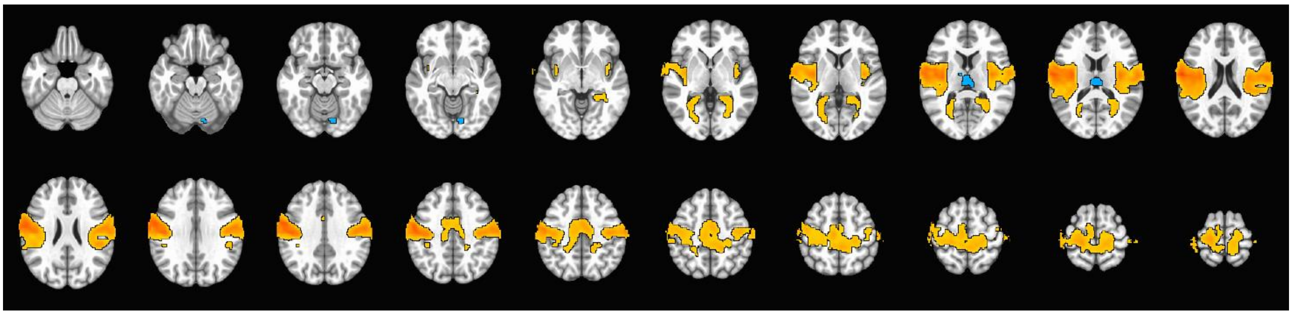

Figure S9.154

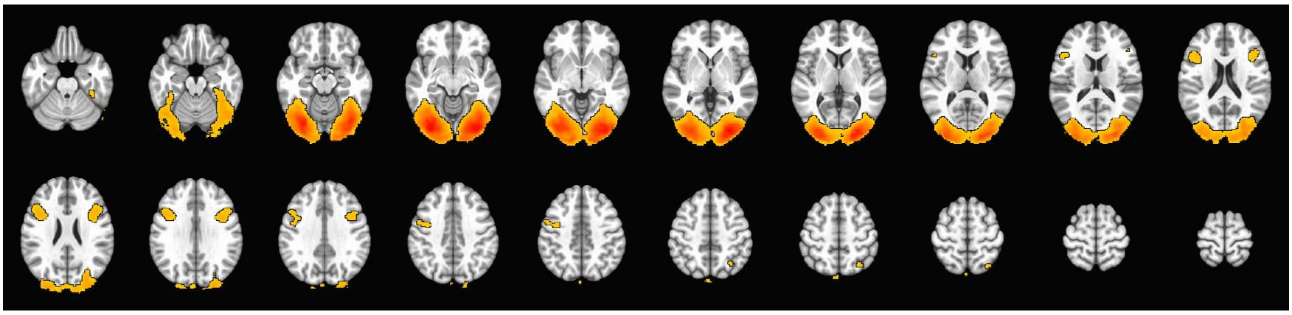

Figure S9.155

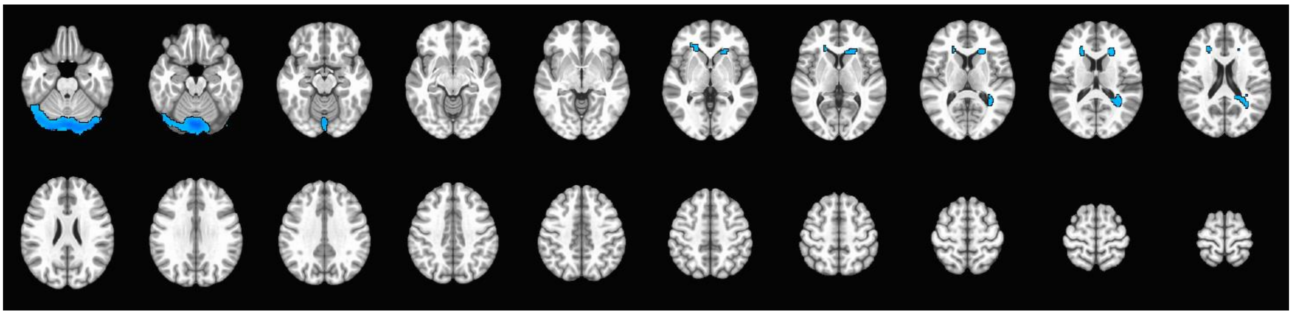

Figure S9.156

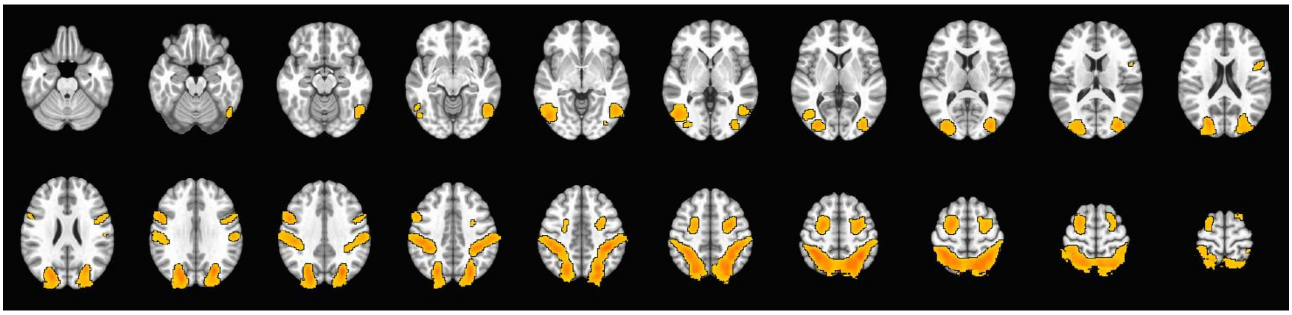

Figure S9.157

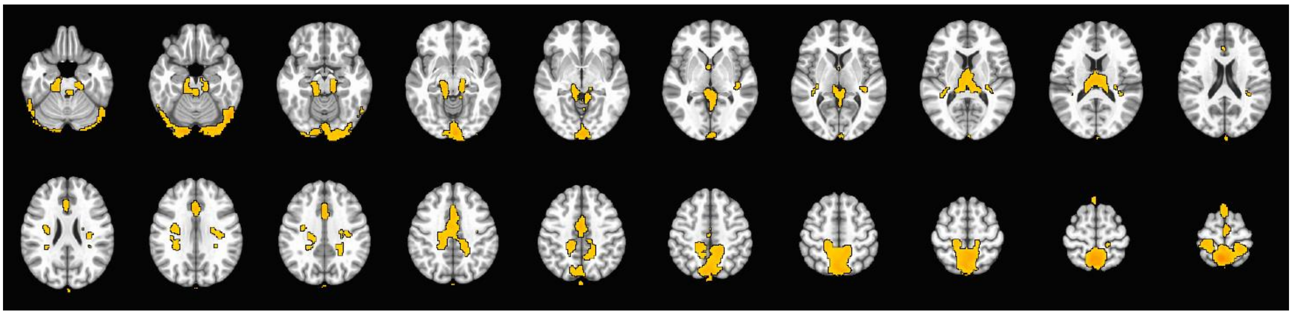

Figure S9.158

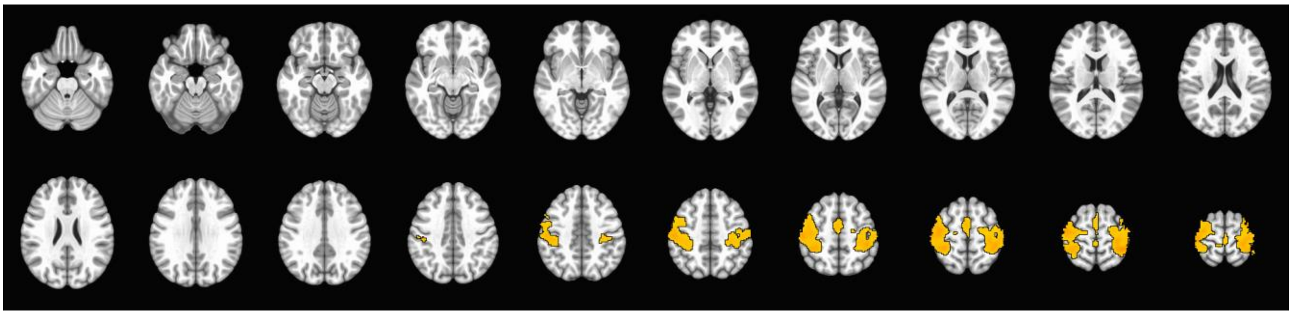

Figure S9.159

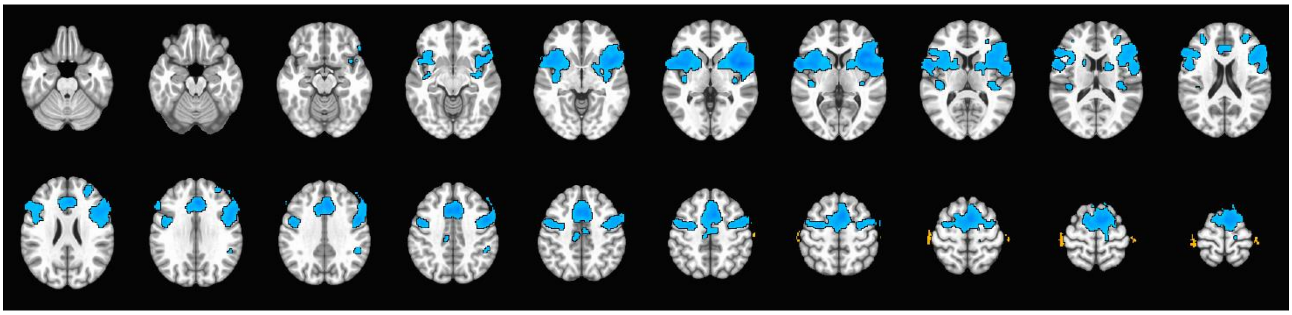

Figure S9.160

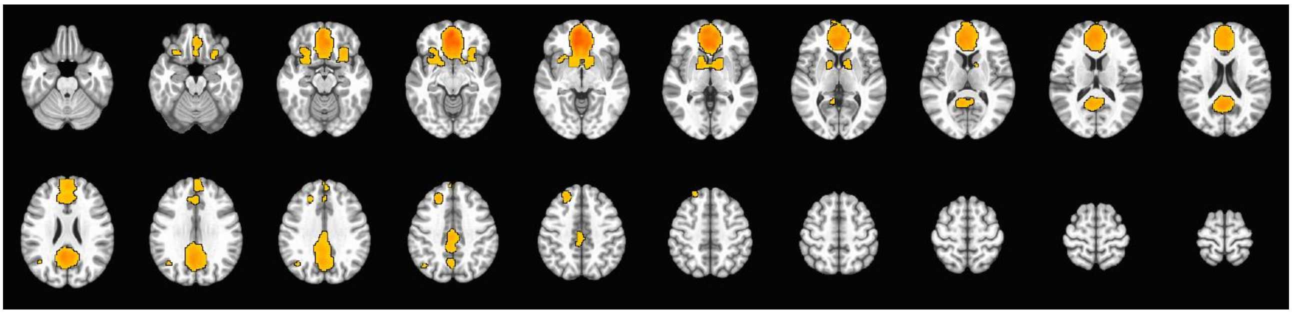

Figure S9.161

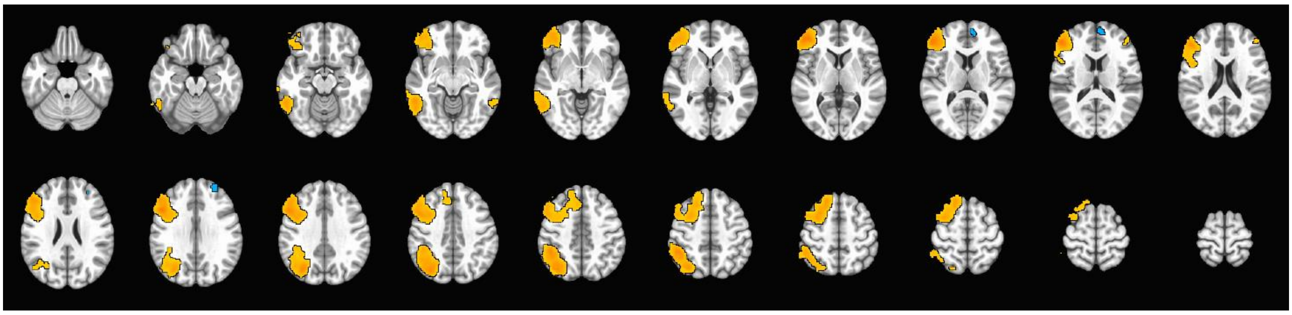

Figure S9.162

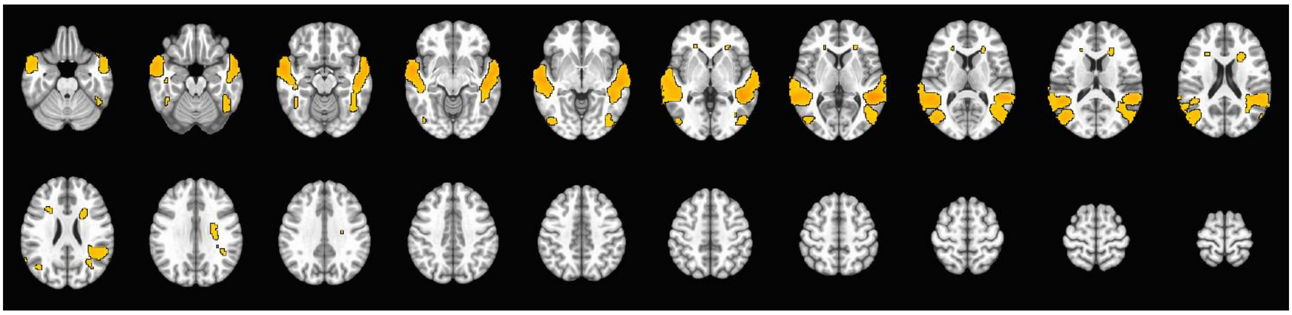

Figure S9.163

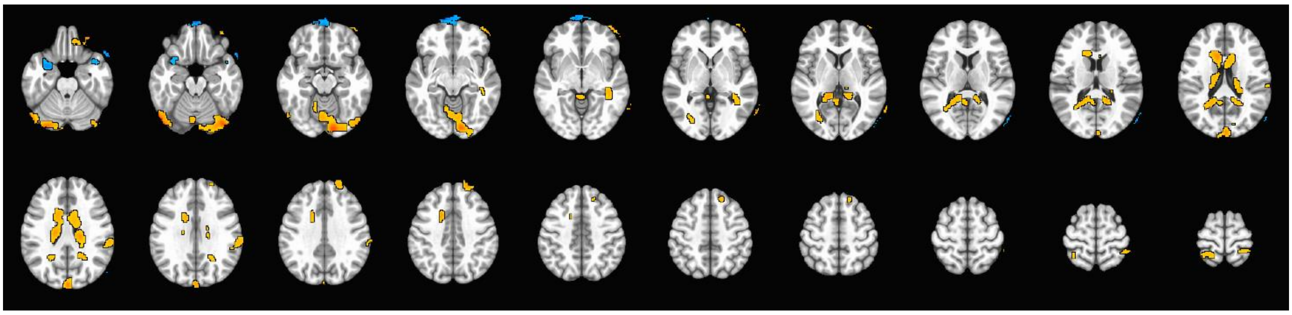

Figure S9.164

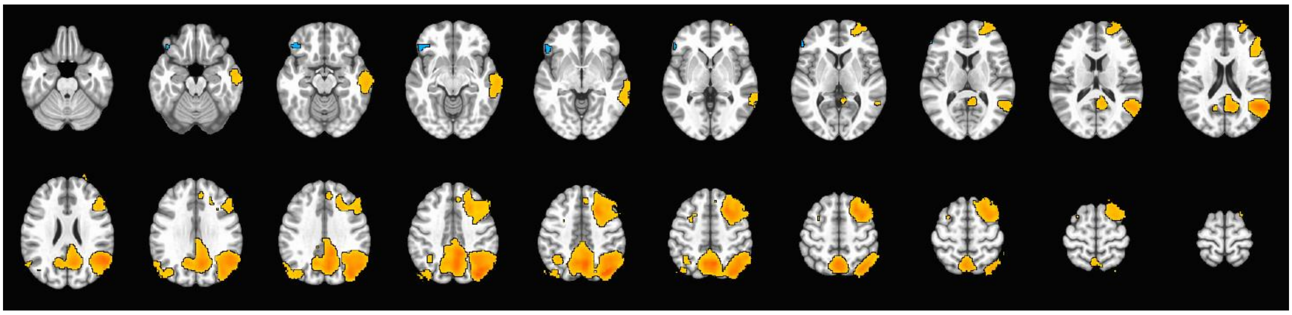

Figure S9.165

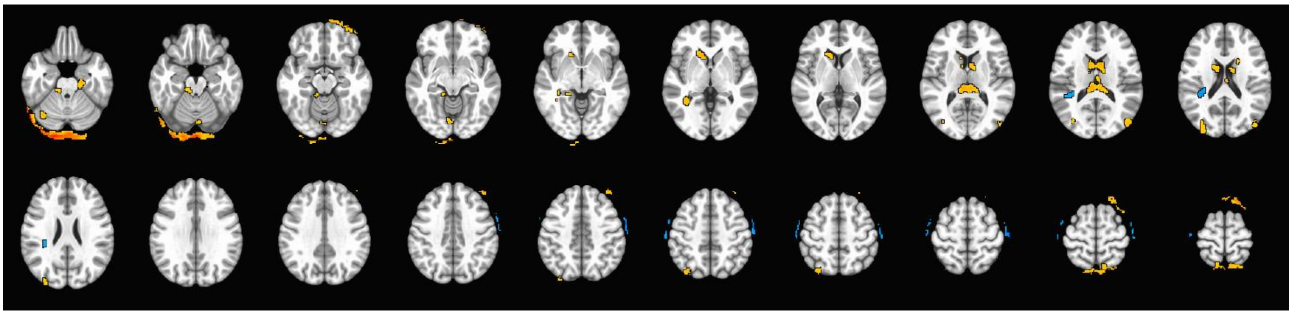

Figure S9.166

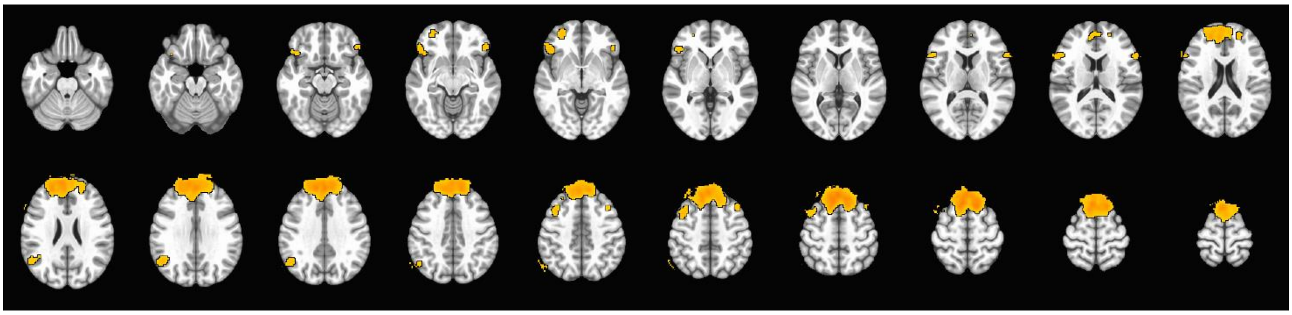

Figure S9.167

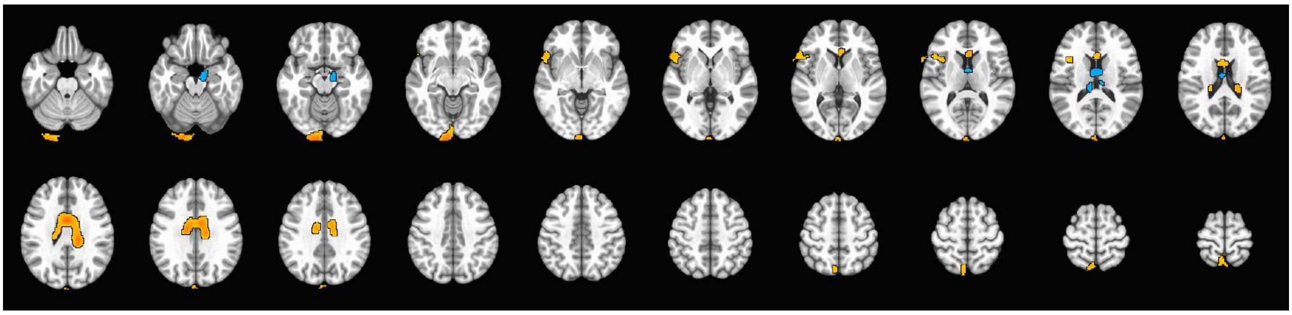

Figure S9.168

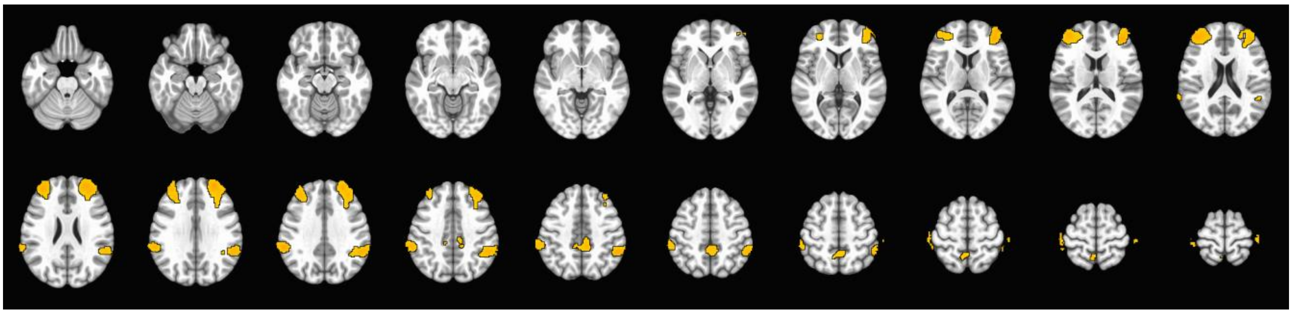

Figure S9.169

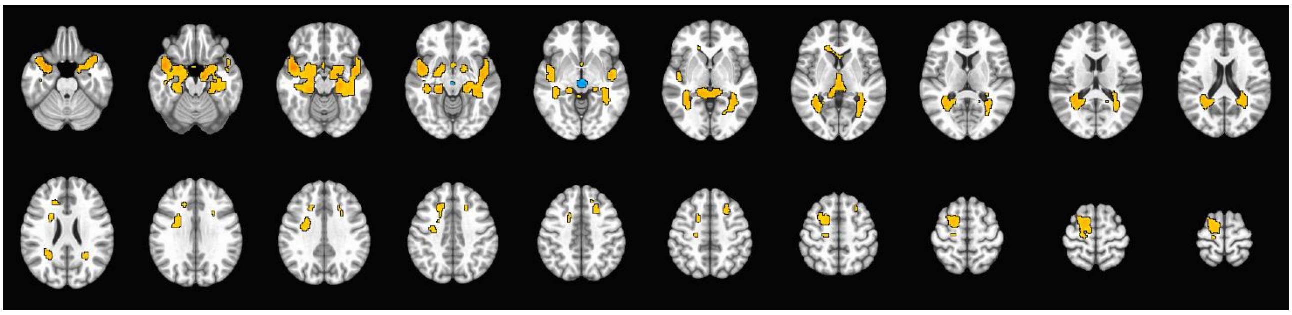

Figure S9.170

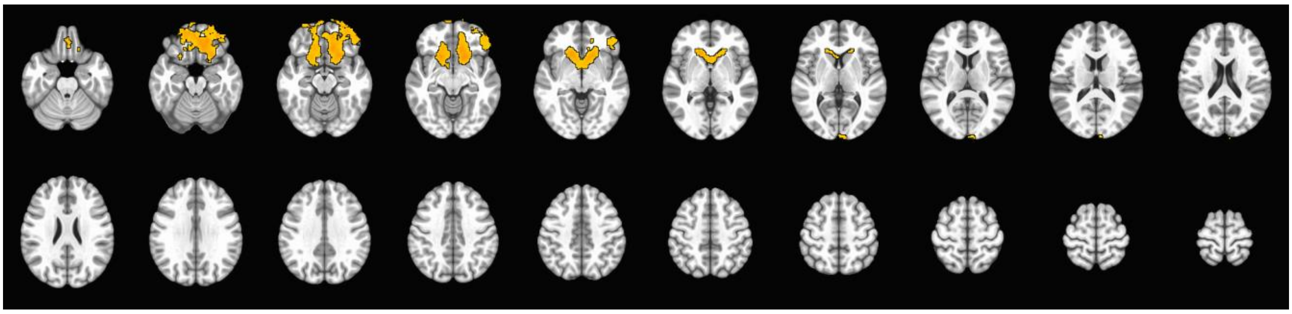

Figure S9.171

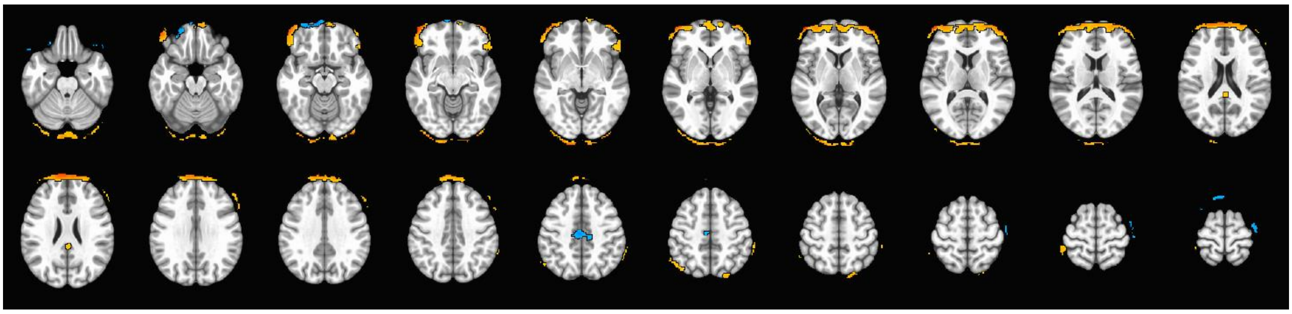

Figure S9.172

Group ICA at 7T with 8mm smoothing and Dimensionality 20

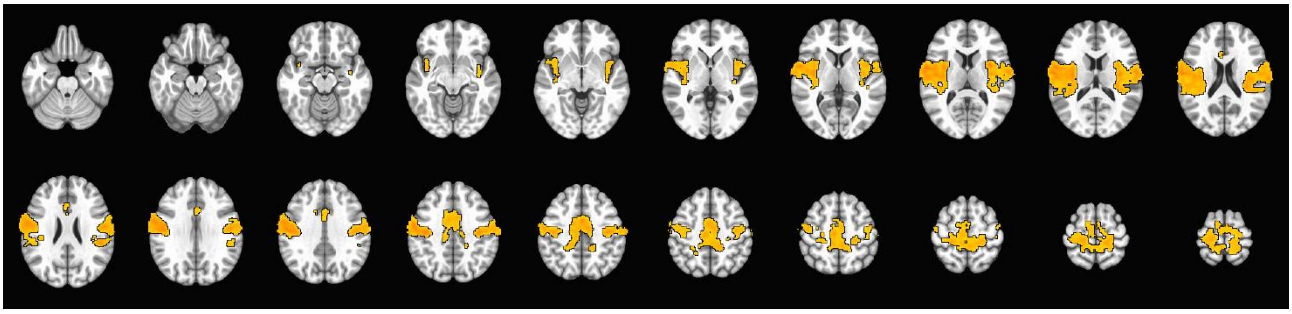

Figure S10.1

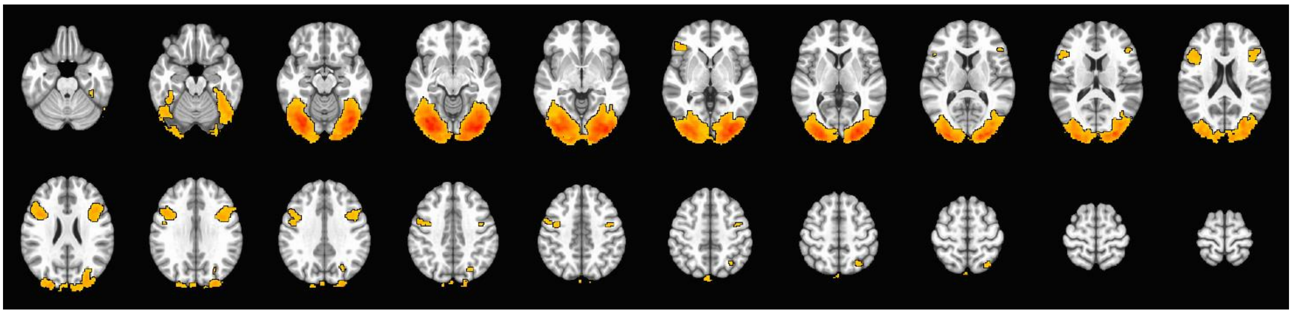

Figure S10.173

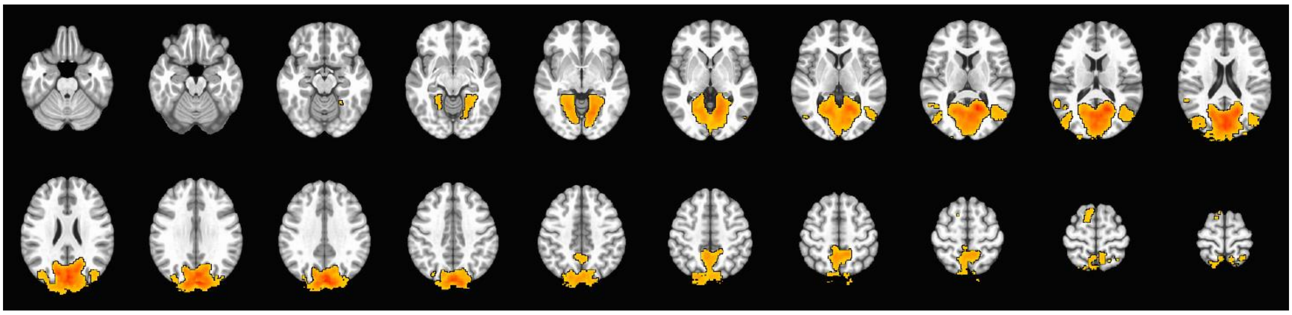

Figure S10.174

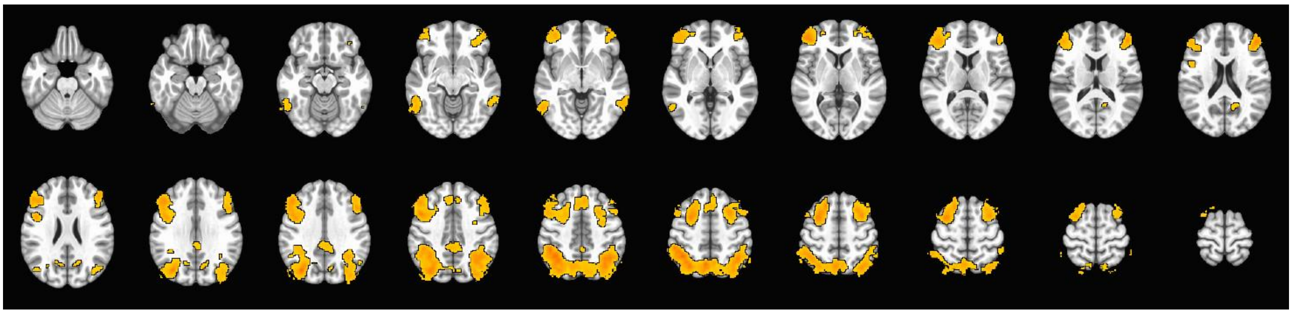

Figure S10.175

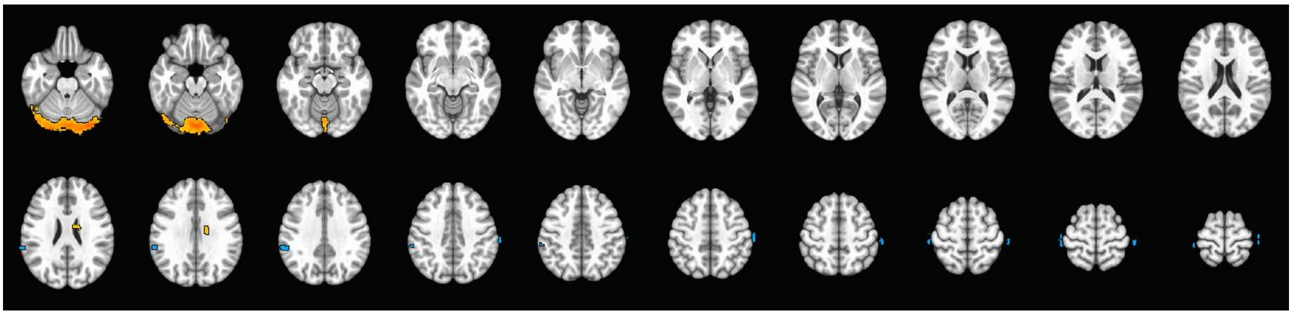

Figure S10.176

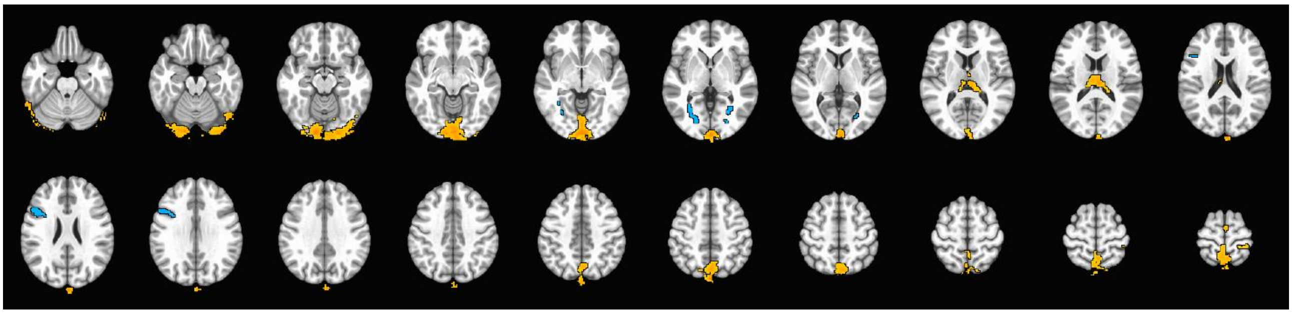

Figure S10.177

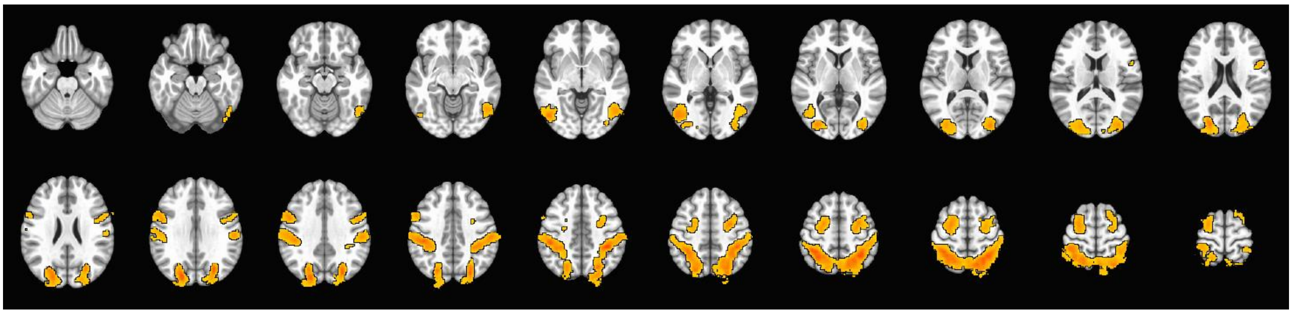

Figure S10.178

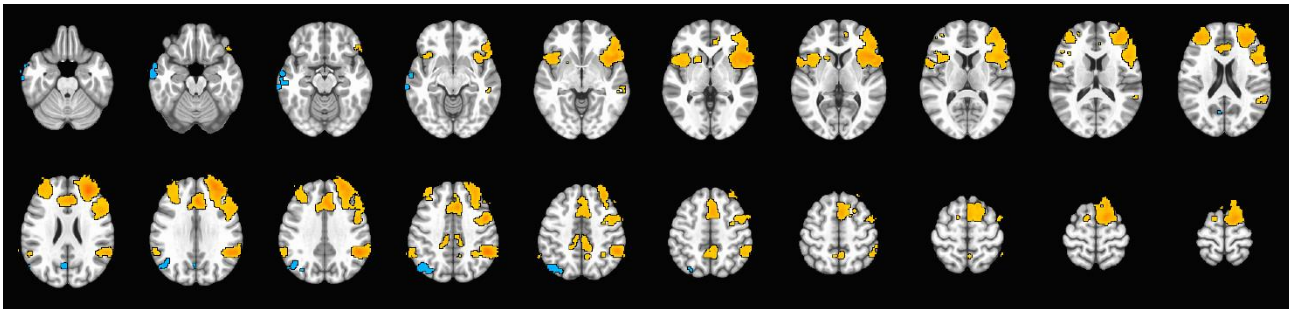

Figure S10.179

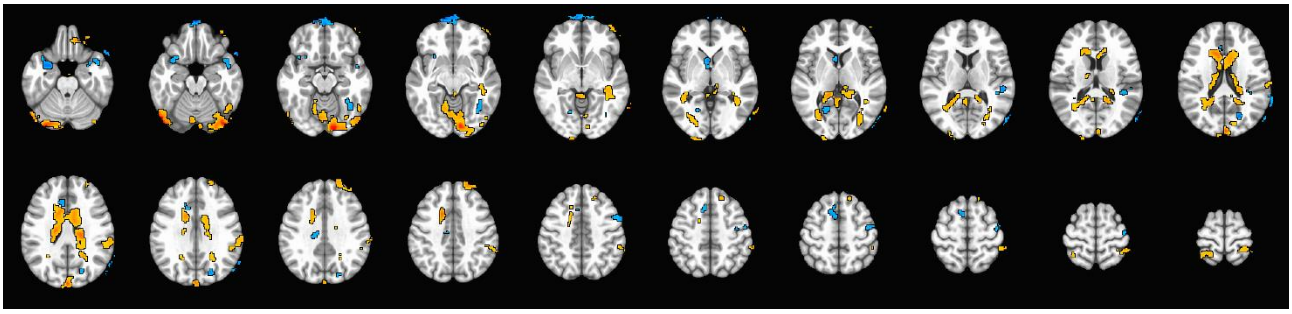

Figure S10.180

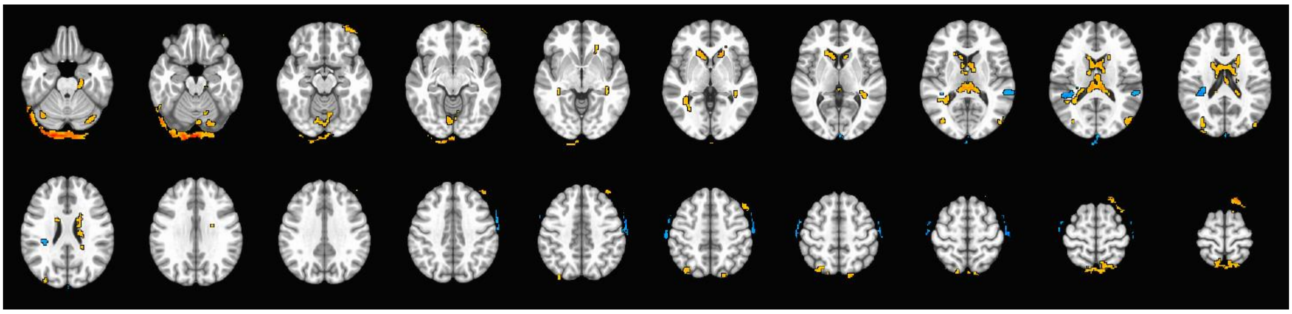

Figure S10.181

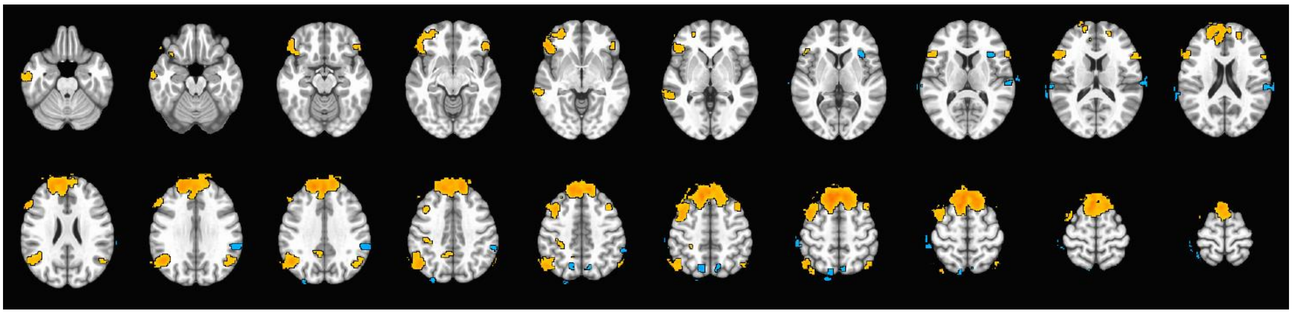

Figure S10.182

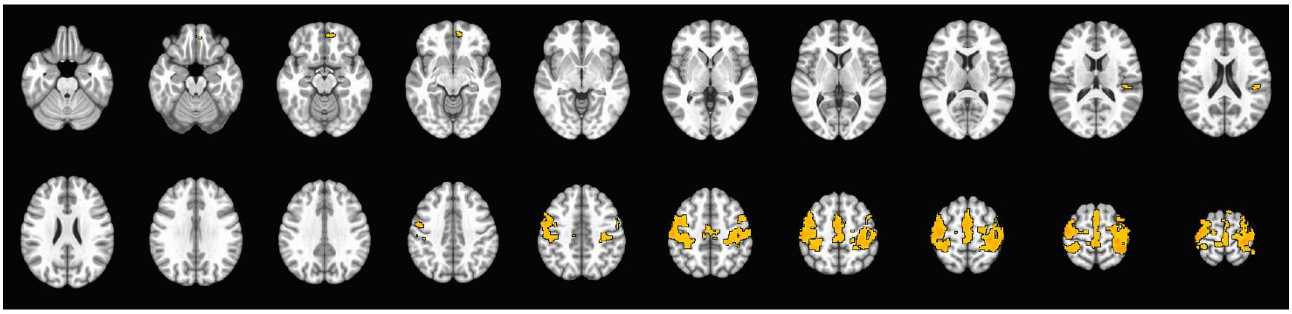

Figure S10.183

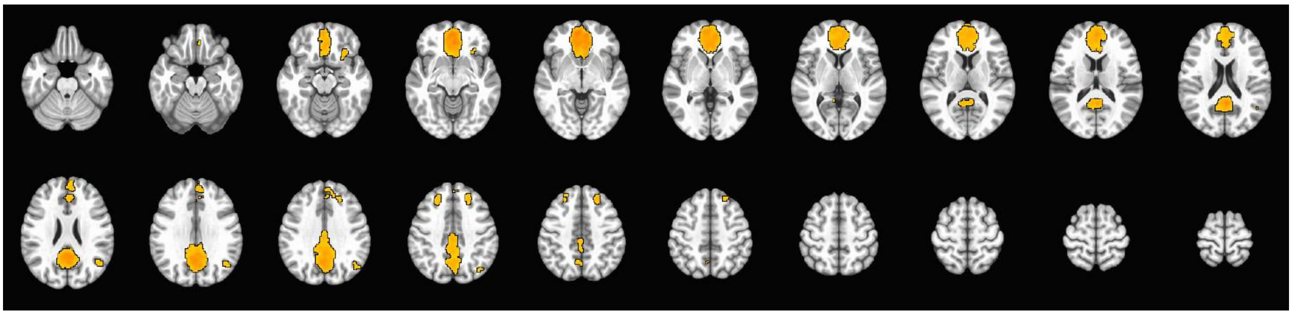

Figure S10.184

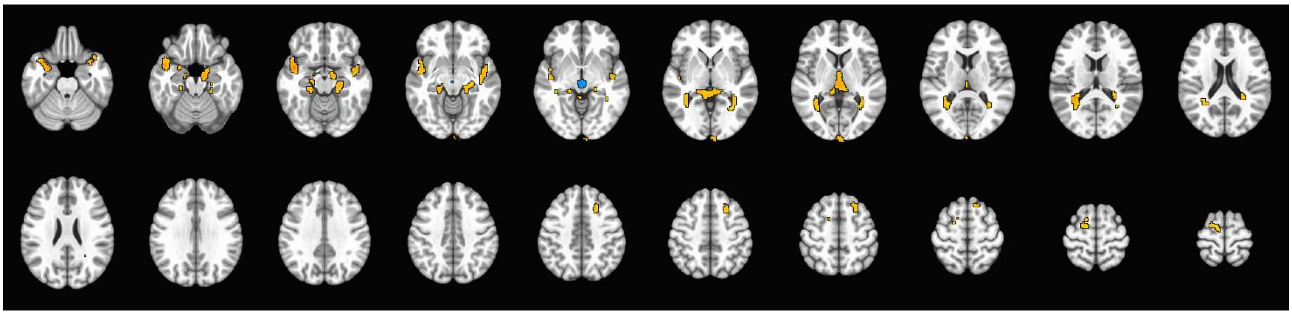

Figure S10.185

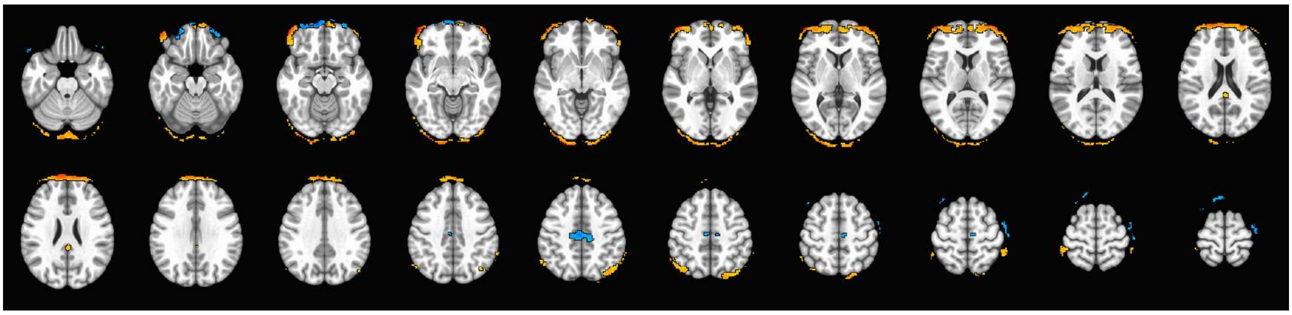

Figure S10.186

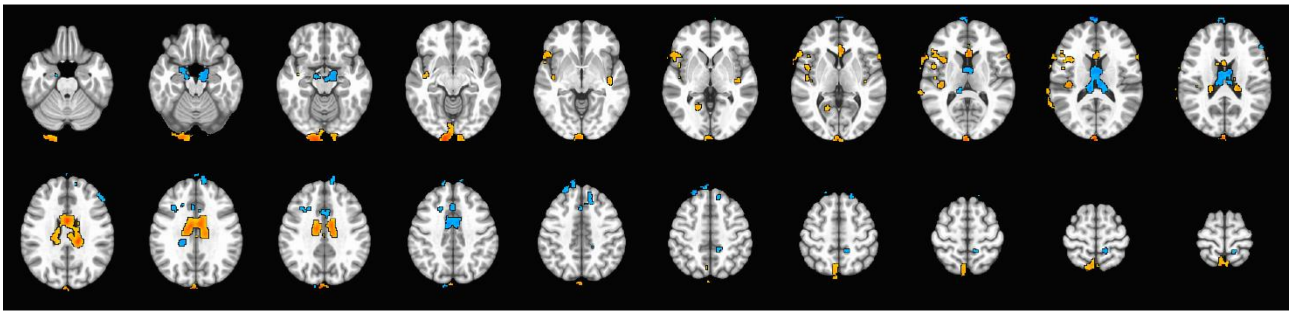

Figure S10.187

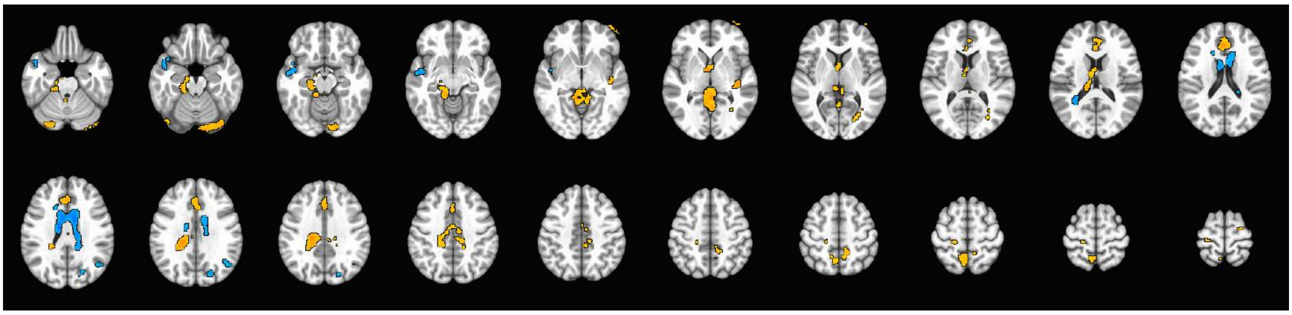

Figure S10.188

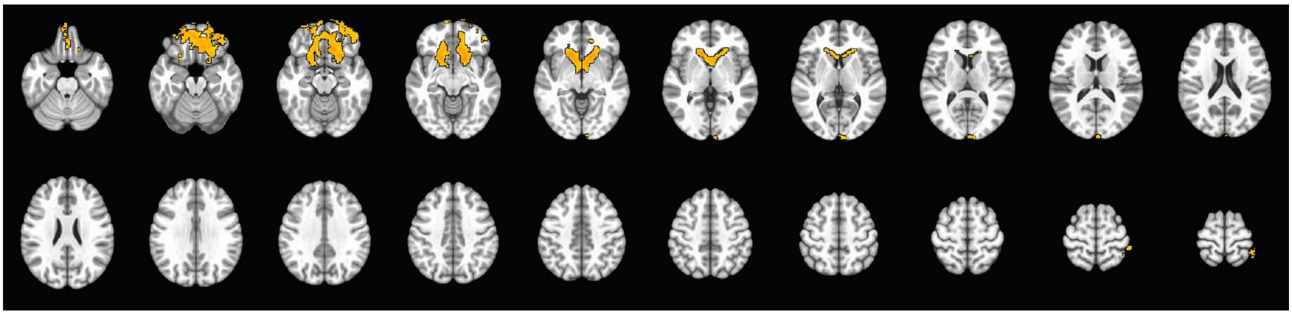

Figure S10.189

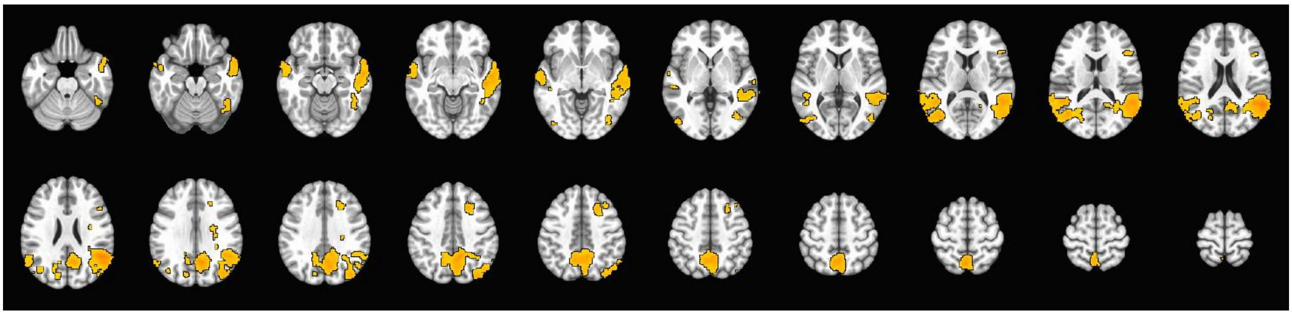

Figure S10.190

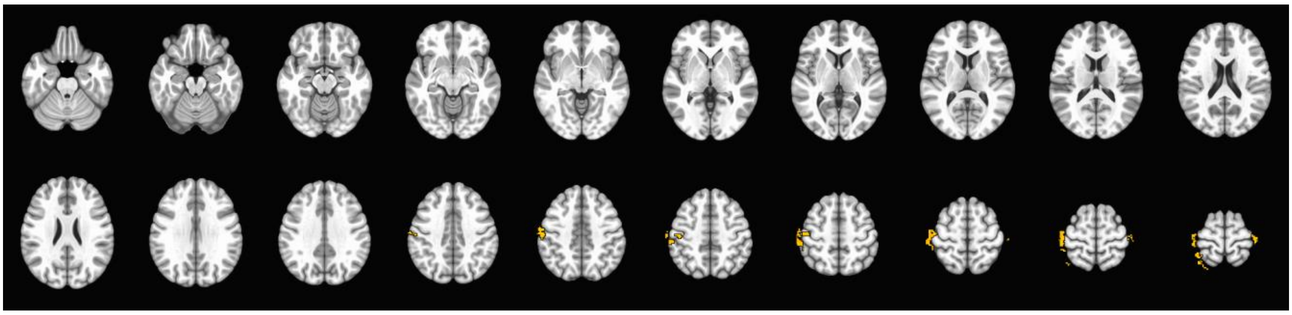

Figure S10.191

Group ICA at 7T with 6mm smoothing and Dimensionality 20

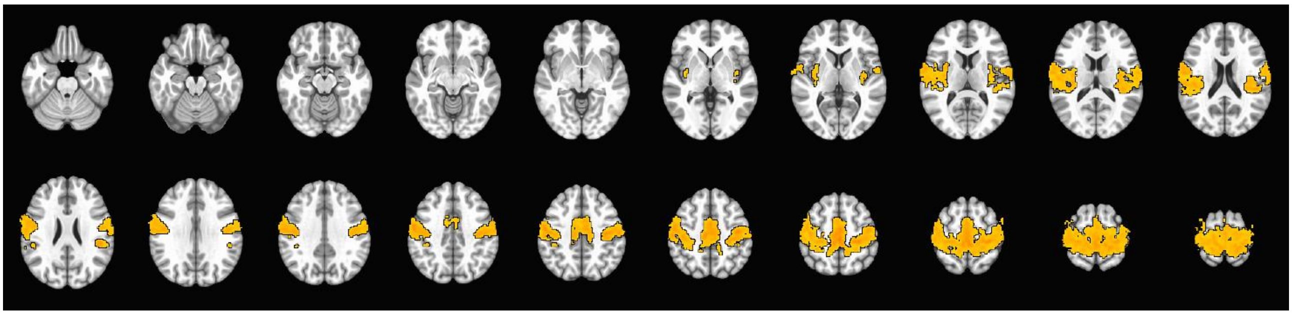

Figure S1192.1

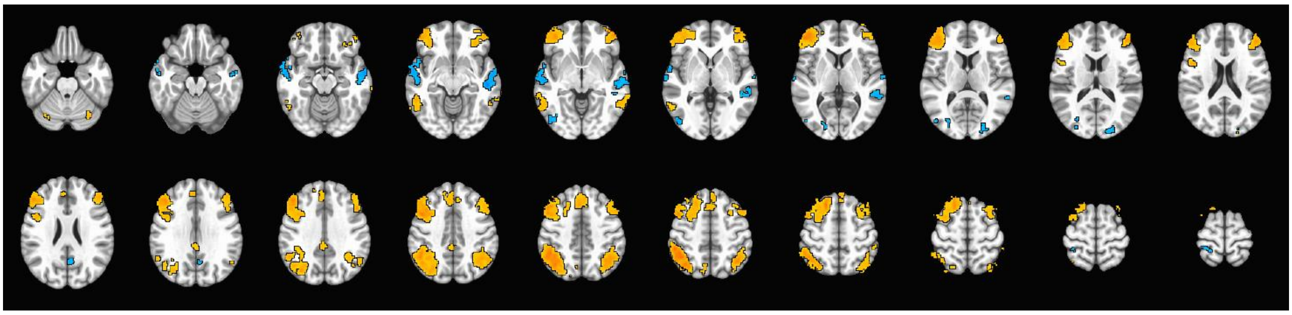

Figure S11.193

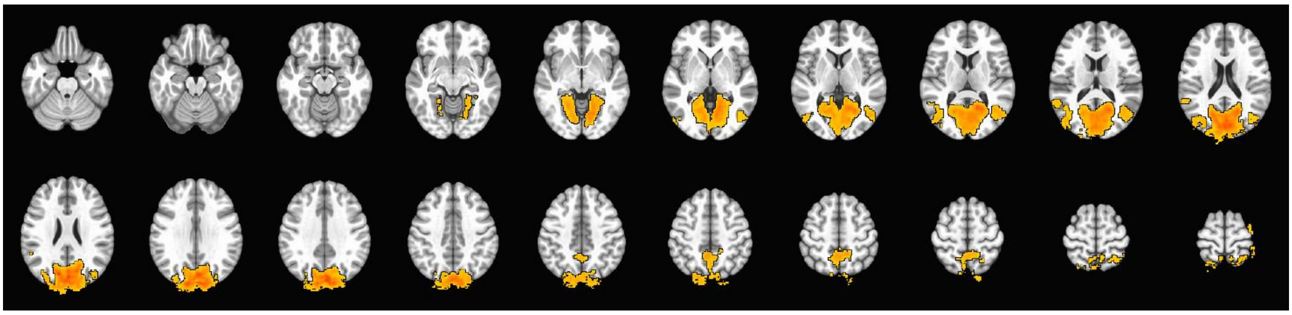

Figure S11.194

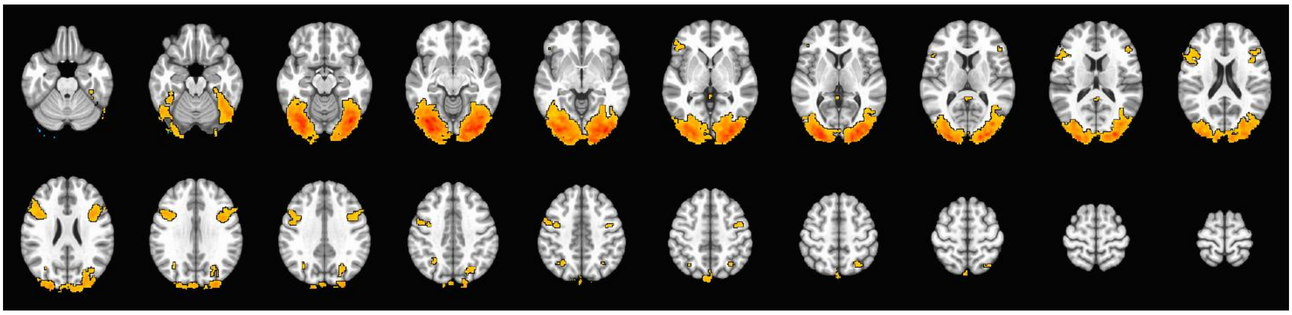

Figure S11.195

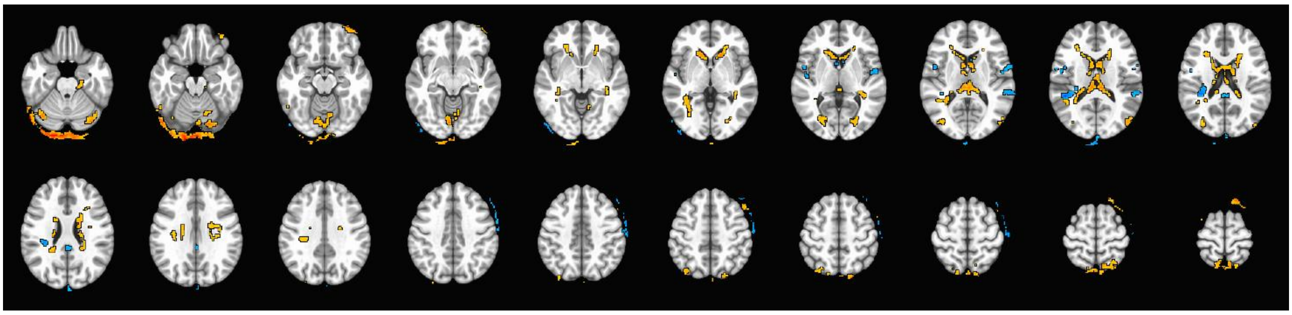

Figure S11.196

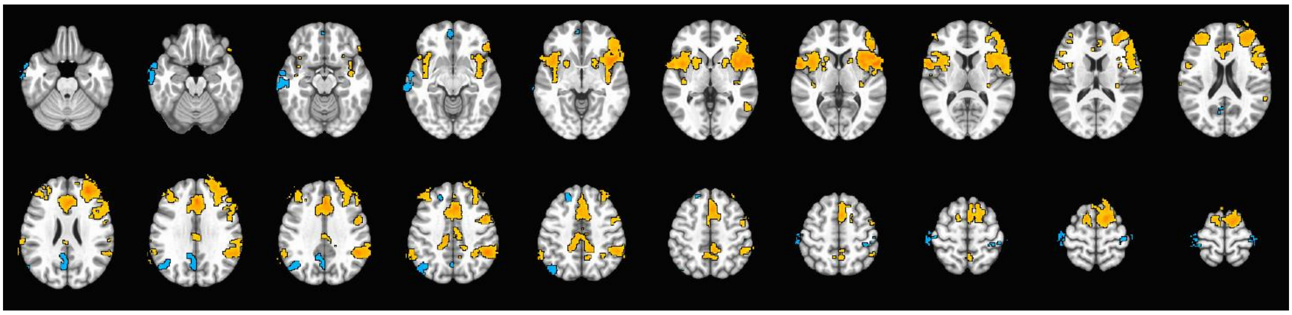

Figure S11.197

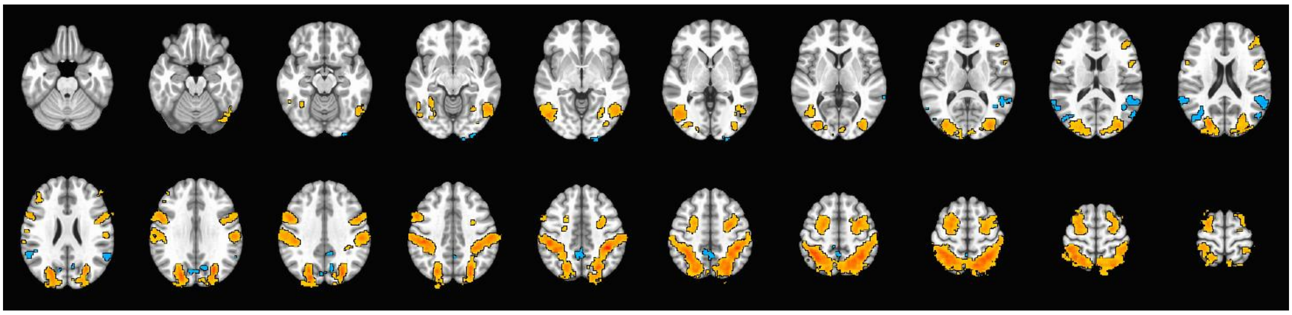

Figure S11.198

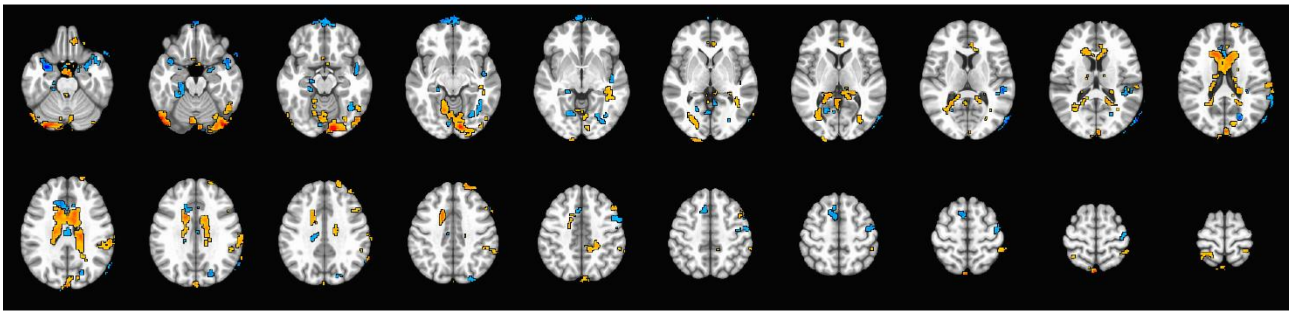

Figure S11.199

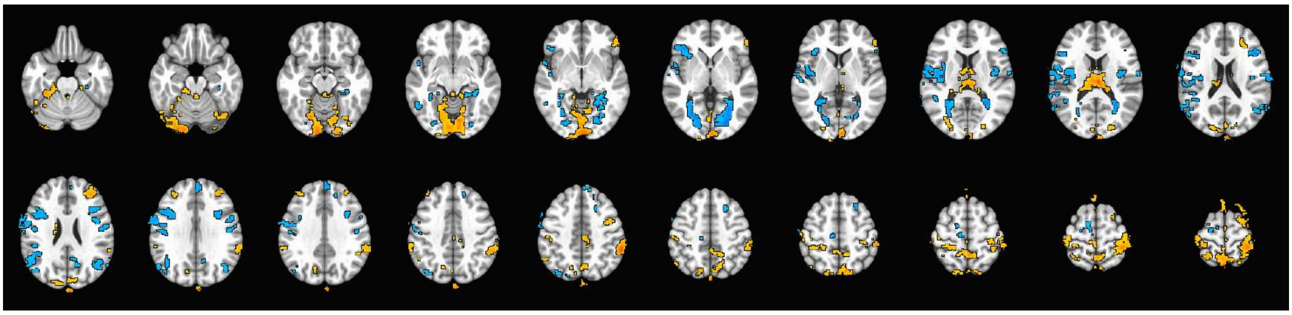

Figure S11.200

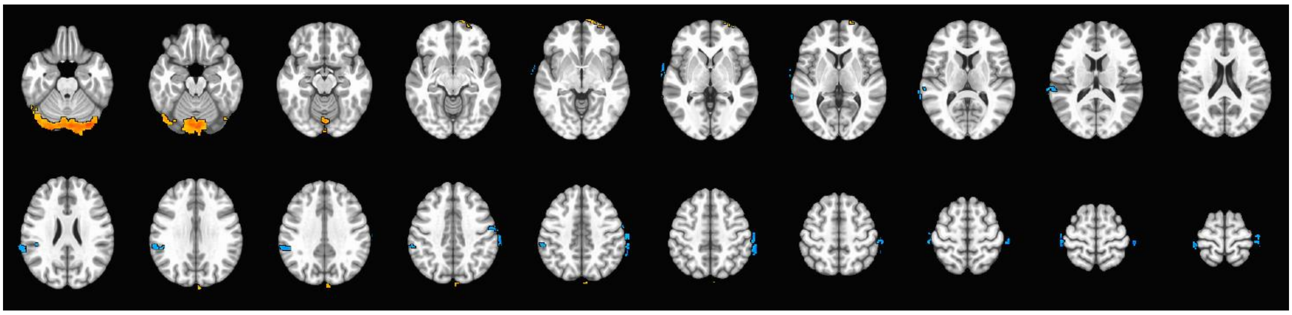

Figure S11.201

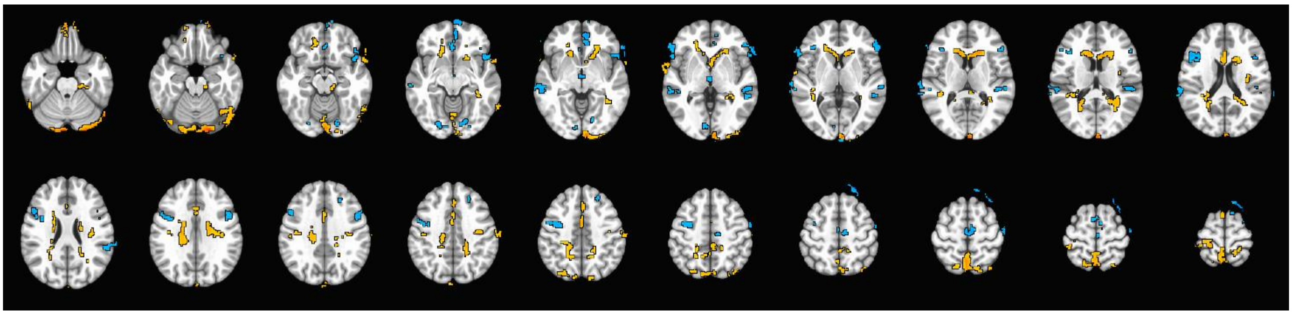

Figure S11.202

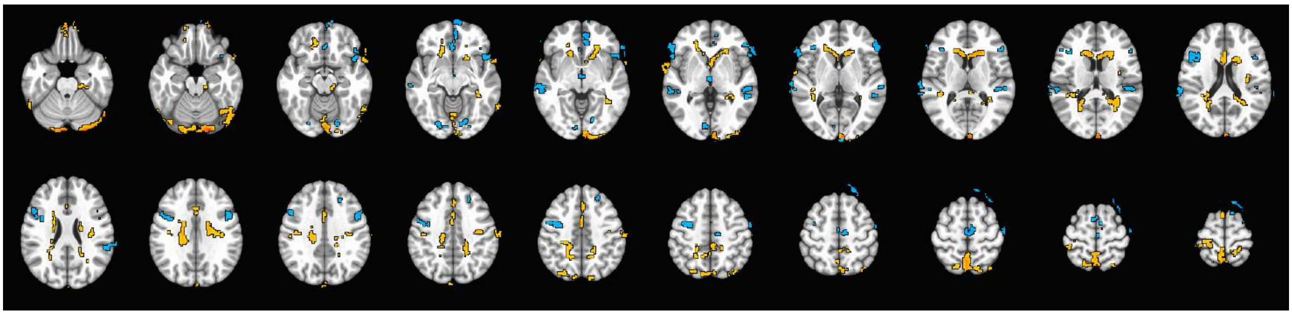

Figure S11.203

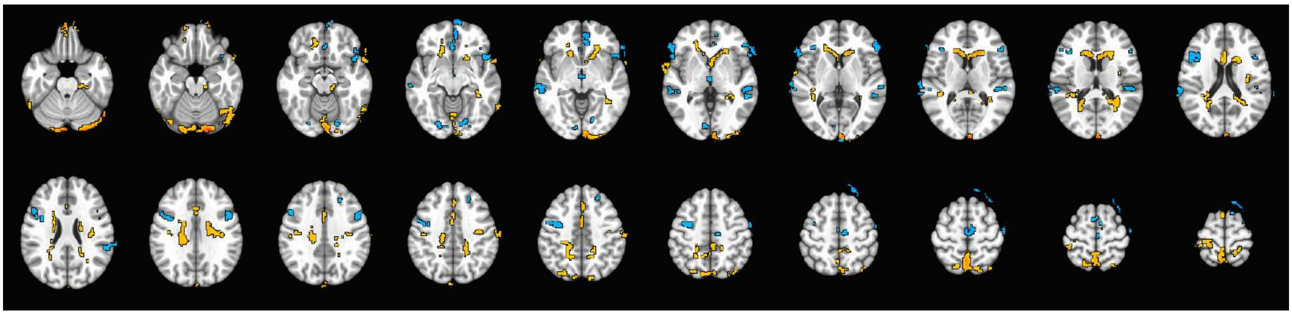

Figure S11.204

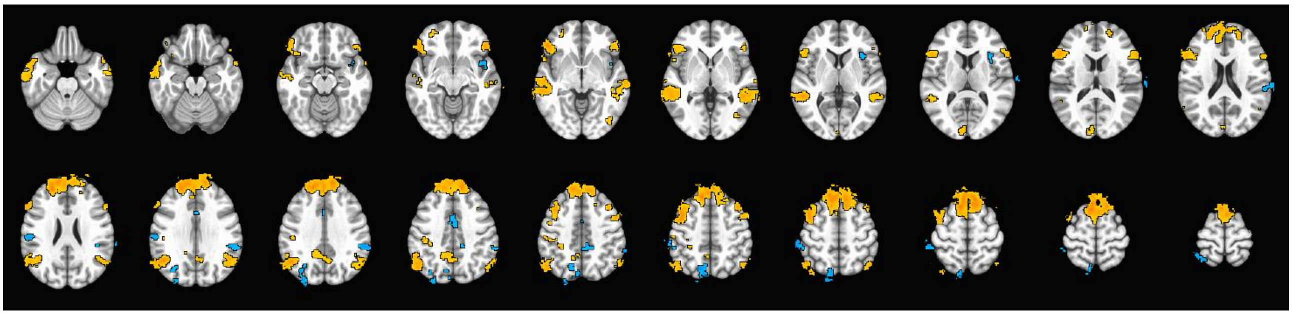

Figure S11.205

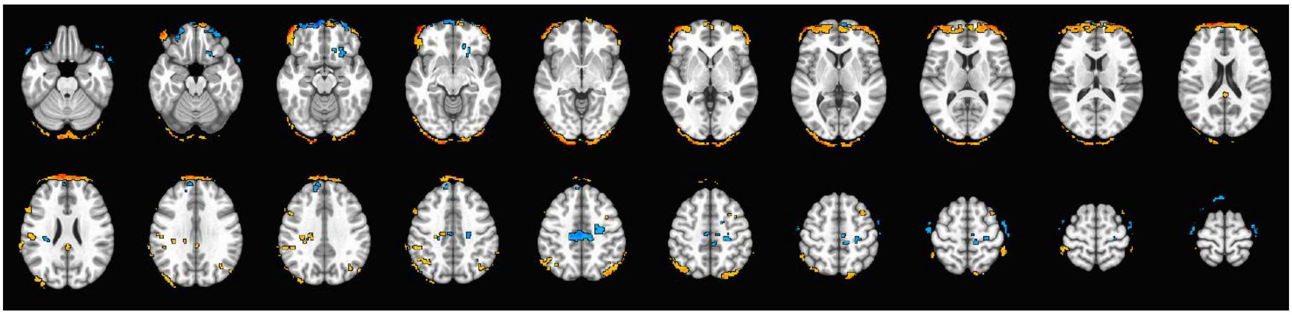

Figure S11.206

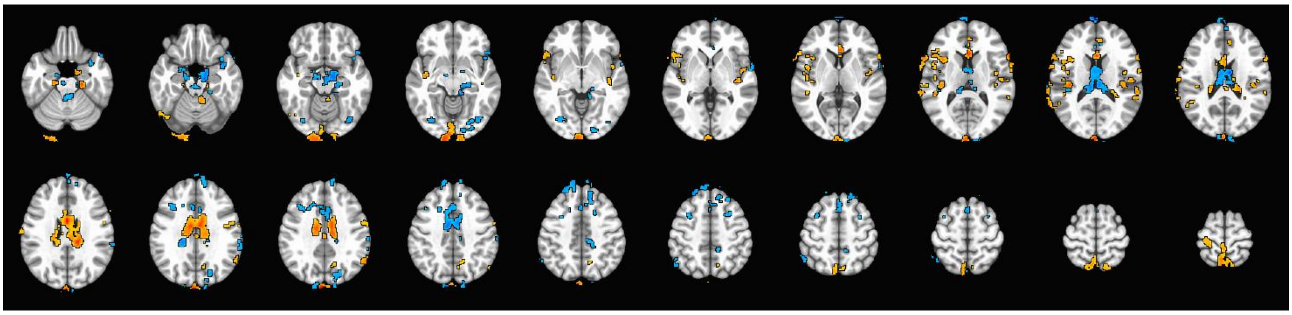

Figure S11.207

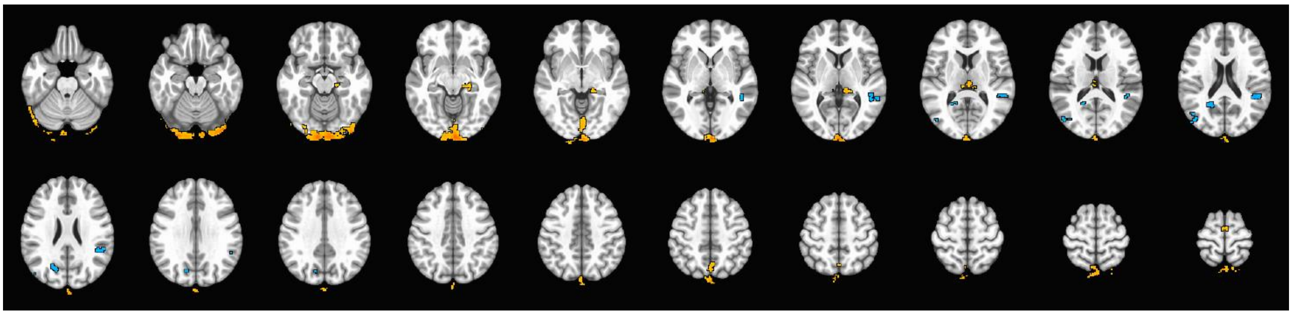

Figure S11.208

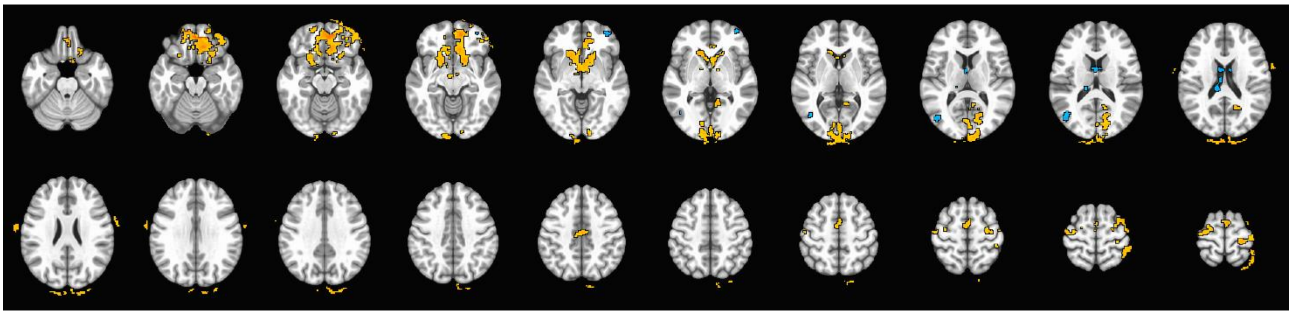

Figure S11.209

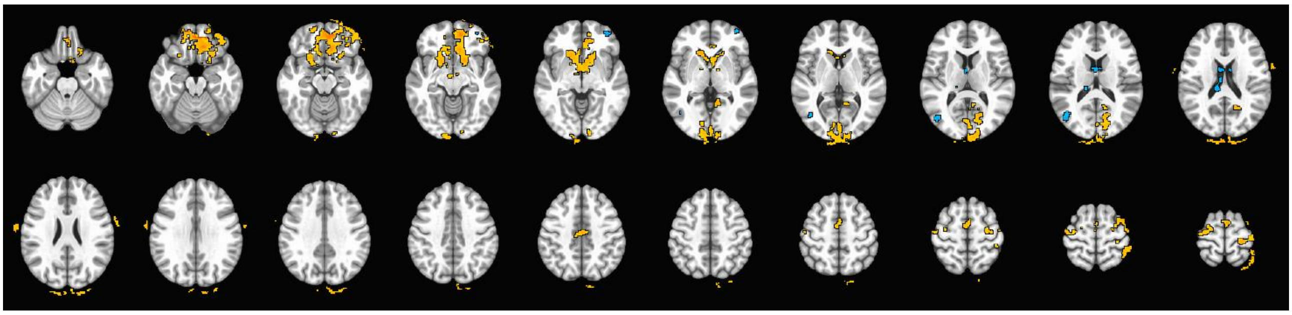

Figure S11.210

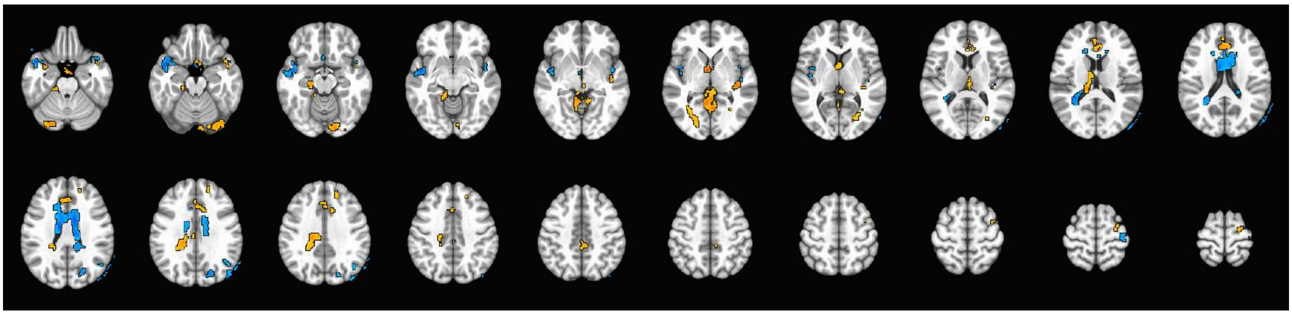

Figure S11.211

Group ICA at 7T with 4mm smoothing and Dimensionality 20

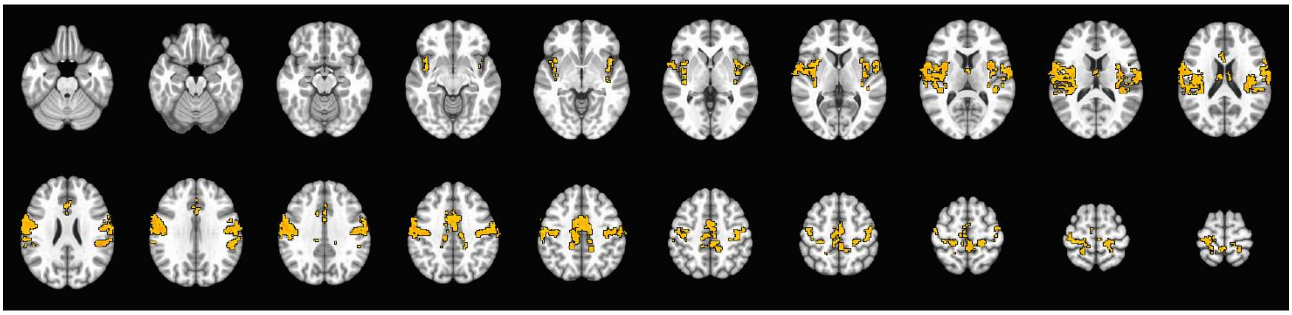

Figure S2122.1

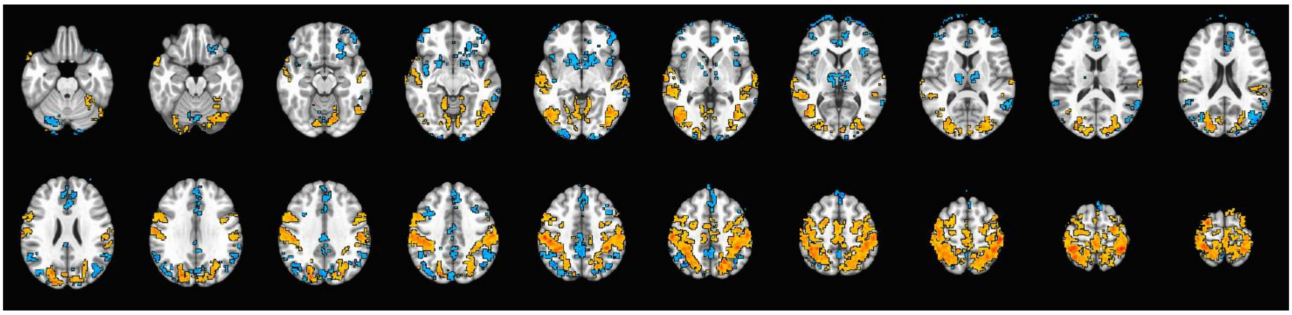

Figure S1.213

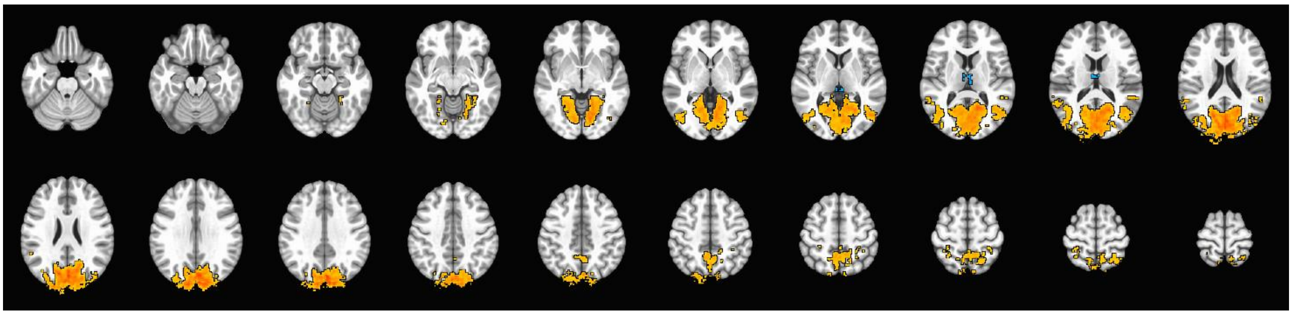

Figure S12.214

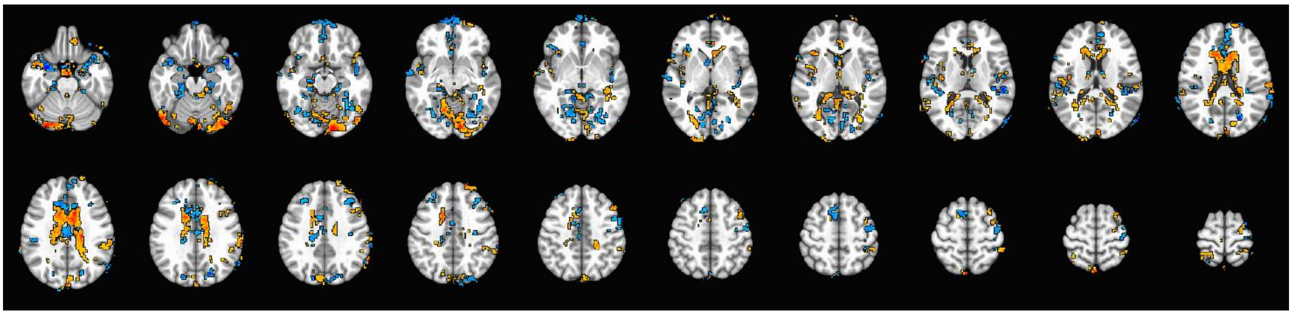

Figure S12.215

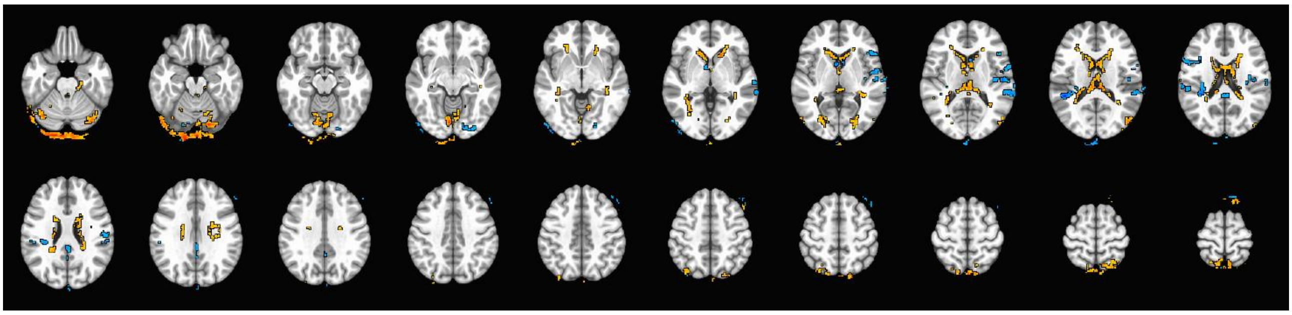

Figure S12.216

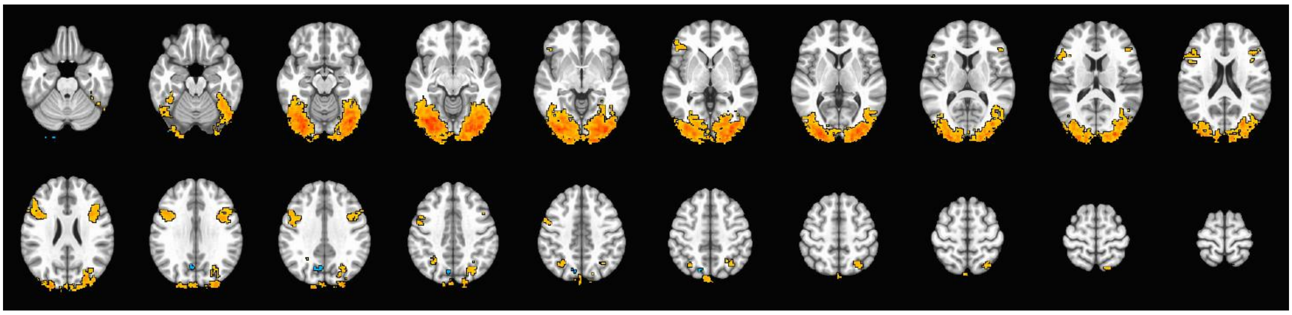

Figure S12.217

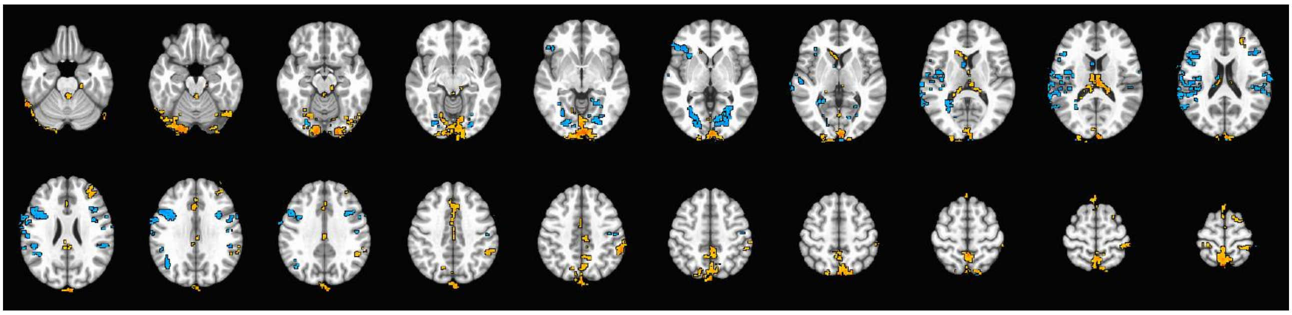

Figure S12.218

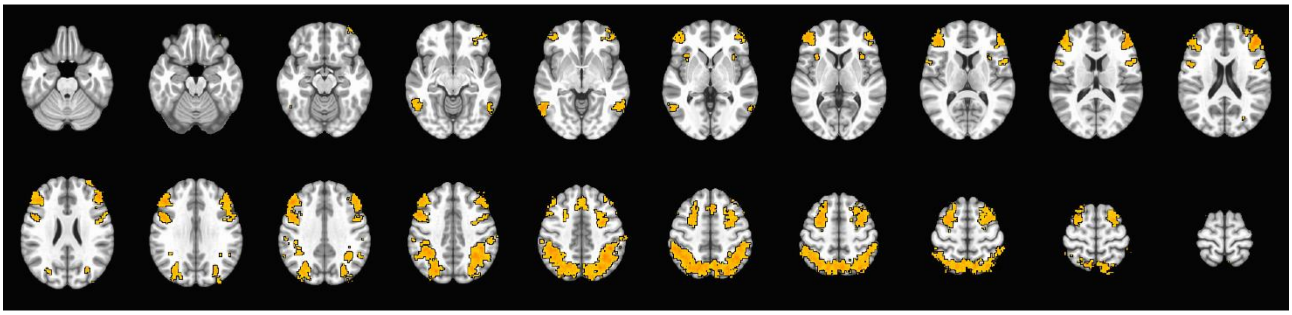

Figure S12.219

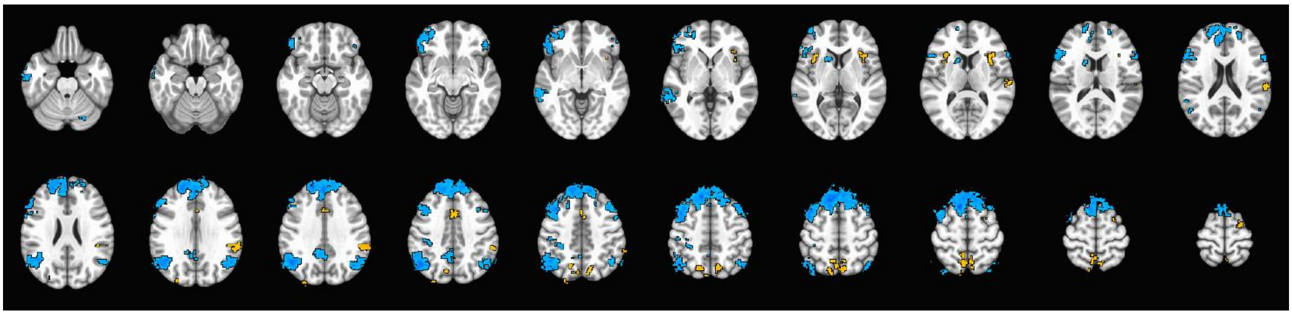

Figure S12.220

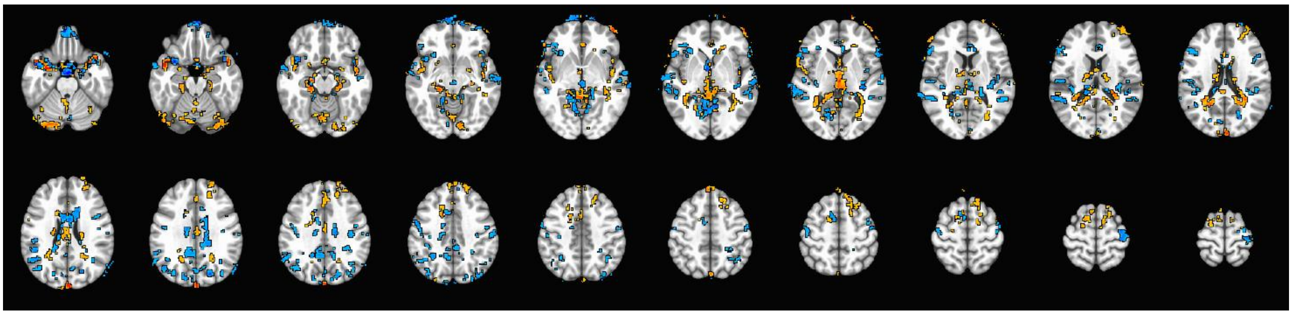

Figure S12.221

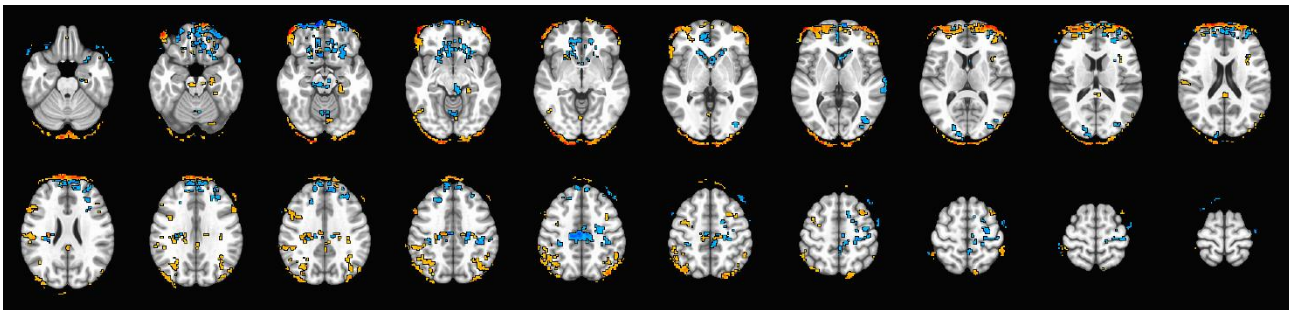

Figure S12.222

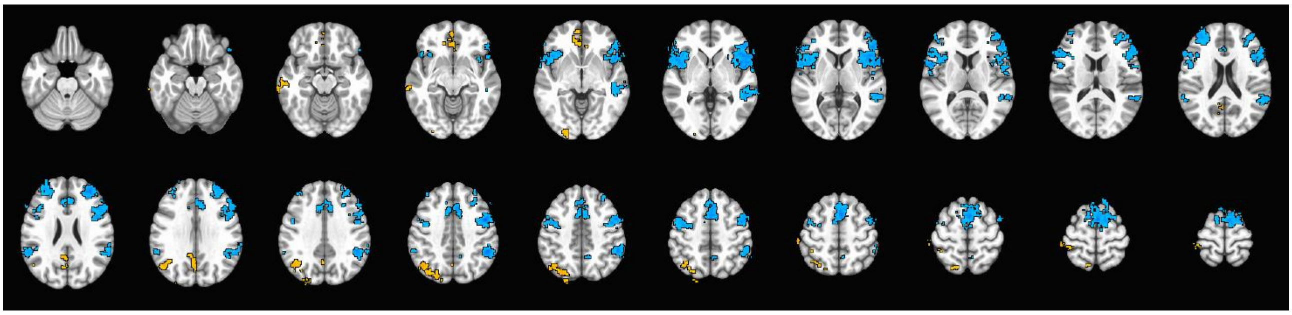

Figure S12.223

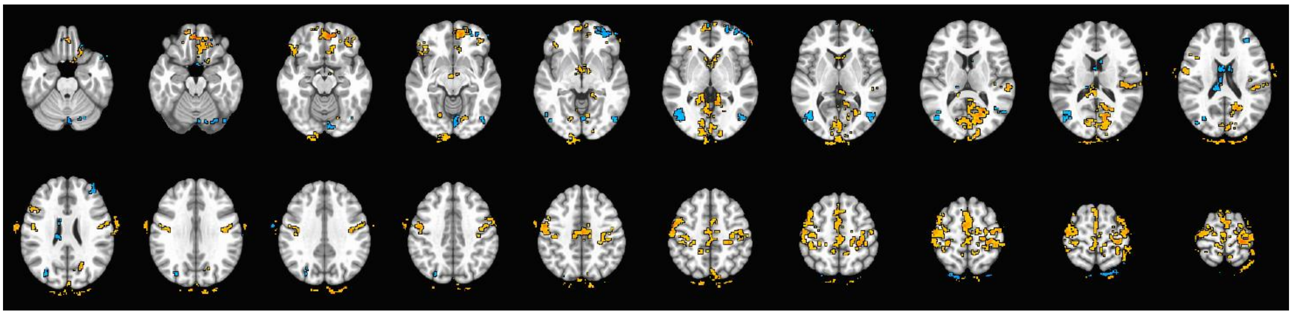

Figure S12.224

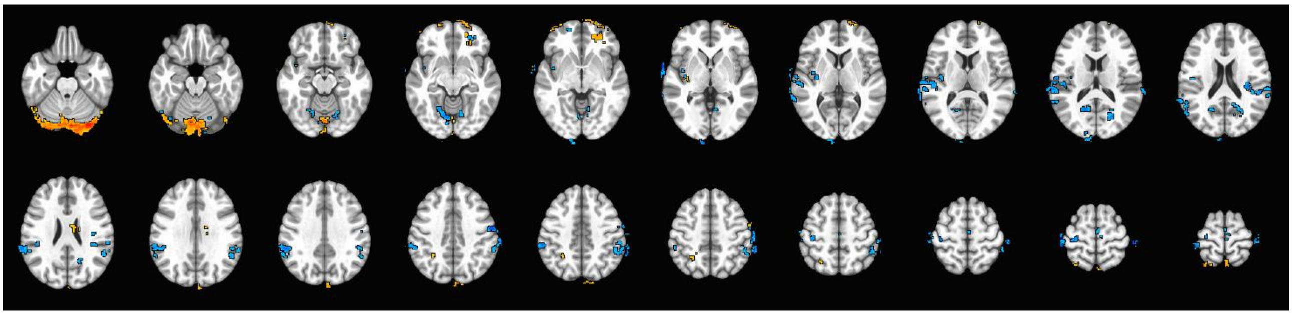

Figure S12.225

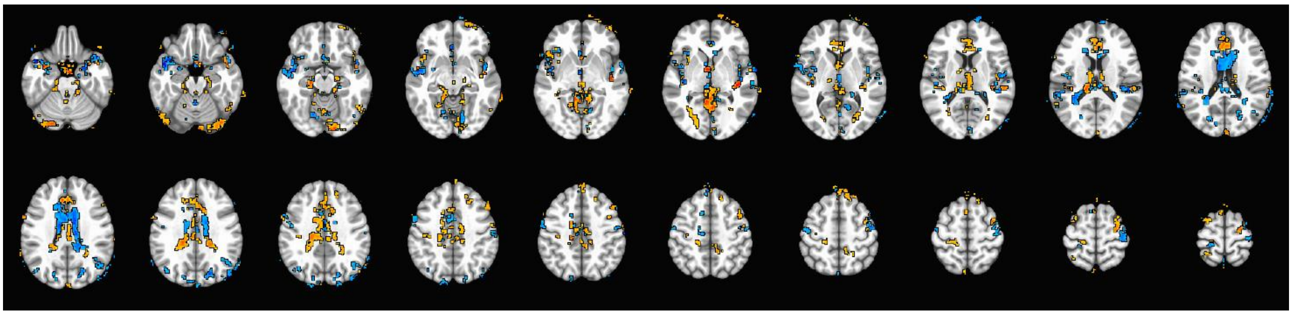

Figure S12.226

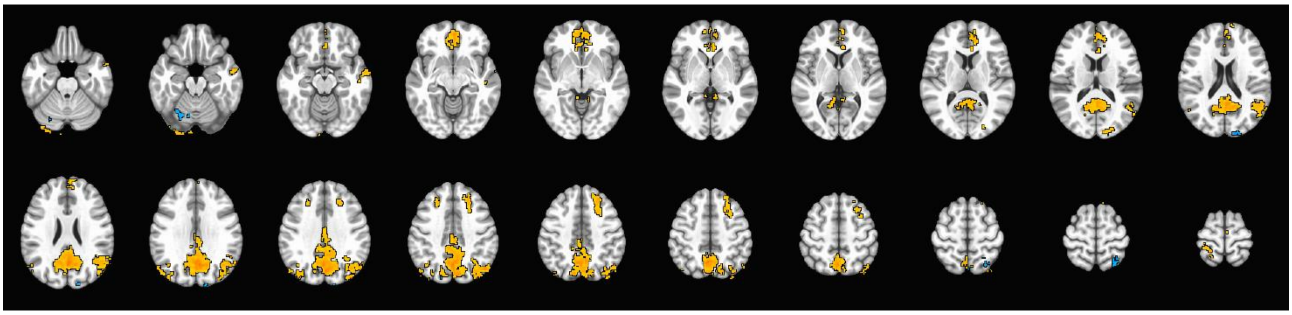

Figure S12.227

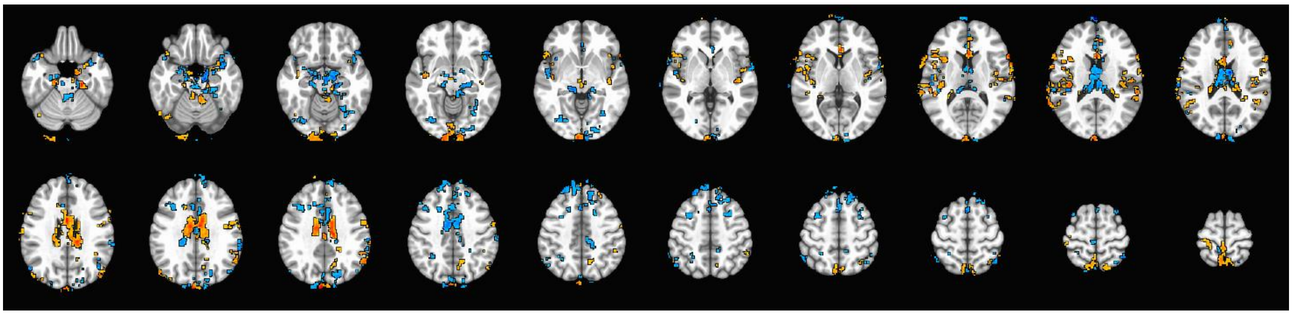

Figure S12.228

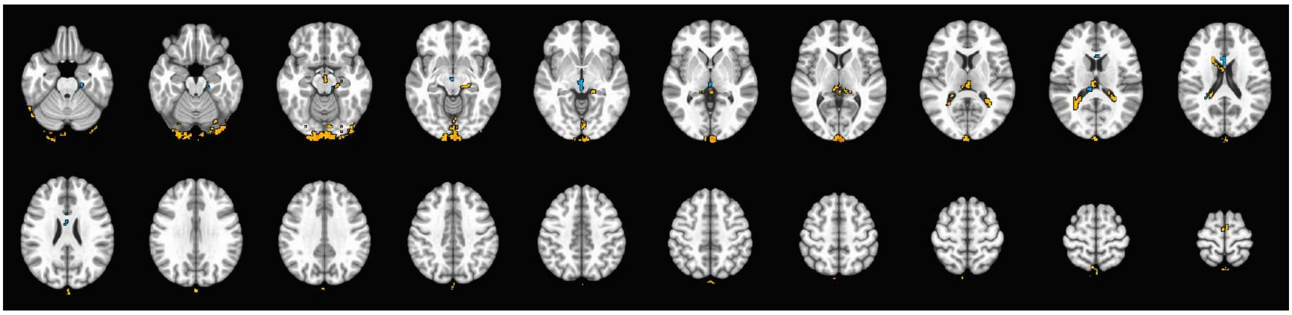

Figure S12.229

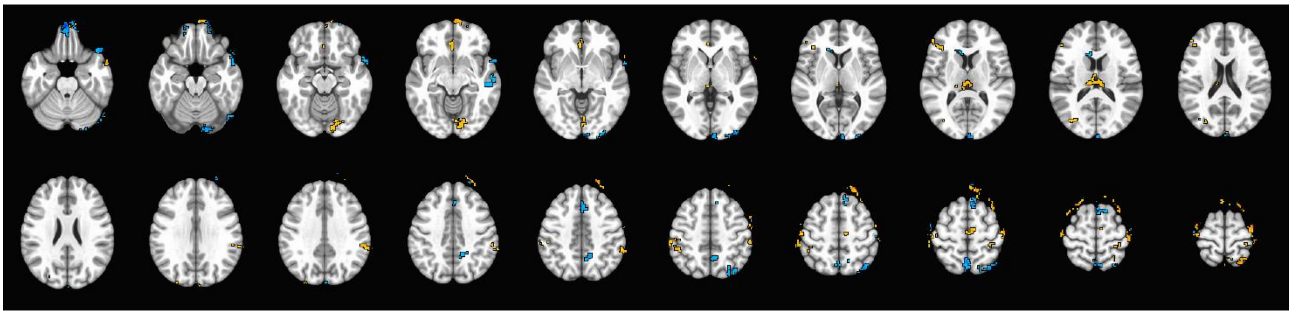

Figure S12.230

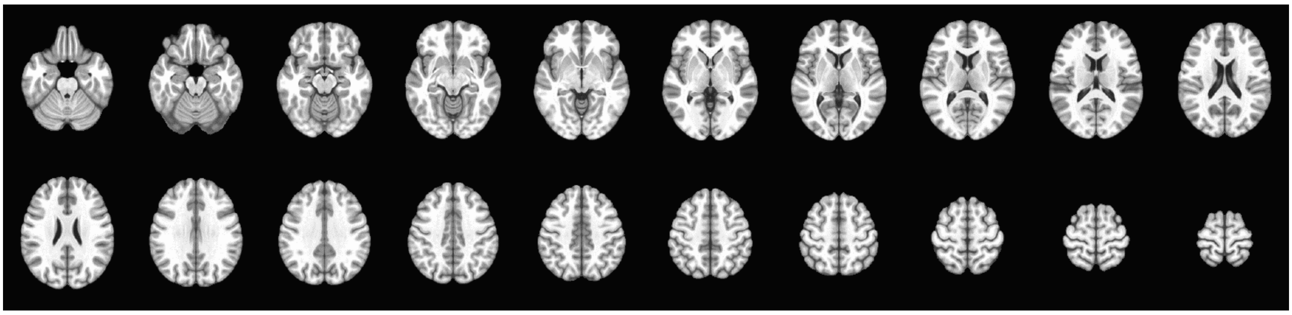

Figure S12.231
